# Novel motifs distinguish multiple homologues of *Polycomb *in vertebrates: expansion and diversification of the epigenetic toolkit

**DOI:** 10.1186/1471-2164-10-549

**Published:** 2009-11-20

**Authors:** Ramamoorthy Senthilkumar, Rakesh K Mishra

**Affiliations:** 1 Centre for Cellular and Molecular Biology, Uppal Road, Hyderabad 500007, India; 2 Centre for Computational Natural Sciences and Bioinformatics, International Institute of Information Technology, Hyderabad 500032, India

## Abstract

**Background:**

*Polycomb *group (PcG) proteins maintain expression pattern of genes set early during development. Although originally isolated as regulators of homeotic genes, PcG members play a key role in epigenetic mechanism that maintains the expression state of a large number of genes. *Polycomb *(PC) is conserved during evolution and while invertebrates have one PC gene, vertebrates have five or more homologues. It remains unclear if different vertebrate PC homologues have distinct or overlapping functions. We have identified and compared the sequence of PC homologues in various organisms to analyze similarities and differences that shaped the evolutionary history of this key regulatory protein.

**Results:**

All PC homologues have an N-terminal chromodomain and a C-terminal *Polycomb *Repressor box. We searched the protein and genome sequence database of various organisms for these signatures and identified ~100 PC homologues. Comparative analysis of these sequences led to the identification of a novel insect specific motif and several novel and signature motifs in the vertebrate homologue: two in CBX2 (Cx2.1 and Cx2.2), four in CBX4 (Cx4.1, Cx4.2, Cx4.3 and Cx4.4), three in CBX6 (Cx6.1, Cx6.2 and Cx6.3) and one in CBX8 (Cx8.1). Additionally, adjacent to the chromodomain, all the vertebrate homologues have a DNA binding motif - AT-Hook in case of CBX2, which was known earlier, and 'AT-Hook Like' motif, from this study, in other PC homologues.

**Conclusion:**

Our analysis shows that PC is an ancient gene dating back to pre bilaterian origin that has not only been conserved but has also expanded during the evolution of complexity. Unique motifs acquired by each homologue have been maintained for more than 500 millions years indicating their functional relevance in boosting the epigenetic 'tool kit'. We report the presence of a DNA interaction motif adjacent to chromodomain in all vertebrate PC homologues and suggest a three-way 'PC-histoneH3-DNA' interaction that can restrict nucleosome dynamics. The signature motifs of PC homologues and insect specific motif identified in this study pave the way to understand the molecular basis of epigenetic mechanisms.

## Background

Cell type specific expression pattern of genes is set during development. This complex process is accompanied by differential packaging of the genome in a cell and tissue specific manner that involves post-translational modifications, like methylation and acetylation of histones, and subsequent interaction of other regulatory proteins. The differential organization of chromatin, once set early during development, is maintained by *Polycomb *group (PcG) and trithorax group (trxG) proteins. Maintenance of chromatin structure, and thereby the expression state, is referred to as epigenetic cellular memory that provides continuity of specific pattern of expression states in daughter cells when a differentiated cell divides and also throughout the life span of organisms. PcG proteins maintain the repressed state, while trxG proteins maintain genes in the active state. Both PcG and trxG proteins function as multi-protein complexes. In *Drosophila*, PcG proteins form two major complexes. PC, Ph, Psc and dRing form *Polycomb *Repressive Complex1 (PRC1) [1], while PRC2 consists of Esc, E(z), Su(z)12 and P55 [2,3]. It has been observed that PCL also interact with a subset of PRC2, making a highly active and distinct complex [4,5]. A third complex consists of a DNA binding protein Pleiohomeotic (PHO) that interacts with PC and directs its binding to the specific sites of recruitment [6]. Similarly, trxG proteins form specific complexes [7]. The DNA sequences that function as sites for the recruitment of the PcG/trxG proteins are called the cellular memory elements or Polycomb response elements (PREs) [8]. Often common elements function to recruit both PcG and trxG proteins. It is generally believed that a balance between the two opposing functions on PREs maintains the precise level of expression state of a particular genomic region. Expression state of the locus is interpreted and maintained by distinct set of PcG and trxG complexes that bind to PREs and establish a chromatin state marked by specific histone modifications [9-14].

PC, the core member of PRC1, was first identified in *Drosophila*. The PC mutation causes a dominant phenotype of extra sex comb and is an essential gene. Many members of this group show similar phenotype due to the defect in the expression pattern of homeotic genes [15]. In *Drosophila*, the initial expression of homeotic genes is determined by segmentation genes [16] and subsequently this expression pattern is maintained by PcG and trxG proteins [17]. Changes in the expression pattern leads to homeotic transformation and/or lethality [18]. Insects have one PC gene, where as mammals have five homologues. Vertebrate homologues of PC contain Chromatin organizer modifier domain, chromodomain, and are referred to as chromobox, CBX, proteins. These include CBX2, CBX4, CBX6, CBX7 and CBX8. The other CBX proteins CBX1, CBX3 and CBX5 are the homologues of heterochromatin protein (HP1). Here we refer PC proteins of vertebrates as CBX proteins.

Several lines of evidence suggest that homeotic genes are not the only targets of PcG genes [19,20]. More recently, genome wide ChIP on Chip analysis of PcG proteins and its associated histone methylation marks in fly, human and mouse cells have identified large number of targets of these proteins [21-23]. PcG members are essential for maintenance and normal proliferation of cells and have been implicated in the maintenance of stem cells [24]. Genome wide mapping of H3K27Me3 in various prostate cancer tissues shows the PcG mediated repression of several genes which are down regulated in cancer [25]. The abnormal expression of PcG genes cause misregulation on its target loci and subsequently to abnormal proliferation of cells and cancer [24,26]. CBX7 and CBX8 are involved in maintaining the repressive state of *INK4A-ARF *locus which is involved in the regulation of cellular proliferation and senescence [27,28]. CBX7 knockdown increases the ARF and INK4A expression which causes impairment in cell growth [29]. CBX4 is the repressor of C-MYC and mutation in its C-terminal region leads to enhanced expression of this proto oncogene and cellular transformation [30]. Genome wide mapping of CBX8 target shows that this PcG protein is predominantly associated with genes that are involved in developmental and differentiation processes [31]. CBX2 and CBX7 have also been implicated in maintenance of the inactive X-chromosome in mouse [32,33].

The N-terminal end of PC has chromodomain, which binds to the histone methylation marks created by PRC2 on PREs [34]. Chromodomain is a three beta strands and a helix containing domain present in proteins that are involved in chromatin organization, viz., HP1, SU(var)3-9, Swi6, CHD1, MSL-3, MOF, etc. Chromodomain is involved in targeting the protein to specific regions of chromatin. The chromodomain of *Drosophila *PC exhibits preferential binding to tri-methylated histone H3 at lysine 27 (H3K27Me3) [35] whereas chromodomain of HP1 recognizes H3K9Me3 mark. Mutation in the chromodomain of PC results in the disintegration of PRC1 and subsequently loss of its silencing activity [36]. In *Drosophila*, a chimeric protein generated by replacing chromodomain of HP1 with PC chromodomain localizes HP1 to euchromatic PC binding sites indicating that chromodomain is essential for recognizing specific histone methyl marks [37,38]. This suggests that subtle differences in the sequence/structure of chromodomains may confer differential affinity to different histone methylation patterns, for example, H3K9Me3 and H3K27Me3. Unlike the fly PC that recognizes only H3K27Me3, mammalian PC homologues show differential binding to methylated histone. CBX2 and CBX7 bind to both H3K9Me3 and H3K27Me3 whereas CBX4 shows strong affinity for H3K9Me3 [33].

Significance in PcG system is apparent from the observation that these genes are not only conserved from plants to animals, but also highly evolved animals have more homologues of PcG genes. For example, while insects have only one copy of PC, vertebrates have at least five homologues. The importance of having more PC homologues in an organism remains elusive. Identification of uniquely conserved regions in each CBX protein will help us understand the function of homologues. In this study, we carried out extensive mining and analysis of PC homologues to understand their evolution and sequence-structure-function relationship in the context of motif organization.

## Results

### Search for PC homologues and comparative sequence analysis

**Figure 1:**
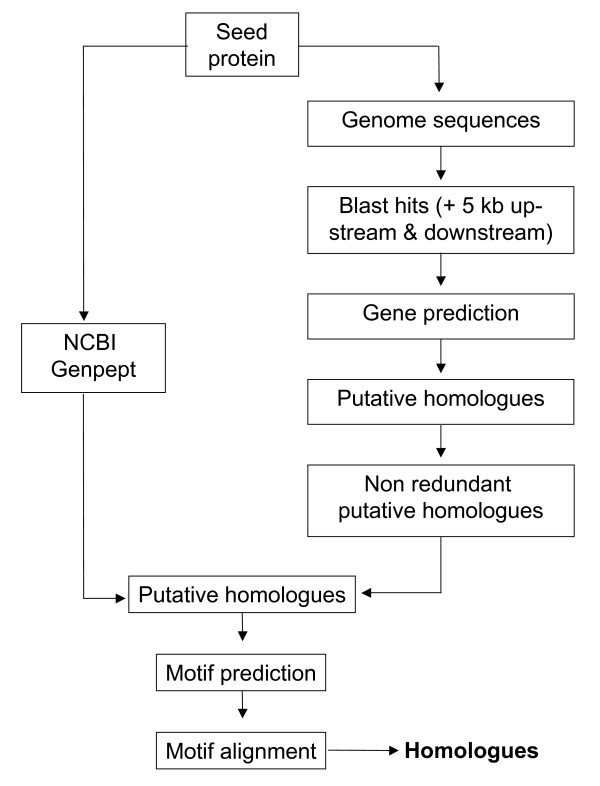
**Schema for mining the homologues sequences**. The schema represents the approach followed for mining PC homologues. The seed sequence, PC of *Drosophila *was searched in the NCBI protein sequence database and the genome sequences of the model organisms. The TBLASTN hits of genome sequences were extended 5 kb upstream and 5 kb downstream and merged. The extended hits were subjected to gene prediction and the sequences showing >98% similarity were considered as redundant records and the non redundant records were considered as putative homologues and subjected to motif predictions. Sequences were grouped based on the motif conservation.

**Figure 2:**
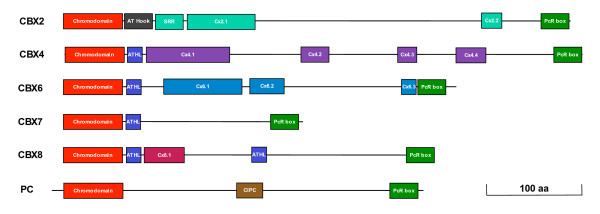
**Conserved regions of PC homologues**. The conserved regions are mapped to the scale in fly and human homologues. Each novel motif identified is named based on the name of the homologue and the order it occurs in the protein. SRR-serine rich region; CIPC-Conserved region of insects PC; PcR box - *Polycomb *Repressor box; ATHL - AT-Hook Like, a conserved motif present in CBX4, CBX6, CBX7 and CBX8; Cx2.1 and Cx2.2 - Conserved Region (CR) of CBX2; Cx4.1, Cx4.2, Cx4.3 and Cx4.4 - CR of CBX4; Cx6.1, Cx6.2 and Cx6.3 - CR of CBX6; Cx8.1 - CR of CBX8.

**Figure 3:**
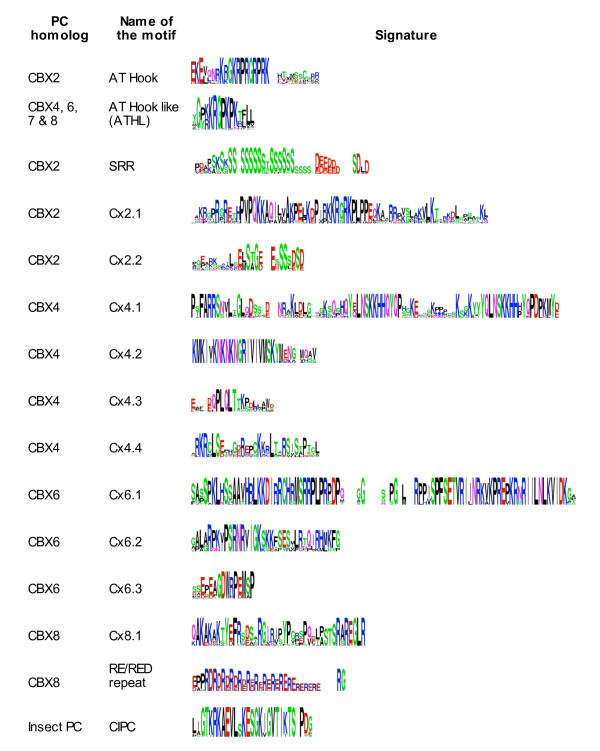
**Novel, conserved and CBX specific motifs**. The conserved motifs present between chromodomain and PcR box are listed. Each row represents name of the motif, name of homologue having the motif and the amino acid conservation pattern in logo format. The height of amino acids is proportional to the frequency of occurrence (degree conservation) in each position. The MSA of motifs is in the Additional file 5.

All PC homologues have N-terminal chromodomain and a conserved *Polycomb *repressor box, PcR box, at the C terminus. We used these features to mine PC homologues in the protein sequence database and genome sequence of different organisms and identified 100 PC homologues (Figure 1, see Additional file 1). Barring the two known domains, PC homologues have not been shown to contain any other distinguishable molecular or structural features. We carried out secondary structure analysis of these proteins and found that the region between chromodomain and PcR box is predominantly predicted as coils (see Additional file 2). Such secondary structures are known to be involved in protein-protein interactions and acquire specific structural features as a consequence of such interactions. Since PC homologues show poor sequence homology in this region, we used motif prediction and motif alignment tools to find additional conserved regions that might be present in various PC homologues (see Additional files 3 and 4). This approach led to the identification of a number of highly conserved motifs that are conserved across vertebrates (Figures 2 &3, see Additional file 5). Most of the motifs identified in this study are novel. Regions that are conserved in all PC homologues and uniquely conserved in each CBX protein are shown in Figure 2. Partial sequences of potential PC homologues present in unfinished databases have been excluded from the analysis.

### Chromodomain in PC homologues

**Figure 4:**
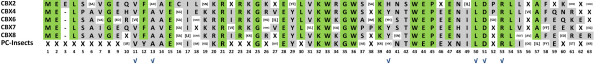
**Consensus sequences of chromodomain of Polycomb homologues**. Represented in MSA format, the consensus of each CBX protein and the consensus pattern of insect PC are shown in the alignment. The residues conserved across the homologues are highlighted in green and residues that are selectively conserved in one or more proteins are highlighted in gray. √ - The key residues of *Drosophila *PC for H3K27Me3 interaction and PC dimerization.

**Figure 5:**
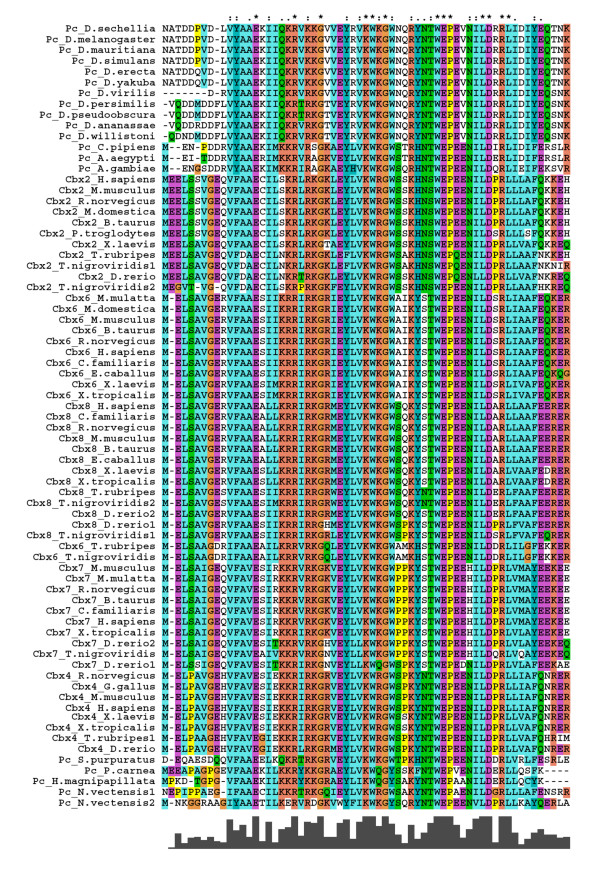
**The multiple sequence alignment of chromodomain of Polycomb homologues**. Sequences are named with protein and species name. The amino acids are highlighted in different colours based on their properties. The degree of conservation observed at each position is represented as bar graph in the bottom. The duplicated fish homologues are represented with a number following to the species name.

**Figure 6:**
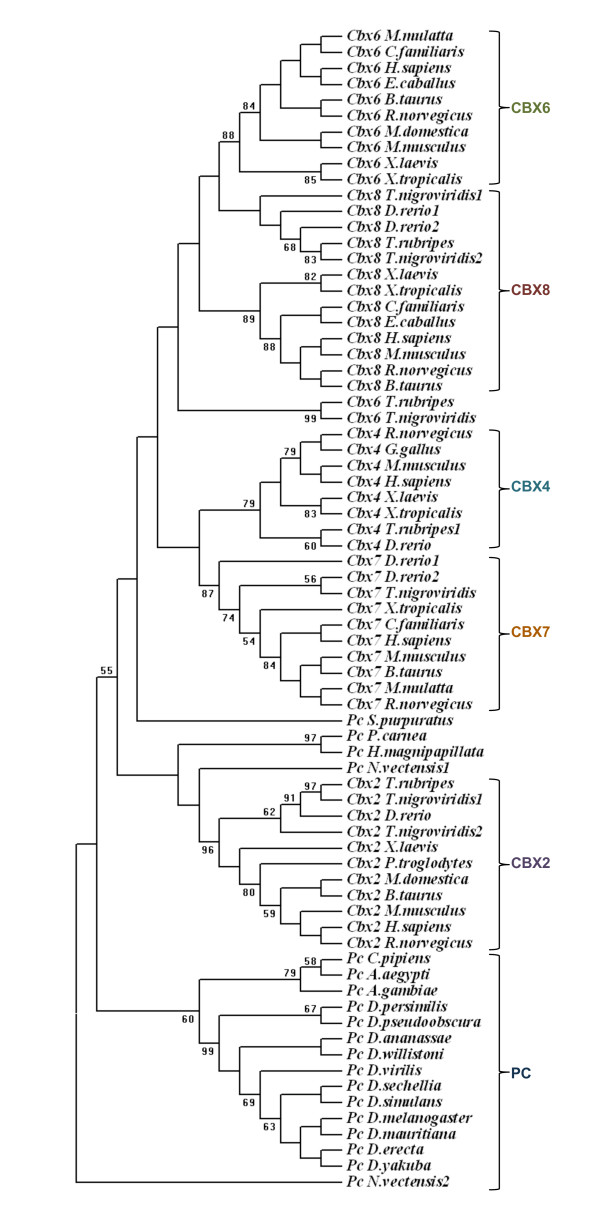
**The phylogenetic tree constructed using chromodomain of Polycomb homologues**. The phylogenetic tree was constructed using neighbour joining method. The value of bootstrap consensus (>50%) for 1000 replicates is shown in the branches. The sequences are named with the homologue name and species name. The duplicated fish homologues are represented with a number following to the species name.

Even though highly conserved, chromodomains of different kinds of chromatin proteins contain subtle variations that specify recognition of distinct methylation patterns of histones. We analysed the chromodomain of different PC homologues to understand the sequence determinants of their possible functional differences (Figures 4 &5). We find that all PC homologues have *Polycomb *type chromodomain, and not like the one of HP1, with key amino acids being conserved and that chromodomains of different PC homologues have characteristic sequence features that are highly conserved. For example, CBX2 chromodomain among the vertebrates shows more similarity and is relatively distant from that of CBX4, 6, 7 or 8. Using chromodomain of *Polycomb *homologues collected in this study, we constructed a phylogenetic tree. The chromodomain of CBX2, 4, 6, 7 and 8 from different vertebrates fall in distinct clusters and so do the chromodomains of invertebrate PC albeit in yet another cluster (Figure 6). This clearly shows that chromodomains of different PC paralogue groups have distinct features. The amino acids that define such distinct features may be essential for directing the paralogues to different targets or functions. It also suggests that different homologues in the common ancestor of vertebrates had acquired unique features that have been in similar selection pressure. We also noticed that the chromodomain of CBX proteins is distributed in 3 exons in all vertebrates indicating that one domain can be distributed in more than one exon.

The crystal structure of fly PC chromodomain indicates that W(36) and Y(40) residues are essential for its interaction with H3K27Me3 [39]. W is involved in H3K27Me3 recognition and Y is involved in stabilization of the interaction. W is conserved in all the PC homologues whereas Y is not conserved as some homologues have His residue at this position (Figures 4 &5). The *Drosophila *PC chromodomain also forms hydrophobic pocket, involving V(11), Y(12) and A(13), that is essential for its interaction with H3. CBX6 has V or I at residue 11 whereas the other homologues have highly conserved V at this position. Similarly, A(13) is replaced by other residues only in CBX2. Y(12) is conserved across insects whereas vertebrate homologues have F at this position. D(51) and R(53), also involved in PC and H3 interaction, are conserved across the species. PC chromodomain functions as dimer [35,39]. Of the two key residues shown to be essential for PC dimerization, L(50) is conserved while R(52) is not. These important differences between insect PC chromodomain and among that of vertebrates, point to different preferences for histone modifications by the vertebrate PC homologues. The residues essential for differential affinity of PC homologues to different methylation marks, however, remain elusive. Interestingly we find several residues that are uniquely conserved in paralogue specific manner (Figure 4, highlighted in gray). Further studies will be needed to understand functional significance of these residues.

### *Polycomb *repressor (PcR) domain

**Figure 7:**
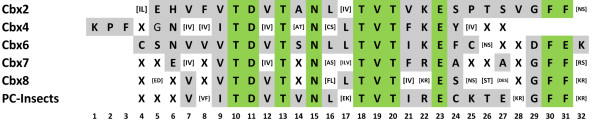
**Consensus sequences of PcR box of Polycomb homologues**. Represented in MSA format, the PcR box consensus of each CBX protein and the consensus pattern of insect PC are shown in the alignment. The conserved residues are highlighted in green and residues that are specifically conserved in one or more CBX proteins are highlighted in gray.

**Figure 8:**
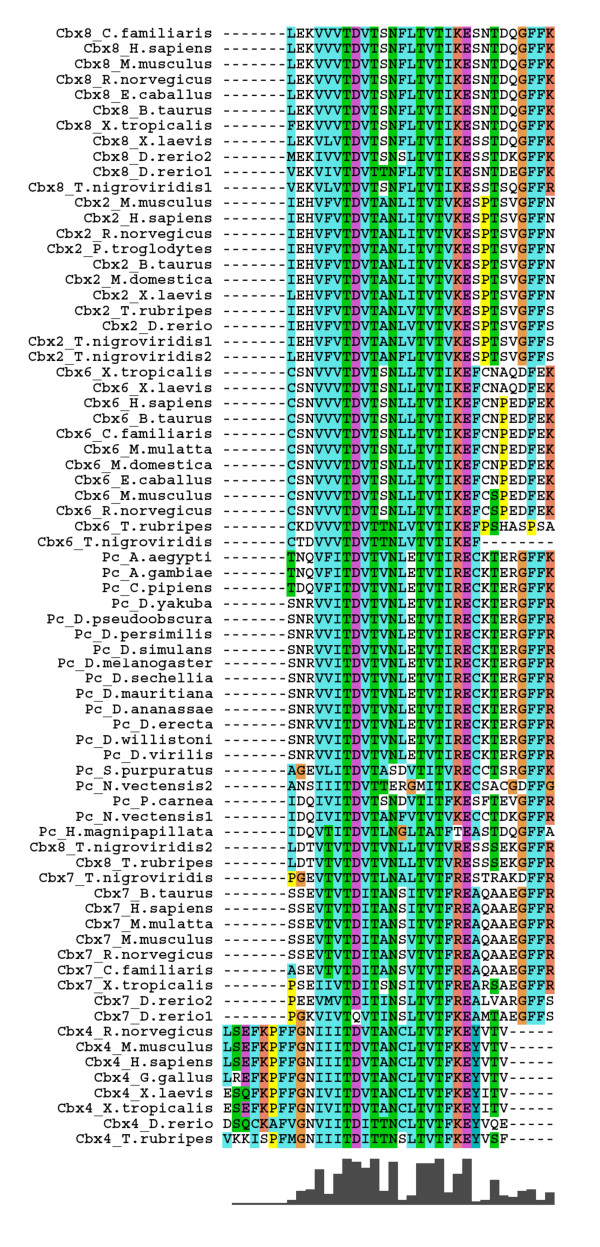
**The multiple sequence alignment of PcR box of Polycomb homologues**. Sequences are named with protein name and species name. The amino acids are highlighted in different colours based on their properties. The degree of conservation observed at each position is represented as bar graph in the bottom. The duplicated fish homologues are represented with a number following to the species name.

**Figure 9:**
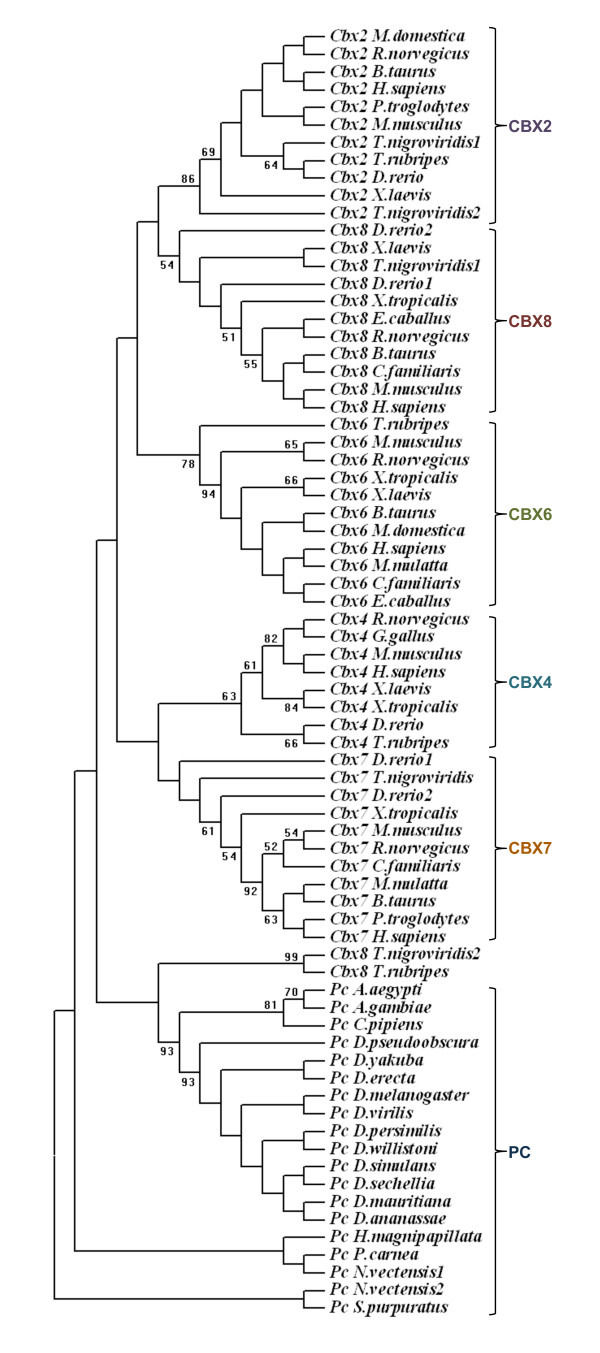
**The phylogenetic tree constructed using PcR box of Polycomb homologues**. Phylogenetic tree was constructed using neighbour joining method. The value of bootstrap consensus (>50%) for 1000 replicates is shown in the branches. The sequences are named with the homologue name and species name. The duplicated fish homologues are represented with a number following to the species name.

The C-terminal region is responsible for the repressive role of PC protein [30,40-43] and is, therefore, referred to as *Polycomb *Repressor (PcR) box. PcR box shows high degree of conservation in all homologues and is unique to *Polycomb *proteins (Figures 7 &8) unlike chromodomain that is found in several other chromatin associated proteins. Deletion of this region in fly shows loss of PC function [40], and has been shown to be essential for interaction of PC homologues CBX2 and CBX8 with RING1A which is a homologue of fly dRING and a component of PRC1 [43,44]. The phylogenetic tree constructed based on the multiple sequence alignment (MSA) of PcR box shows that this motif has inherent homology pattern specific to each CBX (Figure 9), an observation parallel to that in case of chromodomain. A consensus pattern was drawn for each CBX protein and insect PC to look for the residues that are conserved and those that are specific to certain CBX proteins (Figure 7). The sixth amino acid residues in MSA of consensus pattern shows diversity with CBX4 and CBX6 having N, CBX2 having H and CBX7 having E at this position. Likewise, at residue 9, CBX4 and PC of insects have I where as other proteins have V at this position. At position 24, CBX2 and CBX8 have S whereas CBX4, CBX6, CBX7 and insects PC have Y, F, A and C, respectively. These observations indicate that CBX specific alterations occurred early in the vertebrate ancestor and have since been conserved. Such conserved residues may, therefore, play an essential role in differential interaction of PC and RING homologues or other functions that specify their distinct roles.

### AT-Hook and AT-Hook Like motifs in PC homologues

CBX2 has a conserved AT-hook motif adjacent to N-terminal chromodomain. This motif is present in many DNA binding proteins and interacts with the minor groove of AT rich region and enhances the interaction of the protein with chromatin and/or DNA [45]. In CBX4, 6, 7 & 8 we found a highly conserved motif with close similarity to AT-Hook and named it as 'AT-Hook Like' (ATHL) motif. Like AT-Hook, this motif is also not specific to Cbx proteins and found in several other nuclear proteins that are likely to interact with DNA (see Additional file 6). The core region of AT-Hook motif has highly conserved P, G and two basic amino acids, K and R. Similarly the ATHL motif is also rich in basic amino acids and has P and G in the core region (Figure 3, see Additional file 5). This indicates a possible alternative to the AT-Hook in other CBX protein for their chromatin binding. Interestingly, except in *Xenopus*, CBX8 has two ATHL motifs. Although the functional importance of this motif in PC homologues is not clear yet, it is tempting to suggest that it may be involved in a cooperative three way interaction: "H3K27Me3_chromodomain+AT-Hook/ATHL_DNA" on the nucleosome (see below).

### Novel motifs in PC homologues

The region between chromodomain and PcR domain of *Polycomb *homologues gives uniqueness to each CBX protein as it contains motifs that are specific to each homologue. In our analysis we found several such additional conserved motifs in this region (Table 1, Figure 3, see Additional files 3, 4 &5). We suggest that these domains confer unique and essential functionality to these proteins that allowed their conservation during the evolution from fish to human spanning over 500 million years.

**Table 1:** The conserved motifs of PC homologues

Conserved motif	Present in	Size(aa)	Secondary structure	Function
**Chromodomain**	All	61	Twisted antiparallel β sheet (3 β strands and a helix)	Binds with H3K27me3

***Polycomb *Repressor** **(PcR) box**	All	29-31	β strand	Interacts with dRING

**AT-Hook**	CBX2	29	Coils	DNA binding

**AT-Hook Like** **(ATHL)**	CBX4, CBX6, CBX7 and CBX8	15	Coils	Present in several nuclear proteins (DNA binding?)

**CX 2.1**	CBX2	69-74	Coils	-

**CX 2.2**	CBX2	25	Coils	-

**CX 4.1**	CBX4	84-88	Coils	-

**CX 4.2**	CBX4	29	Coils	-

**CX 4.3**	CBX4	20	Coils	SUMO binding

**CX 4.4**	CBX4	31	Coils	-

**CX 6.1**	CBX6	82-88	Coils	-

**CX 6.2**	CBX6	36	Coils	-

**CX 6.3**	CBX6	15	Coils	-

**CX 8.1**	CBX8	39-42	Coils	-

**Poly histidine site**	Insect PC CBX4 of mammals	6-11 10-14	Coils	-

**RED repeat**	CBX8 of mammals	13-37	Coils	Present in several proteins

**CtBP interaction motif**	CBX4	5	Coils	CtBP binding

**Sumoylation site**	CBX4 (Except fishes)	4-5	Coils	Sumoylation

**Serine rich region** **(SRR)**	CBX2 CBX6	23-38 4-6	Coils	-

**Conserved** **Insect Pc box** **(CIPC)**	Insect PC	25-29	Coils	Present in insect *Polycomb*

The multiple sequence alignments of the conserved motifs are given in Additional file 5.

### CBX2 specific motifs

In addition to AT-Hook motif, CBX2 has three conserved motifs (Figure 2). A Serine rich region (SRR) is present next to the AT-Hook motif. SRR is found in proteins with wide range of function and thought to be the site for phosphorylation and associated functions [46]. The length of SRR has reduced during evolution from fish to human. Fishes have 24 Serine residues whereas human have a stretch of 16 serine residues (see Additional file 5). An exception is the CBX2 of *Tetraodon*, where one of the two homologues identified does not have SSR and the size of the motif in other homologue is smaller than that in human. Two more unique motifs present in CBX2 are named as Cx2.1 and Cx2.2. In the 74 aa long Cx2.1 motif, the highly conserved core is rich in basic residues and Proline. Cx2.2 is present in C terminal region; closer to PcR box and has highly conserved Serine residues and acidic amino acids D and E.

### CBX4 specific motifs

The CBX4 protein functions as a SUMO E3 ligase and is involved in SUMOylation of CtBP, SIP1, HIPK2, CTCF and Dnmt3a [47-50]. The CBX4 is highly conserved across vertebrates. It has four conserved motifs (Cx4.1, Cx4.2, Cx4.3 and Cx4.4) (Figure 2, see Additional file 5). Cx4.1, a 88aa conserved motif, has a duplicated and highly conserved shorter stretch of Y [QE]LNSKKHH [QHP]YQP separated by a stretch of poorly conserved 19aa residues (Figure 3). A SUMO binding site is present in motif Cx4.2 and the region flanking this site is rich in Lysine with a highly conserved (KN)3 repeat within it. The motif Cx4.3, which has highly conserved hydrophilic residues (Q, S and T), is absent in *Danio rerio *but present in *Fugu*. CBX4 has a conserved CtBP binding site and a SUMOylation site as well. These are absent in other PC homologues [51] which indicates the functional specificity of CBX4.

### CBX6 specific motifs

CBX6 has three conserved regions between chromodomain and PcR box (Figure 2, see Additional file 5). Cx6.1 is a 88 aa highly conserved region unique to CBX6 and is rich in S, T, N, P and basic amino acids. One end of the motif is rich in Serine, followed by basic amino acid rich region, Proline rich region and basic amino acid rich region, indicating possible multiple functional domains present within this motif. Cx6.2 is rich in basic amino acids, of the 36 residues 10 are basic amino acids. Cx6.3 is a small conserved motif present closer to PcR box and is rich in acidic amino acids and Proline.

### CBX8 specific motifs

CBX8 has one conserved motif unique to it, Cx8.1 following its first AT-Hook like motif (Figure 2, see Additional file 5). CBX8 of mammals also has a RED/RD repeats, present in several nuclear proteins although the function of this acid-base dipeptide repeat is not known (see Additional file 5). Unlike SRR repeat in CBX2, which shows reduction in the repeat length as we move from fish to human, the length of the RED repeat has expanded from mouse to human - mouse has 4 while human has 16 repeat units. In all the species studied, the repeat ends with RG residues. The less conserved region of CBX8 (conserved from *Xenopus *to mammals but not in fishes), 202-333 amino acids, was shown to interact with the nuclear protein AF9 in mouse [52]. Although the function of AF9 protein is not well understood, its human homologue, ENL, is involved in transcriptional activation [53] and interacts with CBX8 [54]. The AF9 locus is prone to gene rearrangement involving MLL and the MLL-AF9 fusion protein is associated with acute leukaemia in mice [52]. Further studies will be needed to understand the precise role of CBX8 in these events.

### Insect specific conserved region

In sequence comparison analysis along with the vertebrate homologues of PC, insect PC always clusters in one group indicating its highly conserved nature compared to the vertebrate counter parts. In this analysis we identified a novel insect specific highly conserved motif, conserved inset specific *Polycomb *(CIPC) box (Figure 2). This unique motif is mainly composed of basic, hydroxyl and acidic residues (see Additional file 5). Vertebrate homologues of PC do not have this extremely conserved insect specific motif. It appears as though CIPC was replaced by different conserved motifs that are specific to each homologue in order to acquire functional uniqueness.

### Evolution of *Polycomb *homologues

**Figure 10:**
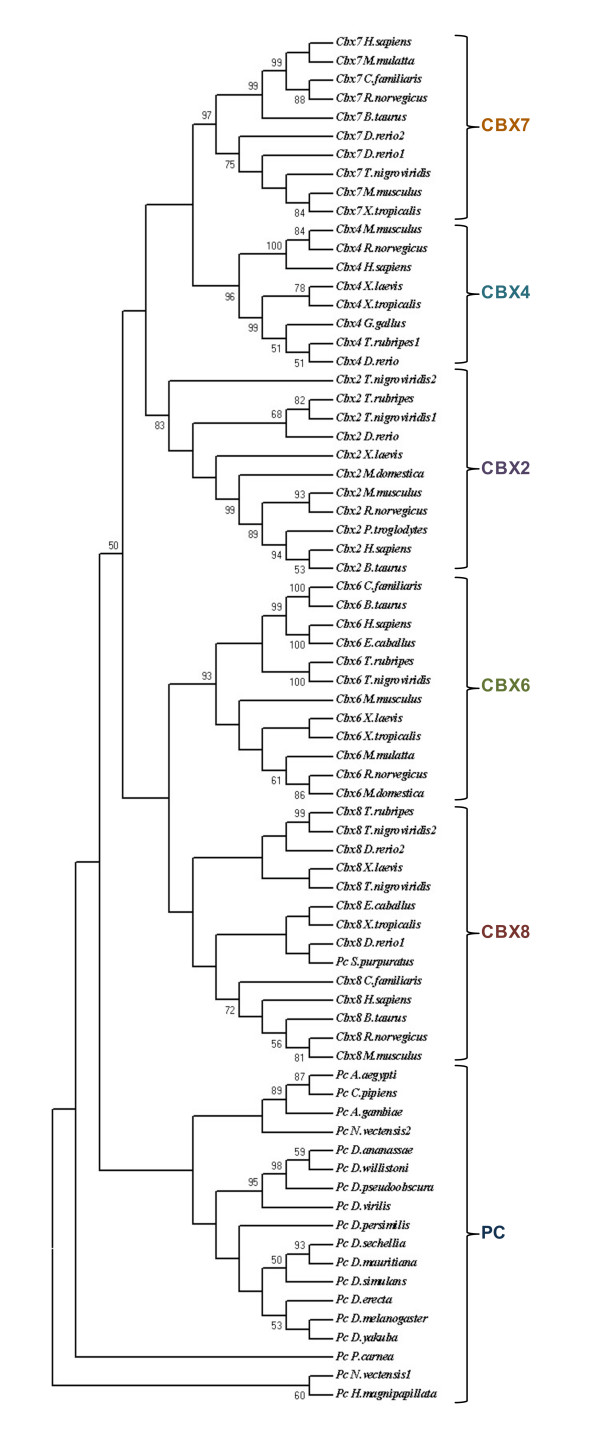
**The Phylogenetic tree of PC homologues**. The phylogenetic tree was constructed using neighbour joining method. The branching pattern for invertebrates PC and vertebrate PC homologues are highlighted in different colours. The bootstrap value (>50%) of 1000 replicates is shown in the branches. The sequences are named with the homologue name and species name.

The homologues identified by our analysis (Additional file 1) were subjected to phylogenetic analysis. The phylogenetic tree constructed by maximum parsimony method is in agreement with our grouping based on motif conservation (Figure 10). The phylogenetic analysis of PC homologues shows that insect PC homologues group into a single branch. In vertebrates, each CBX protein branches along with their other vertebrate counterparts (Figure 9). For example CBX2 of human shows more similarity with CBX2 of other vertebrates but not with the other paralogues present in human. This indicates that each CBX protein is evolving independently since the duplication event under a common selection pressure in different vertebrate lineages. The phylogenetic analysis of chromodomain and PcR box also show similar clustering (Figure 6 &9). While each CBX protein from different vertebrate species grouped together whether we use chromodomain, PcR domain or entire protein for phylogenetic tree analysis, the branching pattern varies depending upon the sequence used for the analysis (see Additional file 7). As the bootstrap values for the branching of each CBX protein cluster are not significant, it is difficult to speculate the closeness in terms of homology between different CBX proteins. This analysis also suggests that domains of PC homologues have independently evolved within certain constraints during expansion of vertebrate lineage.

The members of PRC2 are conserved across the species including plants [55]. Earlier studies, although suggested that members of PRC1 are absent in plants [56], recent findings indicate that plant do have distant homologues of these genes. *Arabidopsis *protein LHP1/TFL2 that has chromodomain and a chromoshadow domain, like HP1 in animals, does recognize H3K27Me3 modification which is a major repressive mark in plants [57-59]. LHP1/TFL2 is, therefore, considered as the functional homologue of PC in plants [58,60]. More recently, *Arabidopsis *homologues of ring finger proteins have been identified that also interact with LHP1 [61,62]. In our analysis we found PC homologues in cnidarians *Nematostella, Hydra *and *Podocoryne*. This shows that PC is pre bilaterian origin. The PC homologues of sea anemone show high sequence homology with the chromodomain and the PcR box of other PC homologues. PC of *Podocoryne carnea *interacts with mouse RING1B [63] which is a PRC1 member. This shows that PC appeared in the common ancestor of bilaterians and cnidarians, 570-700 millions years ago.

In nematode *Caenorhabditis elegans*, Cec-1 is predicted as a homologue of PC [64]. Functional analysis has shown that expression profile of cec-1 is similar to that of *Drosophila *Pc [65]. It has N-terminal chromodomain but the predicted PcR box of CEC-1 shows a poor sequence homology [64]. In *Drosophila *it has been shown that both N-terminal chromodomain and C-terminal PcR box is essential for PC function [40]. PcR box of vertebrates shows much higher sequence homology with PcR box of cnidarians compared to that of *C. elegans*. This raises the possibility that CEC-1 may be at best a highly diverged PC homologue. Function of PcR box includes the assembly of PRC1. In *C. elegans*, where all homologues of PRC2 members are present, only a few homologues of PRC1 members have been identified [61,66]. It remains possible that CEC-1 may not be a true PC homologue or it may be a remote homologue of PC that interacts with different class of proteins for its repressive function.

Invertebrates have one PC where as mammals have 5 homologues. The teleosts like Zebrafish, *Fugu *and *Tetraodon *have up to 7 PC homologues. This expansion of PC homologue in vertebrate shows remarkable parallel with the expansion of Hox gene complex. In teleosts PC genes are duplicated in species-specific manner. CBX8 is duplicated in Zebrafish and *Tetraodon*. CBX2, CBX4, CBX7 are duplicated in *Tetraodon*, *Fugu*, Zebrafish, respectively (see Additional file 1). Interestingly, teleosts have more copies of Hox clusters than mammals. Invertebrates have one Hox cluster where as tetrapods have four Hox clusters. In teleosts lineage, Hox cluster duplicated further and ray finned fishes have 7-8 Hox clusters. During vertebrate evolution, genome of the common ancestor of vertebrates underwent two rounds of duplication events that led to increase in the copy number of several genes [67] and many of the extra copies acquired novel functions. It is believed to have paved the way for increase in the system complexity. In teleosts lineage, genome underwent an additional round of species-specific duplication event that led to duplication of several genes.

## Discussion

PC is the key protein in the epigenetic control of gene expression from early development to adult stages. Precise expression of Hox genes along the anterior-posterior body axis and its maintenance in subsequent cell divisions is dependent on PcG and trxG of genes. PC homologues begin to appear in cnidarians. This early origin of PC and its expansion in vertebrates shows a remarkable parallel with the pre bilaterian-cnidarian origin of Hox complexes and their expansion in vertebrates [68]. While most vertebrates have five PC homologues, we found more PC homologues in fishes. This may be due to the fish specific genome duplications. Interestingly, fishes have more Hox clusters too [69,70]. Genome duplication event that occurred in vertebrate lineage [67] caused gene duplications that lead to increase in complexity-associated acquisition of novel functions in these additional copies. We show that different PC homologues carry distinct motifs and, therefore, are likely to have functional distinctions. This also suggests that common ancestor of vertebrate - the link between invertebrate and vertebrates - acquired novel features in various duplicated copies of the PC gene. These novelties have been conserved for more than 500 million years indicating their functional relevance in vertebrate diversity. Expansion of PC gene itself and that of several other members of PcG/trxG genes vis-à-vis expansion of genome size, in particular, the increasing proportion of non-coding part of genome indicates enforcement of epigenetic regulatory toolkit and its important role in vertebrate evolution. It is known that vertebrates that have four or more Hox clusters also have similarly higher number of PC homologues [69,70]. This parallel expansion may indicate that the expanded group of the master control Hox genes also need a matching degree of expansion of the epigenetic tools including PC. This may have been one of the selection pressures behind the amplification and conservation of PC genes in vertebrates.

The vertebrate homologues of *Polycomb *gene, CBX2, 4, 6, 7 and 8 acquired distinct combination of motifs that separates them from one another and have been remarkably well conserved. We also found that each vertebrate PC homologue has a putative DNA binding domain AT-Hook or ATHL, a newly identified variant. Since these motifs are present adjacent to the chromodomain that recognizes specific histone tail modification, it raises the possibility that PC contact with the target site may involve bimodal recognition and binding. In addition to chromodomain binding to the histone tail, AT-Hook/ATHL can bind to the adjacent site in the minor groove of DNA, sandwiching the DNA between PC and histone surface (see Additional file 8). It is interesting to note that this interaction will 'lock' the nucleosome from shuffling or remodelling and may explain why PRC1 makes nucleosome more stable and the entire region repressive for transcription [1]. While several DNA binding proteins are known as the members of the PcG in flies, our finding that all CBX counterparts in vertebrates contain their own DNA binding motif, indicates that at least at some loci in vertebrate genomes PRC1 by itself can read the epigenetic histone tail mark and associate with it.

Why vertebrates have more PC homologues? In this study we report for the first time that each homologue has its own signature. We think that the expansion, which allowed addition of novel features to different PC homologues, created greater possibilities in the variety of factors interacting with this protein. This may also allow distinct kind of PRC1 complexes depending on the CBX homologue associated with it. Very recently CBX7 and CBX8 have been shown to exist in distinct complexes [71], it remains to be seen, however, if other homologues follow the same trend. In mouse embryonic stem cells, PC homologues follow distinct sub nuclear localization patterns and show differential dynamics during ES cell differentiation [72,73]. It is possible that novel motifs identified in our study may contribute to these distinct properties of the PC homologues by themselves or with the help of different sets of interactors. It also remains possible that some CBX proteins may function as individuals and not as components of a complex. Detailed biochemical analysis will be needed to explore these possibilities.

We also identified a novel insect specific conserved motif, CIPC, absent in vertebrate homologues. While the function of this novel domain remains to be studied, it seems likely that expansion of *Polycomb *gene and acquisition of novel motifs have come at the expense of the CIPC. Expansion of epigenetic mechanisms has been one of the significant events in the evolution of complexity. Since PcG and trxG are the key players in regulating the differential expression of genes, having more homologues of these groups of proteins may have provided regulatory capabilities needed for the evolution of complex organisms. Diversification of PC function (recognition of histone tail modification by its chromodomain and recruitment of repressor factor by PcR box) coincides with the trade off between insect specific CIPC and vertebrate homologue specific motifs including the DNA binding motif AT-Hook/ATHL. We suggest that CIPC may be restricting PC to selected loci or processes and that its absence allows the new protein architecture to be recruited for diverse functions depending on the newly acquired motifs. It will be important to dissect out the function of these new motifs to understand the epigenetic mechanisms of developmental gene regulation controlled by PC homologues and various disease conditions where these proteins are involved.

## Conclusion

Using genome wide scanning approach we report an efficient strategy to mine homologues in the genome databases. We find that PC is of pre-bilaterian origin and it is evolving from the common ancestor of bilaterians and cnidarians. We find that multiple PC homologues present in vertebrates, CBX2, 4, 6, 7 & 8, have acquired CBX protein specific conserved motifs, including signature motifs for each homologue. This indicates that these homologues emerged early during the vertebrate evolution and have been conserved under positive selection pressure. The chromodomain and PcR domains of vertebrate PC proteins also show orthologue specific features indicating functional uniqueness of these domains as well within the globally conserved constraints. We also see two major features in the PC that sets apart the vertebrate and insect proteins - presence of CIPC motif in the insect protein and that of DNA binding AT-Hook or ATHL (identified in this study) motif in all vertebrate homologues. We also suggest a model in which chromdomain-H3K27Me3 interaction on the one hand and AT-Hook/ATHL - DNA interaction on the other, locks nucleosomes in repressed state.

Several lines of studies indicate that complexity of the higher vertebrates evolved by increase in the non-coding part of the genome that is likely to have functional elements where a set of trans-acting factors interact. One of such set of factors is the PcG system. Our study shows a quick expansion and acquisition of novelties in vertebrate PC homologues. We suggest that increase in the number of homologues of these proteins resulted into the expansion of epigenetic mechanisms in the common ancestor of vertebrates that led to the evolution of complexity and diversity in these organisms.

## Methods

### Mining of the PC homologues

One PC is present in fly and other insects. The *Drosophila *PC protein was used as a seed for mining its homologues. The overall schema for mining PC homologues is shown in Figure 1. PC homologues were searched in NCBI Nr Genpept (release156, 3,570,920 sequences) database using PSI BLAST [74]. It is based on iterative profile based search and constructs position specific weight matrices on the basis of the alignment generated by the blast search. This matrix was used to score the next iteration. The availability of protein sequence information is lesser than the possible coding regions in genome. So it is essential to search in genome to find the homologues, which are not reported in protein databases. For this purpose the genome sequence of model organisms (see Additional file 9) were also searched by using tBLASTN [75]. The genomic region showing hits with any length irrespective of their score and identity were considered as probable candidates for our analysis. The BLAST hits were parsed and the regions showing hits were extended 5 kb upstream and 5 kb downstream. After extending their length if any hit over laps with another hit, they were combined together and extended 5 kb further from the overlapping regions. This approach will be useful to extract larger size gene sequence and homologues with less sequence homology. The extended blast hits were subjected for gene prediction. Genscan [76] was used to predict genes in the genomic regions showing hits with PC of *Drosophila*. The sequences showing similarity >98% over the length of the sequences in same organism were considered as redundant set. The predicted genes of same organism were aligned using Blastclust [75] and clustered with above cut off and redundant data were removed.

### Motif prediction

The sequence homology among PC homologues is less. Conventional methods to find sequence homologues based on sequence homology are not efficient enough to pick up conserved regions in sequences having less sequence homology in larger dataset. The conserved motifs among our training dataset were predicted and used for picking true homologues. The motifs were predicted by using the program MEME [77]. It uses expectation maximization algorithm to identify motifs with best width, in a group of protein or DNA sequences. The predicted motifs were assigned to Genpept hits and predicted proteins of our genome wide search approach. The sequences were aligned based on motif conservation by MAST (Motif Alignment Search Tool) [78]. The conserved regions in homologues were extracted from predicted motifs and HMM profile was created by using HMMER [79]. The profiles were searched in Swissprot protein sequence database to identify the existence of these conserved regions in other proteins.

### Multiple sequence alignment and phylogenetic analysis

The multiple sequence alignment was done by using ClustalW [80] and T-Coffee [81]. The alignments were visualized by using ClustalX [82]. The consensus pattern was drawn in logo format using WebLogo [83]. The phylogenetic analysis was performed by maximum parsimony method using MEGA [84]. The sequences were bootstrapped 1000 times, to check the chance for occurrence of each internal branch of the tree. This method is helpful to know the reliability of tree.

### Secondary structure analysis

The secondary structure prediction was done by using PSIPRED [85]. It uses a neural network method to predicts the secondary structure based on PSI BLAST result of the given query sequence.

## Authors' contributions

RKM suggested the analysis and designed the strategy. RS performed the analysis. RS and RKM analyzed the data and wrote the manuscript. Both authors have read and approved the final manuscript.

## Supplementary Material

Additional file 1 **The Polycomb homologues**. PC homologues available in the NCBI protein sequence database and the homologues predicted by our genome wide search approach are given in the table. Click here for file

Additional file 2 **Secondary structure of the homologues**. The predicted secondary structures of human and fly PC homologues are shown and conserved regions are highlighted. Click here for file

Additional file 3 **Motifs predicted in PC homologues**. The motifs predicted in PC homologues are represented in BLOCKS format. Sequences are named with the protein name followed by species name. Click here for file

Additional file 4 **The motif alignment of PC homologues**. The motifs predicted by MEME tool are aligned to the homologues. Click here for file

Additional file 5 **Conserved regions of PC homologues**. The conserved motifs that are described in Figures 2 and 3 are represented in multiple sequence alignment format. Click here for file

Additional file 6 **List of AT Hook like motif containing proteins**. The table describes proteins having AT Hook Like motif, ATHL, from different organisms. Click here for file

Additional file 7 **The phylogenetic tree of Chromdomain, PcR box and PC homologues**. The phylogenetic tree generated using Chromodomain, PcR box and PC homologues (entire protein) represented in Figures 6, 9 and 10 are shown in a simplified form based on the consensus branching of the homologues. Click here for file

Additional file 8 **A nucleosome cartoon showing three way interaction of DNA-polycomb-H3K27Me3**. AT-Hook/ATHL motif is shown to interact with DNA while adjacent chomodomain interacts with the histone H3K27Me3. This three-way interaction locks the nucleosome disallowing any remodelling or dissociation till PC (alone or as part of PRC1) is dislodged. Click here for file

Additional file 9 **List of genome databases searched for mining the homologues**. List shows name of the organism and genome database source used in this study to look for PC homologues. Click here for file
