# Supplementary material for: Novel motifs distinguish multiple homologues of Polycomb in vertebrates: expansion and diversification of the epigenetic toolkit
Source: BMC Genomics. 2009 Nov 20;10:549. doi: 10.1186/1471-2164-10-549 (PMC2784810; doi:10.1186/1471-2164-10-549)
Supplement: Additional file 1 — The Polycomb homologues. PC homologues available in the NCBI protein sequence database and the homologues predicted by our genome wide search approach are given in the table. [file 1471-2164-10-549-S1.PDF]

Additional file 1 - The *Polycomb* homologues

Organism	Polycomb homologues				
**Vertebrates**	**Cbx2 (Pc1, M33)**	**Cbx4 (Pc2)**	**Cbx6**	**Cbx7**	**Cbx8 (Pc3)**
Homo sapiens (Hs)	Q14781	NP_003646.2	AAH12111.1	NP_783640.1	NP_065700.1
Mus musculus (Mm)	NP_031649(M33)	O55187	BAC29001.1	EDL04618	NP_038954.1
Rattus norvegicus (Rn)	EDM06770.1	XP_576815.2	EDM15778.1	EDM15772	AAH99206.1
Pan troglodytes (Pt)	NW_118553.1[*]	-	NW_001230981.1[*+]	NW_121190.1[*]	XP_523736.2[+]
Canis familiaris (Cf)	XP_540468.2[+]	XP_850702.1[+]	XP_538370.2	XP_538368.2	XP 850749.1
Bos Taurus (Bt)	XP_583104.3	XP_869814.2[+]	AAI33331.1	XP_604126	NP_001071330.1
Macaca mulatta (Mmu)	XP_001108961[+]	XP_001109437.1[+]	XP_001096528.1	XP_001097286.1	XP_001109073.1[+]
Monodelphis domestica (Md)	XP_001370995.1	NW_001581875.1[*]	NW_001582016.1[*]	XP_001367417.1 [+]	-
Equus caballus (Ec)	XP_001490541.1[+]	-	NW_001799702.1[*]	XP_001499977.1[+]	XP_001490422.1
Ornithorhynchus anatinus (Oa)	XP_001517212.1[+]	NW_001635282.1[+]	-	NW_001626409.1[*+]	-
Gallus gallus (Gg)	XP_423911.2[+]	NP_989973.1(CHCB3)	XP_425476.2[+]	NW_060209.1[*+]	XP_001231757.1[+]
Xenopus laevis (Xl)	AAH72868.1	AAC59728(X Polycomb)	AAH82876.1	-	AAH87529.1
Xenopus tropicalis (Xt)	scaffold_1094[*]	NP_001096327.1	NP_001016617.1	NP_001005071.1	NP_001072443.1
Tetraodon nigroviridis (Tn)	CAG02234.1(Cbx2a)<br>Chr2(Cbx2b)[*]	CAG11435.1[+]	CAG12544	CAG04517.1	CAF92674.1 (Cbx8a)<br>CAG11436.1 (Cbx8b)
Danio rerio (Dr)	NP_919354.1	NP_991312.1	XP_684076.2[+]	XP_687769.1 (Cbx7a)<br>NP_001017853.1(Cbx7b)	AAH95631.1 (Cbx8a)<br>AAN87353.1 (Cbx8b)
Takifugu rubripes (Fr)	22418806[*]	22418301[*] (Cbx4a)<br>22421923(Cbx4b)[+]	CAAB01001373.1[*]	Scaffold3[+]	22418301[*]
**Invertebrates**	**Pc**				
Aedes aegypti (Aa)	EAT33624.1				
Anopheles gambiae (Ag)	19612143[*]				
Apis mellifera (Am)	XP_001121316.1				
Bombyx mori (Bm)	AADK01013325.1[*]				

Culex pipiens quinquefasciatus (Cp)	EDS31965.1
Drosophila ananassae (Da)	AAPP01019312.1*
Drosophila erecta (De)	AAPQ01006587.1*
Drosophila mauritiana (Dma)	ABY55463.1
Drosophila melanogaster (Dm)	AAL49241.1
Drosophila persimilis (Dpe)	76465459*
Drosophila pseudoobscura (Dps)	EAL29819.1
Drosophila sechellia (Dse)	76493180*
Drosophila simulans (Dsi)	111499470*
Drosophila virilis (Dv)	AANI01017370.1*
Drosophila willistoni (Dw)	AAQB01007738.1*
Drosophila yakuba (Dy)	84678090*
Nasonia vitripennis (Nvi)	XP_001600331.1+
Nematostella vectansis (Nve)	XP_001640371.1, XP_001632346.1
Pediculus humanus corporis (Ph)	AAZO01000027.1*+
Podocoryne carnea (Pc)	AAM28241.1
Tribolium castaneum (Tc)	XP_974290
Hydra magnipapillata (Hm)	CL1767Contig1*
Strongylocentrotus purpuratus (Sp)	Scaffold_v2_34702*

PC homologues available in the NCBI protein database and the homologues predicted by our genome wide search approach are listed. The sequences are represented with their NCBI accession number. The sequences that are not reported in the NCBI protein database and predicted by our genome wide search are represented with their corresponding contig number and highlighted with *. + denotes the partial sequence and these were not used in further analysis.
