# Supplementary material for: Novel motifs distinguish multiple homologues of Polycomb in vertebrates: expansion and diversification of the epigenetic toolkit
Source: BMC Genomics. 2009 Nov 20;10:549. doi: 10.1186/1471-2164-10-549 (PMC2784810; doi:10.1186/1471-2164-10-549)
Supplement: Additional file 2 — Secondary structure of the homologues. The predicted secondary structures of human and fly PC homologues are shown and conserved regions are highlighted. [file 1471-2164-10-549-S2.PDF]

Additional file 2 - Secondary structure of the homologues

The predicted secondary structures in human and fly homologues are shown. The amino acid sequence, predicted secondary structure for each amino acid and the confidence score for the prediction is represented. The secondary structure for the conserved regions are highlighted.

➢ CBX2(*Homo sapiens*)

```
Conf: 987555586117866773235778866999997588841122263665057799999999
Pred: CCCCCCCCCCEEEEEEEEEEEEEECCEEEEEEEEECCCCCCCCCCCHHHHCCHHHHHHHHH
  AA: MEELSSVGEQVFAAECILSKRLRKGKLEYLVKWRGWSSKHNSWEPEENILDPRLLLAFQK
              10        20        30        40        50        60

Conf: 999876302444556776654433577766444466776532466766767211354222
Pred: HHHHHHHHCCCCCCCCCCCCCCCCCCCCCCCCCCCCCCCCCCCCCCCCCCCCCCCCCCCC
  AA: KEHEKEVQNRKRGKRPRGRPRKLTAMSSCSRRSKLKEPDAPSKSKSSSSSSSSTSSSSSS
              70        80        90       100       110       120

Conf: 456777886667887774577886656542566656777634578757786777331125
Pred: CCCCCCCCCCCCCCCCCCCCCCCCCCCCCCCCCCCCCCCCCCCCCCCCCCCCCCCCCCCC
  AA: DEEDDSDLDAKRGPRGRETHPVPQKKAQILVAKPELKDPIRKKRGRKPLPPEQKATRRPV
             130       140       150       160       170       180

Conf: 643245665447778866567776654467545445776467788750061100001355
Pred: CCCCCCCCCCCCCCCCCCCCCCCCCCCCCCCCCCCCCCCCCCCCCCCCCCCCCCCCCCCC
  AA: SLAKVLKTARKDLGAPASKLPPPLSAPVAGLAALKAHAKEACGGPSAMATPENLASLMKG
             190       200       210       220       230       240

Conf: 545466476453575666523377666578765566543545567776346667776457
Pred: CCCCCCCCCCCCCCCCCCCCCCCCCCCCCCCCCCCCCCCCCCCCCCCCCCCCCCCCCCCC
  AA: MASSPGRGGISWQSSIVHYMNRMTQSQAQAASRLALKAQATNKCGLGLDLKVRTQKGELG
             250       260       270       280       290       300

Conf: 776664435675667666767887777765575766887888887777888750046777
Pred: CCCCCCCCCCCCCCCCCCCCCCCCCCCCCCCCCCCCCCCCCCCCCCCCCCCCCCCCCCCC
  AA: MSPPGSKIPKAPSGGAVEQKVGNTGGPPHTHGASRVPAGCPGPQPAPTQELSLQVLDLQS
             310       320       330       340       350       360

Conf: 757524764558888877744753436888888813015766578766665541012026
Pred: CCCCCCCCCCCCCCCCCCCCCCCCCCCCCCCCCCCCCCCCCCCCCCCCCCCCCCEEEECC
  AA: VKNGMPGVGLLARHATATKGVPATNPAPGKGTGSGLIGASGATMPTDTSKSEKLASRAVA
             370       380       390       400       410       420

Conf: 887874402456765676755778888788881011104687778888888877798863
Pred: CCCCCCCCCCCCCCCCCCCCCCCCCCCCCCCCHHCCCCCCCCCCCCCCCCCCCCCCCCCC
  AA: PPTPASKRDCVKGSATPSGQESRTAPGEARKAATLPEMSAGEESSSSDSDPDSASPPSTG
             430       440       450       460       470       480

Conf: 3744134578871011101783799656403146886403354541000259
Pred: CCCCCCCCCCCCCHHHHCCCCCEEEEEEEEECEEEEEEEEECCCCCCCCCCCCC
  AA: QNPSVSVQTSQDWKPTRSLIEHVFVTDVTANLITVTVKESPTSVGFFNLRHY
```

490 500 510 520 530

CBX4(*Homo sapiens*)

```
Conf: 987654660244333304121278679999964898345442676776998999999999
Pred: CCCCCCCCCEEEEEEEECEEEECCEEEEEEEECCCCHHHCCCCCHHHCCCHHHHHHHHHH
  AA: MELPAVGEHVFAVESIEKKRIRKGRVEYLVKWRGWSPKYNTWEPEENILDPRLLIAFQNR
              10        20        30        40        50        60

Conf: 865531011236777764110157887654444455656433454033035554510245
Pred: HHHHHHHCCCCCCCCCCCCCCCCCCCCCCCCCCCCCCCCCCCCCCEEECCCCCCCCHHHH
  AA: ERQEQLMGYRKRGPKPKPLVVQVPTFARRSNVLTGLQDSSTDNRAKLDLGAQGKGQGHQY
              70        80        90       100       110       120

Conf: 664888716675666646777788888876088377553774577640222545786434
Pred: HHHHHHHHCCCCCCCCCCCCCCCCCCCCCCEEECCCCCCCCCCCCCCEEECCCCCCCCCC
  AA: ELNSKKHHQYQPHSKERAGKPPPPGKSGKYYYQLNSKKHHPYQPDPKMYDLQYQGGHKEA
             130       140       150       160       170       180

Conf: 786775424478884111247322575166441011035876432236876788870210
Pred: CCCCCCCCCCCCCCCCCCCCCCCCCCCCCCCCCHHHCCCCCCCCCCCCCCCCCCCCHHHC
  AA: PSPTCPDLGAKSHPPDKWAQGAGAKGYLGAVKPLAGAAGAPGKGSEKGPPNGMMPAPKEA
             190       200       210       220       230       240

Conf: 566676633588750587871999972420148027875055422354377555333332
Pred: CCCCCCCCEEEEEECCCCCCCEEEEEHHHCCCCCEEEEEECCCCCCCCCCCCCCCCCCCC
  AA: VTGNGIGGKMKIVKNKNKNGRIVIVMSKYMENGMQAVKIKSGEVAEGEARSPSHKKRAAD
             250       260       270       280       290       300

Conf: 358733437120020121220157000101001356666311266666755566465740
Pred: CCCCCCCCCHHHCCCHHHCCCCCCCHHHHHHHCCCCCCCCCCCCCCCCCCCCCCCCCCCC
  AA: ERHPPADRTFKKAAGAEEKKVEAPPKRREEEVSGVSDPQPQDAGSRKLSPTKEAFGEQPL
             310       320       330       340       350       360

Conf: 001575545568422467755576777655664356654334541231556630265556
Pred: EECCCCCCCCCCCCCCCCCCCCCCCCCCCCCCCCCCCCCCCCCCCCCCHHHHHCCCCCCC
  AA: QLTTKPDLLAWDPARNTHPPSHHPHPHPHHHHHHHHHHHAVGLNLSHVRKRCLSETHGE
             370       380       390       400       410       420

Conf: 110000000001267644678887657666871002579847832588777701022123
Pred: CCCHHHEECCCCCCCCCCCCCCCCCCCCCCCCCCCCCCCCCCCEEEECCCCCCCCCCEEECC
  AA: REPCKKRLTARSISTPTCLGGSPAAERPADLPPAAALPQPEVILLDSDLDEPIDLRCVKT
             430       440       450       460       470       480

Conf: 234677764220025688776610045447877655675320000124543301200557
Pred: CCCCCCCCCCCCCCCCCCCCCCCCCCCCCCCCCCCCCCCCCCCHHCCCCCHHHCCCCCCE
  AA: RSEAGEPPSSLQVKPETPASAAVAVAAAAAPTTTAEKPPAEAQDEPAESLSEFKPFFGNI
             490       500       510       520       530       540

Conf: 98522057468875212309
Pred: EEEEEECCEEEEEEEEEEEC
  AA: IITDVTANCLTVTFKEYVTV
             550       560
```

➢ CBX6(*Homo sapiens*)

```
Conf: 987664863366344414021288689999965887355343766777968999999999
Pred: CCCCCCCCCEEEEEEEECEEEECCEEEEEEEECCCCHHHCCCCCHHHCCCHHHHHHHHHH
  AA: MELSAVGERVFAAESIIKRRIRKGRIEYLVKWKGWAIKYSTWEPEENILDSRLIAAFEQK
              10        20        30        40        50        60

Conf: 986542031014778764211145675433201222101457887742000000000145
Pred: HHHHHHHCCCCCCCCCCCCCCCCCCCCCCCCCHHHHHHCCCCCCCCCCCCCCHHCCCHHH
  AA: ERERELYGPKKRGPKPKTFLLKARAQAEALRISDVHFSVKPSASASSPKLHSSAAVHRLK
              70        80        90       100       110       120

Conf: 530100256657756677788878736787764102303545427887743333365565
Pred: HHCCCCCCCCCCCCCCCCCCCCCCCCCCCCCCCCCEEECCCCCCCCCCCCCCCCCCCCCC
  AA: KDIRRCHRMSRRPLPRPDPQGGSPGLRPPISPFSETVRIINRKVKPREPKRNRIILNLKV
             130       140       150       160       170       180

Conf: 436788981201777433545787556577654434777655476543212343147841
Pred: CCCCCCCCHHHCCCCCCCCCCCCCCCCCCCCCCCCCHHHHHHHHHHHHHHHHHHHCCCCC
  AA: IDKGAGGGGAGQGAGALARPKVPSRNRVIGKSKKFSESVLRTQIRHMKFGAFALYKPPPA
             190       200       210       220       230       240

Conf: 127788874310123621000257545576888899855665433472223202200000
Pred: CCCCCCCCCCCCCCCCCCCCCCCCCCCCCCCCCCCCCCCCCCCCCCCCCCCCCHHHCCC
  AA: PLVAPSPGKAEASAPGPGLLLAAPAAPYDARSSGSSGCPSPTPQSSDPDDTLPKLLPETV
             250       260       270       280       290       300

Conf: 777776575334666877332435452773102103654223577532112335666674
Pred: CCCCCCCCCCCCCCCCCCCCCCCCCCCCCCCHHHCCCCCCCCCCCCCCCCCCCCCCCCCC
  AA: SPSAPSWREPEVLDLSLPPESAATSKRAPPEVTAAAGPAPPTAPEPAGASSEPEAGDWRP
             310       320       330       340       350       360

Conf: 4134222467510266589998317872135566420001356766454559
Pred: CCCCCCEEEEEECCCCEEEEEEECCCCCCCHHHHHHHCCCCCCCCCCCCCCC
  AA: EMSPCSNVVVTDVTSNLLTVTIKEFCNPEDFEKVAAGVAGAAGGGGSIGASK
             370       380       390       400       410
```

- CBX7(*Homo sapiens*)

```
Conf: 987442641000001103232488789999975898466422556656848999999999
Pred: CCCCCCCCCHHHHEEECCEEEECCEEEEEEEEECCCCHHHCCCCCHHHCCCHHHHHHHHH
  AA: MELSAIGEQVFAVESIRKKRVRKGKVEYLVKWKGWPPKYSTWEPEEHILDPRLVMAYEEK
                10        20        30        40        50        60

Conf: 986210156778866301004677876656444611100034123201788870101444
Pred: HHHHHHCCCCCCCCCCCHHCCCCCCCCCCCCCCCCCCCCCCCCCHHHHCCCCCCHHHCCCC
  AA: EERDRASGYRKRGPKPKRLLLQRLYSMDLRSSHKAKGKEKLCFSLTCPLGSGSPEGVVKA
                70        80        90       100       110       120

Conf: 442213456777632344101001400135440124756664436521544114667776
Pred: CCCCCCCCCCCCCCCCCCCCHHHCCCCCCCCCCCCCCCCCCCCCCCCHHHHCCCCCCCCC
  AA: GAPELVDKGPLVPTLPFPLRKPRKAHKYLRLSRKKFPPRGPNLESHSHRRELFLQEPPAP
               130       140       150       160       170       180

Conf: 754665444887667541110586889885456766478566446623278884202222
Pred: CCCCCCCCCCCCCCCCCCHHHCCCCCCCCCCCCCCCCCCCEEEEEEECEEEEEEEECCCC
  AA: DVLQAAGEWEPAAQPPEEEADADLAEGPPPWTPALPSSEVTVTDITANSITVTFREAQAA
               190       200       210       220       230       240

Conf: 35000101689
Pred: CCCHHHCCCCC
  AA: EGFFRDRSGKF
               250
```

➢ CBX8(*Homo sapiens*)

```
Conf: 987654631023224305032588779999965898013033555877999999999999
Pred: CCCCCCCCCEEEEEEEEECCEEECCEEEEEEECCCCCHHCCCCCHHHCCCHHHHHHHHHH
  AA: MELSAVGERVFAAEALLKRRIRKGRMEYLVKWKGWSQKYSTWEPEENILDARLLAAFEER
                10        20        30        40        50        60

Conf: 998764055336777776432232211123454356654344545678774122677754
Pred: HHHHHHCCCCCCCCCCCCCCCCCCCCCCCCCCCCCCCCCCCCCCCCCCCCCCCCCCCCCC
  AA: EREMELYGPKKRGPKPKTFLLKAQAKAKAKTYEFRSDSARGIRIPYPGRSPQDLASTSRA
                70        80        90       100       110       120

Conf: 456678777788778776544555566447775465435677754022101321010054
Pred: CCCCCCCCCCCCCCCCCCCCCCCCCCCCCCCCCCCCCCCCCCCHHHHCCCCCHHHCCC
  AA: REGLRNMGLSPPASSTSTSSTCRAEAPRDRDRDRDRDRERDRERERERERERERERERER
               130       140       150       160       170       180

Conf: 410200246780103223563102110143457687543563100001486637888887
Pred: CCCEEECCCCCCCCCCCCCCHHHHHHCCCCCCCCCCCCCCCCCHHHCCCCCCCCCCCCC
  AA: GTSRVDDKPSSPGDSSKKRGPKPRKELPDPSQRPLGEPSAGLGEYLKGRKLDDTPSGAGK
               190       200       210       220       230       240

Conf: 888743355666545640167888889876314341011221002431001333125667
Pred: CCCCCCCCCCCCCCCCCCCCCCCCCCCCCCCCHHHHHCCCCCCCCCCCCHHHHHHCCCCC
  AA: FPAGHSVIQLARRQDSDLVQCGVTSPSSAEATGKLAVDTFPARVIKHRAAFLEAKGQGAL
               250       260       270       280       290       300

Conf: 886200112556300100000158767887310111112310378653466756344323
Pred: CCCCCEEECCCCCHHHHHHHCCCCCCCCCCCCHHHHHHHHHCCCCCCCCCCCCCCCCCCE
  AA: DPNGTRVRHGSGPPSSGGGLYRDMGAQGGRPSLIARIPVARILGDPEEESWSPSLTNLEK
               310       320       330       340       350       360

Conf: 58851025748999840688312011059
Pred: EEEEECCCCEEEEEEECCCCCCCHHHCCC
  AA: VVVTDVTSNFLTVTIKESNTDQGFFKEKR
               370       380
```

➢ POLYCOMB (*Drosophila melanogaster*)

```
# PSIPRED HFORMAT (PSIPRED V2.5 by David Jones)

Conf: 977776751000466678886434444221305210088317999850788114245733
Pred: CCCCCCCCCHHCCCCCCCCCCCCCEEEEEEECCCEEECCEEEEEEEECCCCHHHCCCCCH
  AA: MTGRGKGSKGKLGRDNATDDPVDLVYAAEKIIQKRVKKGVVEYRVKWKGWNQRYNTWEPE
              10        20        30        40        50        60

Conf: 547999999999998643321001477767777776653223444335577431100356
Pred: HHCCCHHHHHHHHHHHHHHHHCCCCCCCCCCCCCCCCCCCCCCCCCCCCCCCCCCEECCC
  AA: VNILDRRLIDIYEQTNKSSGTPSKRGIKKKEKEPDPEPESEEDEYTFTENDVDTHQATTS
              70        80        90       100       110       120

Conf: 643233466543365334533467543557776434566655543168887124121378
Pred: CCCCCCCCCCCCCCCCCCCCCCCCCCCCCCCCCCCCCCCCCCCCCCCCCCCCEEEEECCC
  AA: SATHDKESKKEKKHHHHHHHHHHHIKSERNSGRRSESPLTHHHHHHHESKRQRIDHSSSS
             130       140       150       160       170       180

Conf: 877777436778767766542235565432444655764423256888452125557533
Pred: CCCCCCCCCCCCCCCCCCCCCCCCCCCCCCCCCCCCCCCCCCCCCCCCCCCCCCCCCCCC
  AA: NSSFTHNSFVPEPDSNSSSSEDQPLIGTKRKAEVLKESGKIGVTIKTSPDGPTIKPQPTQ
             190       200       210       220       230       240

Conf: 125542066645676544544233577764443101257776376655542010475100
Pred: CCCCCCCCCCCCCCCCCCCCCCCCCCCCCCCCCCCHHHHHHHCCCCCCCCCCEECCCCCC
  AA: QVTPSQQQPFQDQQQAEKIASEAATQLKSEQQATPLATEAINTTPAESGAEEEEVANEEG
             250       260       270       280       290       300

Conf: 113556786357898876100567544436652225874000300145678479987632
Pred: CCCCCCCCCCCCCCCCCCCHHCCCCCCCCCCCCCCCCCCCCCCCCCCCCCCCEEEEEEEC
  AA: NQQAPQVPSENNNIPKPCNNLAINQKQPLTPLSPRALPPRFWLPAKCNISNRVVITDVTV
             310       320       330       340       350       360

Conf: 525786331122223210001466656779
Pred: CEEEEEEEECCCCCCCCCCCCCCCCCCCCC
  AA: NLETVTIRECKTERGFFRERDMKGDSSPVA
             370       380       390
```
