# Supplementary material for: Novel motifs distinguish multiple homologues of Polycomb in vertebrates: expansion and diversification of the epigenetic toolkit
Source: BMC Genomics. 2009 Nov 20;10:549. doi: 10.1186/1471-2164-10-549 (PMC2784810; doi:10.1186/1471-2164-10-549)
Supplement: Additional file 3 — Motifs predicted in PC homologues. The motifs predicted in PC homologues are represented in BLOCKS format. Sequences are named with the protein name followed by species name. [file 1471-2164-10-549-S3.PDF]

Additional file 3 - The Motifs predicted in the PC homologues

The motifs predicted in the homologues are represented in BLOCKS format. Sequences are named with the protein name followed by species abbreviation (for species name see Additional file 1).

```
Motif  1 width=29 seqs=70
Cbx8_Xt                  (   22) RKGRMEYLVKWKGWSQKYSTWEPEENILD  1
Cbx8_Ec                  (   22) RKGRMEYLVKWKGWSQKYSTWEPEENILD  1
Cbx8_Xl                  (   22) RKGRMEYLVKWKGWSQKYSTWEPEENILD  1
Cbx8_Bt                  (   22) RKGRMEYLVKWKGWSQKYSTWEPEENILD  1
Cbx8_Mm                  (   22) RKGRMEYLVKWKGWSQKYSTWEPEENILD  1
Cbx8_Rn                  (   22) RKGRMEYLVKWKGWSQKYSTWEPEENILD  1
Cbx8_Cf                  (   22) RKGRMEYLVKWKGWSQKYSTWEPEENILD  1
Cbx8_Hs                  (   22) RKGRMEYLVKWKGWSQKYSTWEPEENILD  1
Cbx4_Dr                  (   22) RKGRMEYLVKWRGWSPKYNTWEPEENILD  1
Cbx4_Gg                  (   22) RKGRVEYLVKWRGWSPKYNTWEPEENILD  1
Cbx4_Rn                  (   22) RKGRVEYLVKWRGWSPKYNTWEPEENILD  1
Cbx4_Mm                  (   22) RKGRVEYLVKWRGWSPKYNTWEPEENILD  1
Cbx4_Hs                  (   22) RKGRVEYLVKWRGWSPKYNTWEPEENILD  1
Cbx4_Tn                  (   22) RKGKVEYLVKWRGWSPKYNTWEPEENILD  1
Cbx4_Fr1                 (   22) RKGKVEYLVKWRGWSPKYNTWEPEENILD  1
Cbx4_Xt                  (   22) RKGRVEYLVKWRGWSSKYNTWEPEENILD  1
Cbx4_Xl                  (   22) RKGRVEYLVKWRGWSSKYNTWEPEENILD  1
Cbx6_Dr                  (   22) RKGRMEYLVKWKGWAIKYSTWEPEENILD  1
Cbx8_Tn                  (   22) RKGRLEYLVKWKGWSPKYSTWEPEENILD  1
Cbx6_Mmu                 (   22) RKGRIEYLVKWKGWAIKYSTWEPEENILD  1
Cbx8_Dr2                 (   22) RRGRMEYLVKWKGWSQKYSTWEPEENILD  1
Cbx6_Xt                  (   22) RKGRIEYLVKWKGWAIKYSTWEPEENILD  1
Cbx6_Xl                  (   22) RKGRIEYLVKWKGWAIKYSTWEPEENILD  1
Cbx6_Hs                  (   22) RKGRIEYLVKWKGWAIKYSTWEPEENILD  1
Cbx6_Bt                  (   22) RKGRIEYLVKWKGWAIKYSTWEPEENILD  1
Cbx6_Mm                  (   22) RKGRIEYLVKWKGWAIKYSTWEPEENILD  1
Cbx6_Rn                  (   22) RKGRIEYLVKWKGWAIKYSTWEPEENILD  1
Cbx6_Cf                  (   22) RKGRIEYLVKWKGWAIKYSTWEPEENILD  1
Cbx8_Tn2                 (   22) RRGRWEYLVKWKGWSQKYNTWEPEENILD  1
Cbx8_Fr                  (   22) RRGRWEYLVKWKGWSQKYNTWEPEENILD  1
Cbx7_Md                  (   22) RKGKVEYLVKWKGWPPKYSTWEPEEHILD  1
Cbx7_Mmu                 (   22) RKGKVEYLVKWKGWPPKYSTWEPEEHILD  1
Cbx7_Ec                  (  122) RKGKVEYLVKWKGWPPKYSTWEPEEHILD  1
Cbx7_Mm                  (   22) RKGKVEYLVKWKGWPPKYSTWEPEEHILD  1
Cbx7_Rn                  (   22) RKGKVEYLVKWKGWPPKYSTWEPEEHILD  1
```

```
Cbx7_Bt                 (   22) RKGKVEYLVKWKGWPPKYSTWEPEEHILD  1
Cbx7_Xt                 (   22) RKGKVEYLVKWKGWPPKYSTWEPEEHILD  1
Cbx7_Cf                 (   22) RKGKVEYLVKWKGWPPKYSTWEPEEHILD  1
Cbx7_Hs1                (   22) RKGKVEYLVKWKGWPPKYSTWEPEEHILD  1
Cbx8_Dr1                (   22) RRGHMEYLVKWKGWSPKYSTWEPEENILD  1
Pc_Tc                   (   19) RRGVVEYYVKWKGWSQKHNTWEPEENILD  1
Cbx2_Md                 (   23) RKGKLEYLVKWRGWSSKHNSWEPEENILD  1
Cbx2_Pt                 (   23) RKGKLEYLVKWRGWSSKHNSWEPEENILD  1
Cbx2_Rn                 (   23) RKGKLEYLVKWRGWSSKHNSWEPEENILD  1
Cbx2_Mm                 (   23) RKGKLEYLVKWRGWSSKHNSWEPEENILD  1
Cbx2_Hs                 (   23) RKGKLEYLVKWRGWSSKHNSWEPEENILD  1
Cbx6_Tn                 (   22) RKGQLEYLVKWKGWAMKHSTWEPEENILD  1
Cbx6_Fr                 (   22) RKGQLEYLVKWKGWAMKHSTWEPEENILD  1
Pc_Am                   (   57) FKGKVEYFVKWKGWSKKYNTWEPEENILD  1
Cbx2_Tn2                (   21) RKGKFEYLVKWRGWSSKHNSWEPEENILD  1
Pc_Dps                  (   38) RKGTVEYRVKWKGWNQRYNTWEPEVNILD  1
Pc_Dpe                  (   38) RKGTVEYRVKWKGWNQRYNTWEPEVNILD  1
Cbx7_Dr2                (   22) RKGHVEYLLKWKGWPPKYSTWEPEEHILD  1
Cbx7_Tn                 (   22) RKGNVEYLLKWKGWPPKYSTWEPEEHILD  1
Pc_Nvi                  (  152) KRGKVEYYVKWKGWSKKYNTWEPEENILD  1
Pc_Nve1                 (   36) RKGQIEYLVKWRGWSAKYNTWEPAENILD  1
Pc_Ag                   (   20) RAGKVEYLVKWKGWSTRHNTWEPEENILD  1
Cbx2_Xl                 (   23) RKGTAEYLVKWRGWSSKHNSWEPEENILD  1
Pc_Dy                   (   37) KKGVVEYRVKWKGWNQRYNTWEPEVNILD  1
Pc_Dsi                  (   37) KKGVVEYRVKWKGWNQRYNTWEPEVNILD  1
Pc_Dm                   (   37) KKGVVEYRVKWKGWNQRYNTWEPEVNILD  1
Pc_Dse                  (   37) KKGVVEYRVKWKGWNQRYNTWEPEVNILD  1
Cbx7_Dr1                (   22) RKGNVEYLLKWQGWSPKYSTWEPEDNILD  1
Pc_Aa                   (   80) HAGKVEYLVKWKGWSTRHNTWEPEENILD  1
Cbx2_Tn1                (   23) RKGKLEFLVKWRGWSAKHNSWEPQENILD  1
Cbx2_Fr                 (   23) RKGKLEFLVKWRGWSAKHNSWEPQENILD  1
Cbx2_Dr                 (   23) RKGKLEYLVKWRGWSSKHNSWEPQENLLD  1
Pc_Pc                   (   23) KKGRAEYLVKWQGYSSKFNTWEPVENILD  1
Pc_Nve2                 (   22) RDGKVWYFIKWKGYSQRYNTWEPEENVLD  1
Cbx7_Pt                 (   22) RKGKVEYLVKWKGWPPKEERDRASGYRKR  1
//
```

```
Motif  2 width=29 seqs=66
Cbx2_Md                  (  495) IEHVFVTDVTANLITVTVKESPTSVGFFN  1
Cbx2_Mmu                 (  521) IEHVFVTDVTANLITVTVKESPTSVGFFN  1
Cbx2_Gg                  (  452) IEHVFVTDVTANLITVTVKESPTSVGFFN  1
Cbx2_Bt                  (  499) IEHVFVTDVTANLITVTVKESPTSVGFFN  1
Cbx2_Cf                  (  462) IEHVFVTDVTANLITVTVKESPTSVGFFN  1
Cbx2_Pt                  (  500) IEHVFVTDVTANLITVTVKESPTSVGFFN  1
Cbx2_Rn                  (  491) IEHVFVTDVTANLITVTVKESPTSVGFFN  1
Cbx2_Mm                  (  487) IEHVFVTDVTANLITVTVKESPTSVGFFN  1
Cbx2_Hs                  (  500) IEHVFVTDVTANLITVTVKESPTSVGFFN  1
Cbx2_Xl                  (  439) LEHVFVTDVTANLITVTVKESPTSVGFFN  1
Cbx8_Ec                  (  356) LEKVVVTDVTSNFLTVTIKESNTDQGFFK  1
Cbx8_Bt                  (  346) LEKVVVTDVTSNFLTVTIKESNTDQGFFK  1
Cbx8_Mm                  (  331) LEKVVVTDVTSNFLTVTIKESNTDQGFFK  1
Cbx8_Rn                  (  335) LEKVVVTDVTSNFLTVTIKESNTDQGFFK  1
Cbx8_Cf                  (  348) LEKVVVTDVTSNFLTVTIKESNTDQGFFK  1
Cbx8_Hs                  (  358) LEKVVVTDVTSNFLTVTIKESNTDQGFFK  1
Cbx8_Xt                  (  331) FEKVVVTDVTSNFLTVTIKESNTDQGFFK  1
Pc_Nve1                  (  420) IDQIVITDVTANFVTVTVKECCTDKGFFR  1
Cbx2_Tn1                 (  265) IEHVFVTDVTANLVTVTVKESPTSVGFFS  1
Cbx2_Fr                  (  455) IEHVFVTDVTANLVTVTVKESPTSVGFFS  1
Cbx2_Dr                  (  478) IEHVFVTDVTANLVTVTVKESPTSVGFFS  1
Cbx2_Tn2                 (  475) LEHVFVTDVTANFLTVTVKESPTSVGFFS  1
Cbx6_Xt                  (  328) CSNVVVTDVTSNLLTVTIKEFCNAQDFEK  1
Cbx6_Xl                  (  329) CSNVVVTDVTSNLLTVTIKEFCNAQDFEK  1
Cbx6_Gg                  (  290) CSNVVVTDVTSNLLTVTIKEFCNAEDFEK  1
Cbx6_Mmu                 (  372) CSNVVVTDVTSNLLTVTIKEFCNPEDFEK  1
Cbx6_Hs                  (  365) CSNVVVTDVTSNLLTVTIKEFCNPEDFEK  1
Cbx6_Bt                  (  367) CSNVVVTDVTSNLLTVTIKEFCNPEDFEK  1
Cbx6_Cf                  (  368) CSNVVVTDVTSNLLTVTIKEFCNPEDFEK  1
Pc_Aa                    (  399) TNQVFITDVTVNLETVTIRECKTERGFFK  1
Pc_Ag                    (  339) TNQVFITDVTVNLETVTIRECKTERGFFK  1
Pc_Dy                    (  349) SNRVVITDVTVNLETVTIRECKTERGFFR  1
Pc_Dps                   (  333) SNRVVITDVTVNLETVTIRECKTERGFFR  1
Pc_Dpe                   (  333) SNRVVITDVTVNLETVTIRECKTERGFFR  1
Pc_Dsi                   (  334) SNRVVITDVTVNLETVTIRECKTERGFFR  1
Pc_Dm                    (  350) SNRVVITDVTVNLETVTIRECKTERGFFR  1
Pc_Dse                   (  350) SNRVVITDVTVNLETVTIRECKTERGFFR  1
Cbx6_Mm                  (  367) CSNVVVTDVTSNLLTVTIKEFCSPEDFEK  1
Cbx6_Rn                  (  367) CSNVVVTDVTSNLLTVTIKEFCSPEDFEK  1
Pc_Nvi                   (  563) ADQVFITDVTVNLKTVTIRECKTEKGFFR  1
Pc_Am                    (  391) ADQVFITDVTVNLKTVTIRECKTEKGFFR  1
Cbx8_Dr1                 (  330) VEKVIVTDVTTNFLTVTIKESNTDEGFFK  1
```

```
Pc_Tc                         (  351) ADQVFITDVTVNLKTVTIRECKTEKGFFK  1
Cbx8_Xl                       (  330) LEKVLVTDVTSNFLTVTIKESSTDQGFFK  1
Cbx8_Tn                       (  322) VEKVLVTDVTSNFLTVTIKESSTSQGFFR  1
Cbx7_Mmu                      (  217) SSEVTVTDITANSITVTFREAQAAEGFFR  1
Cbx7_Pt                       (  252) SSEVTVTDITANSITVTFREAQAAEGFFR  1
Cbx7_Bt                       (  217) SSEVTVTDITANSITVTFREAQAAEGFFR  1
Cbx7_Hs1                      (  217) SSEVTVTDITANSITVTFREAQAAEGFFR  1
Cbx7_Md                       (  124) PSEVTVTDITANSITVTFREAQAAEGFFR  1
Cbx7_Ec                       (  317) PSEVTVTDITANSITVTFREAQAAEGFFR  1
Cbx8_Dr2                      (  311) MEKIVVTDVTSNSLTVTIKESSTDKGFFK  1
Cbx7_Mm                       (  217) SSEVTVTDITANSVTVTFREAQAAEGFFR  1
Cbx7_Rn                       (  217) SSEVTVTDITANSVTVTFREAQAAEGFFR  1
Cbx7_Gg                       (  187) PSEVTVTDITANSITVTFREAQVAEGFFR  1
Cbx7_Cf                       (  215) ASEVTVTDITANSVTVTFREAQAAEGFFR  1
Cbx8_Tn2                      (  247) LDTVTVTDVTVNLLTVTVRESSSEKGFFR  1
Cbx8_Fr                       (  299) LDTVTVTDVTVNLLTVTVRESSSEKGFFR  1
Pc_Pc                         (  195) IDQIVITDVTSNDVTITFKESFTEVGFFR  1
Cbx7_Xt                       (  211) PSEIIVTDITSNSITVTFREARSAEGFFR  1
Cbx7_Dr1                      (  359) PGKVIVTQVTINSLTVTFKEAMTAEGFFS  1
Cbx7_Dr2                      (  205) PEEVMVTDITINSLTVTFREALVARGFFS  1
Cbx7_Tn                       (  231) PGEVTVTDVTLNALTVTFRESTRAKDFFR  1
Cbx6_Dr                       (  390) CANVVVTDITANLLTVTIKEFCHPPSASS  1
Cbx6_Fr                       (  423) CKDVVVTDVTTNLVTVTIKEFPSHASPSA  1
Pc_Nve2                       (  305) ANSIIITDVTTERGMITIKECSACGDFFG  1
//
```

```
Motif  3 width=21 seqs=65
Cbx6_Mmu                   (    1) MELSAVGERVFAAESIIKRRI  1
Cbx8_Dr2                   (    1) MELSAVGERVFAAESIIKRRI  1
Cbx8_Tn                    (    1) MELSAVGERVFAAESIIKRRI  1
Cbx8_Dr1                   (    1) MELSAVGERVFAAESIIKRRI  1
Cbx6_Hs                    (    1) MELSAVGERVFAAESIIKRRI  1
Cbx6_Bt                    (    1) MELSAVGERVFAAESIIKRRI  1
Cbx6_Mm                    (    1) MELSAVGERVFAAESIIKRRI  1
Cbx6_Rn                    (    1) MELSAVGERVFAAESIIKRRI  1
Cbx6_Cf                    (    1) MELSAVGERVFAAESIIKRRI  1
Cbx6_Xt                    (    1) MELSAVGERVFAAESIMKRRI  1
Cbx6_Xl                    (    1) MELSAVGERVFAAESIMKRRI  1
Cbx7_Xt                    (    1) MELSAIGEQVFAVESIRKKRI  1
Cbx4_Xt                    (    1) MELPAVGEHVFAVESIEKKRI  1
Cbx8_Fr                    (    1) MELSAVGESVFAAESIIKRRI  1
Cbx4_Gg                    (    1) MELPAVGEHVFAVESIEKKRI  1
Cbx4_Rn                    (    1) MELPAVGEHVFAVESIEKKRI  1
Cbx4_Xl                    (    1) MELPAVGEHVFAVESIEKKRI  1
Cbx4_Mm                    (    1) MELPAVGEHVFAVESIEKKRI  1
Cbx4_Hs                    (    1) MELPAVGEHVFAVESIEKKRI  1
Cbx8_Xt                    (    1) MELSAVGERVFAAESLLKRRI  1
Cbx8_Xl                    (    1) MELSAVGERVFAAESLLKRRI  1
Cbx7_Md                    (    1) MELSAIGEQVFAVESIRKKRV  1
Cbx7_Mmu                   (    1) MELSAIGEQVFAVESIRKKRV  1
Cbx7_Mm                    (    1) MELSAIGEQVFAVESIRKKRV  1
Cbx7_Rn                    (    1) MELSAIGEQVFAVESIRKKRV  1
Cbx7_Pt                    (    1) MELSAIGEQVFAVESIRKKRV  1
Cbx7_Bt                    (    1) MELSAIGEQVFAVESIRKKRV  1
Cbx7_Cf                    (    1) MELSAIGEQVFAVESIRKKRV  1
Cbx7_Hs1                   (    1) MELSAIGEQVFAVESIRKKRV  1
Cbx8_Tn2                   (    1) MELSAVGESVFAAESIMKRRI  1
Cbx8_Ec                    (    1) MELSAVGERVFAAEALLKRRI  1
Cbx8_Bt                    (    1) MELSAVGERVFAAEALLKRRI  1
Cbx8_Mm                    (    1) MELSAVGERVFAAEALLKRRI  1
Cbx8_Rn                    (    1) MELSAVGERVFAAEALLKRRI  1
Cbx8_Cf                    (    1) MELSAVGERVFAAEALLKRRI  1
Cbx8_Hs                    (    1) MELSAVGERVFAAEALLKRRI  1
Cbx7_Dr2                   (    1) MELSAIGEQVFAVESITKKRV  1
Cbx7_Dr1                   (    1) MELSSIGEQVFAVESITKKRI  1
Cbx7_Tn                    (    1) MELSAIGEQVFAVEAIVKKRV  1
Cbx2_Xl                    (    2) EELSAVGEQVFAAECILSKRL  1
Cbx6_Dr                    (    1) MELSAAGDRVFAAEAILKRRV  1
Cbx4_Dr                    (    1) MELPAVGEHVFAVEGIEKKRL  1
```

```
Cbx4_Tn                 (    1) MELPAAGEHVFAVEGIEKKRI  1
Cbx4_Fr1                (    1) MELPAAGEHVFAVEGIEKKRI  1
Cbx2_Tn1                (    2) EELSAVGEQVFDAECILNKRL  1
Cbx2_Fr                 (    2) EELSAVGEQVFDAECILNKRL  1
Cbx2_Md                 (    2) EELSSVGEQVFAAECILSKRL  1
Cbx2_Pt                 (    2) EELSSVGEQVFAAECILSKRL  1
Cbx2_Rn                 (    2) EELSSVGEQVFAAECILSKRL  1
Cbx2_Mm                 (    2) EELSSVGEQVFAAECILSKRL  1
Cbx2_Hs                 (    2) EELSSVGEQVFAAECILSKRL  1
Cbx6_Tn                 (    1) MELSAAGDRIFAAEAILKRRV  1
Cbx6_Fr                 (    1) MELSAAGDRIFAAEAILKRRV  1
Cbx2_Dr                 (    2) EELSAVGEQVFDAECILNKRT  1
Cbx7_Ec                 (  101) GSCQPSGEQVFAVESIRKKRV  1
Pc_Dsi                  (   16) NATDDPVDLVYAAEKIIQKRV  1
Pc_Dm                   (   16) NATDDPVDLVYAAEKIIQKRV  1
Pc_Dse                  (   16) NATDDPVDLVYAAEKIIQKRV  1
Pc_Dy                   (   16) NATDDQVDLVYAAEKIIQKRV  1
Pc_Nve1                 (   15) NEPIPPAEGIFAAECILKKRT  1
Pc_Pc                   (    2) EEAPAGPGEVFAAEKILKKRY  1
Pc_Dps                  (   17) VQDDMDDFLVYAAEKIIQKRT  1
Pc_Dpe                  (   17) VQDDMDDFLVYAAEKIIQKRT  1
Pc_Nvi                  (  131) AGSMDLGDRVYAAERITKKRE  1
Pc_Nve2                 (    1) MNKGGRAAGIYAAETILKERV  1
//

Motif  4 width=15 seqs=45
Cbx8_Xt                 (   67) YGPKKRGPKPKTFLL  1
Cbx6_Mmu                (   67) YGPKKRGPKPKTFLL  1
Cbx8_Ec                 (   67) YGPKKRGPKPKTFLL  1
Cbx8_Xl                 (   67) YGPKKRGPKPKTFLL  1
Cbx8_Tn                 (   67) YGPKKRGPKPKTFLL  1
Cbx8_Bt                 (   67) YGPKKRGPKPKTFLL  1
Cbx8_Mm                 (   67) YGPKKRGPKPKTFLL  1
Cbx8_Rn                 (   67) YGPKKRGPKPKTFLL  1
Cbx8_Cf                 (   67) YGPKKRGPKPKTFLL  1
Cbx8_Hs                 (   67) YGPKKRGPKPKTFLL  1
Cbx6_Xl                 (   67) YGPKKRGPKPKTFLL  1
Cbx6_Hs                 (   67) YGPKKRGPKPKTFLL  1
Cbx6_Bt                 (   67) YGPKKRGPKPKTFLL  1
Cbx6_Mm                 (   67) YGPKKRGPKPKTFLL  1
Cbx6_Rn                 (   67) YGPKKRGPKPKTFLL  1
Cbx6_Cf                 (   67) YGPKKRGPKPKTFLL  1
Cbx4_Tn                 (   67) MGYRKRGPKPKHLLL  1
```

```
Cbx6_Dr          (   67) YGPKKRGPKPKTLLL  1
Cbx6_Xt          (   67) YGPKKRGPKPKTFLM  1
Cbx4_Fr2         (   29) MGYRKRGPKPKHLLI  1
Cbx7_Mmu         (   67) SGYRKRGPKPKRLLL  1
Cbx7_Ec          (  167) SGYRKRGPKPKRLLL  1
Cbx7_Gg          (   44) SGYRKRGPKPKRLLL  1
Cbx7_Rn          (   67) SGYRKRGPKPKRLLL  1
Cbx7_Pt          (  102) SGYRKRGPKPKRLLL  1
Cbx7_Bt          (   67) SGYRKRGPKPKRLLL  1
Cbx7_Cf          (   67) SGYRKRGPKPKRLLL  1
Cbx7_Hs1         (   67) SGYRKRGPKPKRLLL  1
Cbx4_Fr1         (  111) VGYRKRGPKPKHLLL  1
Cbx8_Dr2         (   67) YGPKKRGPKPETFLM  1
Cbx8_Tn2         (   67) FGPKKRGPKPETFLL  1
Cbx8_Fr          (   67) FGPKKRGPKPETFLL  1
Cbx8_Dr1         (   67) FGPKKRGPKLKTFLL  1
Cbx7_Xt          (   67) SGCRKRGPKPKRLLL  1
Cbx4_Xl          (   67) MGYRKRGPKPKNNLV  1
Cbx4_Gg          (   67) MGYRKRGPKPKPLVV  1
Cbx4_Rn          (   67) MGYRKRGPKPKPLVV  1
Cbx4_Hs          (   67) MGYRKRGPKPKPLVV  1
Cbx4_Cf          (   29) MGYRKRGPKPKPLVV  1
Cbx7_Mm          (   67) SGYRKRGPKPRRLLL  1
Cbx4_Xt          (   67) MGYRKRGPKPKHHIV  1
Cbx4_Dr          (   67) VGYRKRGPKPKHPLI  1
Cbx4_Mm          (   67) MGYRKRGPKPNPLVV  1
Cbx6_Fr          (   67) HGPKKRGPKPKTSSR  1
Cbx6_Tn          (   67) HGPKKRGPKPKNVAA  1
//
```

```
Motif  5 width=41 seqs=19
Cbx6_Cf                  (  145) GLRPPISPFSETVRIINRKVKPREPKRNRIILNLKVIDKGT  1
Cbx6_Bt                  (  145) GLRPPISPFSETVRIINRKVKPREPKRNRIILNLKVIDKGP  1
Cbx6_Mm                  (  145) GLRPPISPFSETVRIINRKVKPREPKRNRIILNLKVIDKGP  1
Cbx6_Rn                  (  145) GLRPPISPFSETVRIINRKVKPREPKRNRIILNLKVIDKGP  1
Cbx6_Mmu                 (  145) GLRPPISPFSETVRIINRKVKPREPKRNRIILNLKVIDKGA  1
Cbx6_Hs                  (  145) GLRPPISPFSETVRIINRKVKPREPKRNRIILNLKVIDKGA  1
Cbx6_Xt                  (  147) GMRPPVSPFSETVRIINRKAKPREPKRNRIILNLKVIDKGT  1
Cbx6_Xl                  (  147) GMRPPVSPFSETVRIINRKAKPREPKRNRIILNLKVIDKGT  1
Cbx6_Gg                  (   74) GIRPPVSPFSETVRIINRKVKPREPKRSRIILNLKVIDKGG  1
Cbx6_Fr                  (  166) PSRLHVSPFSETVRILNRRVKPREVKRGRIILNLKVIDKAG  1
Cbx6_Tn                  (  179) PSRLHVSPFSETVRILNRRVKPREVKRGRIILNLKVIDKPG  1
Cbx2_Pt                  (  245) PGRGGISWQSSIVHYMNRMTQSQAQAASRLALKAQATNKCG  1
Cbx2_Hs                  (  245) PGRGGISWQSSIVHYMNRMTQSQAQAASRLALKAQATNKCG  1
Cbx2_Rn                  (  249) PSRGGISWQSSIVHYMNRMSQSQAQAASRLALKAQAANKCS  1
Cbx2_Cf                  (  208) PSRGGISWQSSIVHYMNRMSQSQAQAASRLALKAQTAGKCG  1
Cbx6_Dr                  (  166) SSRPTVSAFSETVRILNRKVKPREVKKGRVILNLKVLDKAE  1
Cbx2_Md                  (  243) PNRGSISWQSSIMHYMNRMTQSQAEAANRLALKAQASNKCG  1
Cbx2_Bt                  (  246) PSRGGISWQSSIVHYMNRMSQSQAQAAGRLALRAPATSKCS  1
Cbx8_Mm                  (  123) GLRNTGLPPPGSSTSTCRADPPRDRDRERDRGTSRVDDKPS  1
//
```

```
Motif  6 width=11 seqs=73
Cbx6_Mmu                  (   52) RLIAAFEQKER  1
Cbx6_Hs                   (   52) RLIAAFEQKER  1
Cbx6_Bt                   (   52) RLIAAFEQKER  1
Cbx6_Mm                   (   52) RLIAAFEQKER  1
Cbx6_Rn                   (   52) RLIAAFEQKER  1
Cbx6_Cf                   (   52) RLIAAFEQKER  1
Cbx6_Xt                   (   52) RLIVAFEQKER  1
Cbx6_Xl                   (   52) RLIVAFEQKER  1
Cbx8_Ec                   (   52) RLLAAFEERER  1
Cbx8_Bt                   (   52) RLLAAFEERER  1
Cbx8_Mm                   (   52) RLLAAFEERER  1
Cbx8_Rn                   (   52) RLLAAFEERER  1
Cbx8_Cf                   (   52) RLLAAFEERER  1
Cbx8_Hs                   (   52) RLLAAFEERER  1
Cbx6_Dr                   (   52) RLVAAFEQKER  1
Cbx4_Fr2                  (   14) RLLDAFQERER  1
Cbx4_Xt                   (   52) RLLVAFQNRER  1
Cbx4_Dr                   (   52) RLLVAFQNRER  1
Cbx4_Xl                   (   52) RLLVAFQNRER  1
Cbx8_Tn2                  (   52) RLFAAFEERER  1
Cbx8_Fr                   (   52) RLFAAFEERER  1
Cbx8_Dr2                  (   52) RLFAAFEERER  1
Cbx8_Tn                   (   52) RLFVAFEQRER  1
Cbx8_Dr1                  (   52) RLFVAFEERER  1
Cbx4_Gg                   (   52) RLLIAFQNRER  1
Cbx4_Rn                   (   52) RLLIAFQNRER  1
Cbx4_Mm                   (   52) RLLIAFQNRER  1
Cbx4_Hs                   (   52) RLLIAFQNRER  1
Cbx4_Cf                   (   14) RLLIAFQNRER  1
Cbx7_Mmu                  (   52) RLVMAYEEKEE  1
Cbx7_Ec                   (  152) RLVMAYEEKEE  1
Cbx7_Mm                   (   52) RLVMAYEEKEE  1
Cbx7_Rn                   (   52) RLVMAYEEKEE  1
Cbx7_Bt                   (   52) RLVMAYEEKEE  1
Cbx7_Cf                   (   52) RLVMAYEEKEE  1
Cbx7_Hs1                  (   52) RLVMAYEEKEE  1
Cbx2_Md                   (   53) RLLLAFQKKEH  1
Cbx2_Mmu                  (   76) RLLLAFQKKEH  1
Cbx4_Tn                   (   52) RLLVAFQHRER  1
Cbx2_Cf                   (   19) RLLLAFQKKEH  1
Cbx2_Rn                   (   53) RLLLAFQKKEH  1
Cbx2_Mm                   (   53) RLLLAFQKKEH  1
```

```
Cbx2_Hs                    (   53) RLLLAFQKKEH  1
Cbx8_Xt                    (   52) RLVAAFEDRER  1
Cbx8_Xl                    (   52) RLVAAFEDRER  1
Cbx7_Gg                    (   29) RLVVAYEEKEE  1
Cbx2_Xl                    (   53) RLLVAFQKREQ  1
Cbx7_Dr2                   (   52) RLVLAYEEKEQ  1
Cbx7_Xt                    (   52) RLVLAYEEKEE  1
Cbx2_Fr                    (   53) RLLAAFNKKEH  1
Cbx6_Tn                    (   52) RLILGFEKKER  1
Cbx6_Fr                    (   52) RLILGFEKKER  1
Cbx2_Dr                    (   53) RLLVAFNKREQ  1
Cbx7_Tn                    (   52) RLVQAYEEKEQ  1
Cbx2_Tn2                   (   51) RLLAAFHKREQ  1
Pc_Nve1                    (   66) RLLLAFENSRR  1
Cbx2_Pt                    (   53) RLLLSFQKKEH  1
Cbx7_Dr1                   (   52) RLVLAFEEKAE  1
Pc_Dps                     (   68) RLIDIYEQSNK  1
Pc_Dpe                     (   68) RLIDIYEQSNK  1
Pc_Dy                      (   67) RLIDIYEQTNK  1
Pc_Dsi                     (   67) RLIDIYEQTNK  1
Pc_Dm                      (   67) RLIDIYEQTNK  1
Pc_Dse                     (   67) RLIDIYEQTNK  1
Cbx4_Fr1                   (   52) RLLVAFQHRIM  1
Cbx7_Md                    (   52) RLVMAYEEKRT  1
Cbx2_Tn1                   (   53) RLLAAFNKNIR  1
Pc_Aa                      (  110) RLIDIFERSLR  1
Pc_Ag                      (   50) RLIDIFERSLR  1
Pc_Nvi                     (  182) RLIELYEESQR  1
Pc_Nve2                    (   52) RLLKAYQERLA  1
Pc_Tc                      (   49) RLIDLFERSQK  1
Pc_Am                      (   87) RLIELYEESQK  1
//
```

```
Motif  7 width=41 seqs=12
Cbx6_Mmu                 (  104) SASSPKLHSSAAVHRLKKDIRRCHRMSRRPLPRPDPQGGSP  1
Cbx6_Hs                  (  104) SASSPKLHSSAAVHRLKKDIRRCHRMSRRPLPRPDPQGGSP  1
Cbx6_Bt                  (  104) SASSPKLHSSAAVHRLKKDIRRCHRMSRRPLPRPDPQGGSP  1
Cbx6_Mm                  (  104) SASSPKLHSSAAVHRLKKDIRRCHRMSRRPLPRPDPQGGSP  1
Cbx6_Cf                  (  104) SASSPKLHSSAAVHRLKKDIRRCHRMSRRPLPRPDPQGGSP  1
Cbx6_Rn                  (  104) SASSPKLHSSAAVHRLKKDIRRCHRMSRRPLPRPDPQGGNP  1
Cbx6_Gg                  (   28) STSSPKLHSSAAVHRLKKDIRRCHRMSRRPLPRPDPQNGGN  1
Cbx6_Xt                  (  103) SSPSPKLHSSAAVHRLKKDIRRCHSMSRRPLPRPDPTAPSG  1
Cbx6_Xl                  (  103) SSPSPKLHSSAAVHRLKKDIRRCHSMSRRPLPRPDPTAPSG  1
Cbx6_Tn                  (  132) FAPSPKLNSLAATHKLKKDIHRCHRMSRRPLPRSDPMAGSF  1
Cbx6_Fr                  (  119) FAPSAKLNSHAATHKLKKDIHRCHRMSRRPLPRSDPMAGSF  1
Cbx6_Dr                  (  122) APSNAKLQSGTAQPKLKKDIHRCHRMARRPLPRQDHTVGPS  1
//

Motif  8 width=29 seqs=20
Cbx4_Gg                  (  246) KMKIVKNKNKNGRIVIVMSKYMENGMQAV  1
Cbx4_Mm                  (  249) KMKIVKNKNKNGRIVIVMSKYMENGMQAV  1
Cbx4_Hs                  (  249) KMKIVKNKNKNGRIVIVMSKYMENGMQAV  1
Cbx4_Cf                  (  211) KMKIVKNKNKNGRIVIVMSKYMENGMQAV  1
Cbx4_Xt                  (  239) KMKIVKNKNKNGRIVIVMSKYMENGMQSV  1
Cbx4_Xl                  (  253) KMKIVKNKNKNGRIVIVMSKYMENGMQSV  1
Cbx4_Tn                  (  254) KMKIIKNKNKNGRIVIVMSKYMDNNKVHG  1
Cbx4_Fr1                 (  298) KMKIIKNKNKNGRIVIVMSKYMDNNKVHG  1
Cbx4_Dr                  (  249) KMKIIKNKNKNGRIVIVMSKYMDKGVHSS  1
Cbx4_Fr2                 (  213) KLKIVKNKNKNGRIVIVMSKYMENGIHPA  1
Cbx6_Cf                  (  206) NRVIGKSKKFSESILRTQIRHMKFGTFSL  1
Cbx6_Bt                  (  206) NRVIGKSKKFSESILRTQVRHMKFGTFAL  1
Cbx6_Mm                  (  206) NRVIGKSKKFSESMLRTQIRHMKFGTFAL  1
Cbx6_Rn                  (  206) NRVIGKSKKFSESMLRTQIRHMKFGTFAL  1
Cbx4_Rn                  (  249) KMKIVKNKNKNGRIVIVMSKTFKKAAGAS  1
Cbx6_Mmu                 (  206) NRVIGKSKKFSESVLRTQIRHMKFGAFAL  1
Cbx6_Hs                  (  206) NRVIGKSKKFSESVLRTQIRHMKFGAFAL  1
Pc_Am                    (   28) MMVLNLDSDELGMIRTVVKRTMQKSIISI  1
Cbx7_Dr1                 (  164) CTENAWNEDEQKRKVKKMRKDEENTTQVH  1
Cbx6_Xl                  (  188) KAPLARPKIPSRNRVIGKSKKYSESMLRN  1
//
```

```
Motif  9 width=50 seqs=8
Cbx7_Mmu                 (  140) RKPRKAHKYLRLSRKKFPPRGPNLESHSHRRELFLQEPPAPDVLQAAGEW  1
Cbx7_Pt                  (  175) RKPRKAHKYLRLSRKKFPPRGPNLESHSHRRELFLQEPPAPDVLQAAGEW  1
Cbx7_Hs1                 (  140) RKPRKAHKYLRLSRKKFPPRGPNLESHSHRRELFLQEPPAPDVLQAAGEW  1
Cbx7_Ec                  (  240) RKPRKAHKYLRLSRKKFPPRGPNLESHSHRRELFLQESPAPDVLQATGEW  1
Cbx7_Bt                  (  140) RKPRKAHKYLRLSRKKFPPRGPNLESHSHRRELFLQESPAQDVLQAASEW  1
Cbx7_Cf                  (  139) RKPRKAHKYLRLSRKKFPPRGPDLESHSHPRELFLQEPAAPDVLQAASEW  1
Cbx7_Mm                  (  140) RKARKAHKYLRLSRKKFPPRGPHLESHSHRRELSLQESAAPDVVQTPGDW  1
Cbx7_Rn                  (  140) RKPHKAHKYLRLSHKKFPPCGSHLESHSHRRELSLQESAAPDVLQATGDW  1
//

Motif  10 width=40 seqs=17
Cbx7_Mmu                 (   82) QRLYSMDLRSSHKAKGKEKLCFSLTCPLGSGSPEGVVKAG  1
Cbx7_Pt                  (  117) QRLYSMDLRSSHKAKGKEKLCFSLTCPLGSGSPEGVVKAG  1
Cbx7_Hs1                 (   82) QRLYSMDLRSSHKAKGKEKLCFSLTCPLGSGSPEGVVKAG  1
Cbx7_Ec                  (  182) QRLYSMDLRSSHKAKGKEKLCFSLTRPLGSGSPEGVVKAG  1
Cbx7_Cf                  (   82) QRLYSMDLRSSHKAKGKEKLCFSLTRPLGSGSPEGVVKAG  1
Cbx7_Bt                  (   82) QRLYSMDLRSSHKAKGKEKLCFSLTRPLGSGSPKGVVKAG  1
Cbx7_Mm                  (   82) QRLYSMDLRSSHKAKGNEKLCFSLARPLRSGSPMGVVKAG  1
Cbx7_Rn                  (   82) QRLYSMDLRSSHKAKGKEKLCFSLACPLGNGSPKGVVKAG  1
Cbx2_Pt                  (  107) SSSSSSSTSSSSSSDEEDDSDLDAKRGPRGRETHPVPQKK  1
Cbx2_Hs                  (  107) SSSSSSSTSSSSSSDEEDDSDLDAKRGPRGRETHPVPQKK  1
Cbx2_Mmu                 (  130) SSSSSSSTSSSSSSDEEDDSDLDAKRGPRGRDTHPVPQKK  1
Cbx2_Bt                  (  108) SSSSSSSTSSSSSSEEEEDSDLDAKRGPRGRETHPVPQKK  1
Cbx2_Cf                  (   70) SSSSSSSTSSSTSSDEEDDSDLDAKRGPRGRETHPVPQKK  1
Cbx2_Md                  (  106) SSSSSSSSTSSSSSDEEDDSDLEAKRGPRGRETHPVPQKK  1
Cbx2_Mm                  (  108) SSSSSSTSSSSSSDEEEDDSDLDSKRGPRGRETHPVPQKK  1
Cbx2_Gg                  (   51) SSSSSSTSSSSSSSDEEDESDLEAKRGPRSRETHPVPQKK  1
Cbx2_Rn                  (  111) SSSTSSSSSSDEEEEDDDDSDLDSKRGSRGRETHPVPQKK  1
//

Motif  11 width=50 seqs=9
Cbx2_Bt                  (  148) AQILVAKPELKDPIRKKRGRKPLPPEQKAARRPVSLAKVLKTARKDLGAP  1
Cbx2_Cf                  (  110) AQILVAKPELKDPIRKKRGRKPLPPEQKAARRPVSLAKVLKTARKDLGAP  1
Cbx2_Mmu                 (  170) AQILVAKPELKDPIRKKRGRKPLPPEQKATRRPVSLAKVLKTARKDLGAP  1
Cbx2_Pt                  (  147) AQILVAKPELKDPIRKKRGRKPLPPEQKATRRPVSLAKVLKTARKDLGAP  1
Cbx2_Hs                  (  147) AQILVAKPELKDPIRKKRGRKPLPPEQKATRRPVSLAKVLKTARKDLGAP  1
Cbx2_Mm                  (  148) AQILVAKPELKDPIRKKRGRKPLPPEQKAARRPVSLAKVLKTTRKDLGTS  1
Cbx2_Rn                  (  151) AQILVAKPELKDPIRKKRGRKPLPPEQKAARRPVSLAKVLKTTRKDLVTS  1
Cbx2_Md                  (  146) AQILVAKPEVKDPIRKKRGRKPLPPEQKAARRPVSLPKVLKTTRKDLGPS  1
Cbx2_Gg                  (   91) AQILVAKPEMKDSSRKKRGRKPLPPEQKAARRTVNLTKVLKTSRKEVGGS  1
//
```

```
Motif  12 width=31 seqs=11
Cbx4_Rn                  (  481) LSEFKPFFGNIIITDVTANCLTVTFKEYVTV  1
Cbx4_Mm                  (  521) LSEFKPFFGNIIITDVTANCLTVTFKEYVTV  1
Cbx4_Hs                  (  530) LSEFKPFFGNIIITDVTANCLTVTFKEYVTV  1
Cbx4_Cf                  (  485) LSEFKPFFGNIIITDVTANCLTVTFKEYVTV  1
Cbx4_Gg                  (  493) LREFKPFFGNIIITDVTANCLTVTFKEYVTV  1
Cbx4_Xt                  (  477) ESEFKPFFGNIVITDVTANCLTVTFKEYITV  1
Cbx4_Xl                  (  491) ESQFKPFFGNIVITDVTANCLTVTFKEYITV  1
Cbx4_Dr                  (  447) DSQCKAFVGNVIITDITTNCLTVTFKEYVQE  1
Cbx4_Fr1                 (  558) VKKISPFMGNIIITDITTNSLTVTFKEYVSF  1
Cbx4_Fr2                 (  472) FPSFQPLLGNIVITDITTNCLTVTFKEYVAA  1
Cbx4_Tn                  (  514) AKKVSPFMGNIVITDITTNSLTVTFKEYISF  1
//

Motif  13 width=34 seqs=11
Cbx4_Rn                  (  118) HQYELNSKKHHQYQPHGKERSGKPPPPGKSGKYY  1
Cbx4_Hs                  (  118) HQYELNSKKHHQYQPHSKERAGKPPPPGKSGKYY  1
Cbx4_Cf                  (   80) HQYELNSKKHHQYQPHSKERAGKPPPPGKSGKYY  1
Cbx4_Xl                  (  116) HQYQLNSKKHHQYQPSGKDHNEKHHSSNKKKYYY  1
Cbx4_Xt                  (  118) HQYQLNSKKHHQYQPSGKDHNEKHHLNTKKKYYY  1
Cbx4_Fr1                 (  164) QQYQLNSKKHHQYQPSNQEVPTDQQMNGKKKLIY  1
Cbx4_Mm                  (  118) HQYELNSKKHHQYQPHSKERSGKPPPARKSGKYY  1
Cbx4_Tn                  (  120) QQYQLNSKKHHQYQPSNQEVPADQQANGKKKFIY  1
Cbx4_Dr                  (  120) QHYQLNSKKHHQYQPSCKEISVEQHMSGKKKHFY  1
Cbx4_Gg                  (  118) HQYELNSKKHHQYQPNGKESGVKHQSHSKGKYYY  1
Cbx4_Fr2                 (   82) QQYQLNSQKHHQYPPVCREQDGDRQSSGKKFYYQ  1
//

Motif  14 width=34 seqs=11
Pc_Dm                    (  133) KHHHHHHHHHHIKSERNSGRRSESPLTHHHHHHH  1
Pc_Dse                   (  133) KHHHHHHHHHHIKSERNSGRRSESPLTHHHHHHH  1
Pc_Dy                    (  132) KKHHHHHHHHHIKSERNSGRRSESPLTHHHHHHH  1
Pc_Dps                   (  134) KKHHHHHHHHHIKSERSSSRRSESPLTHHHHHHH  1
Pc_Dpe                   (  134) KKHHHHHHHHHIKSERSSSRRSESPLTHHHHHHH  1
Cbx4_Mm                  (  384) HHHHHHHHHHHHTVGLNLSHARKRCLSETHGERE  1
Cbx4_Hs                  (  389) HHHHHHHHHHHHAVGLNLSHVRKRCLSETHGERE  1
Cbx4_Rn                  (  344) HHFHHHHHHHHHTVGLNLSHARKRCLSETHGERE  1
Cbx4_Cf                  (  347) HHHHHHKHHHHHAVGKNLSHARKRCLSETHGERE  1
Pc_Dsi                   (  133) KHHHHHHHHHHIKSERNSGRRSGISVEPPSSSPP  1
Cbx8_Tn                  (  227) RHHHHHHHYHHHSAPGGSYRRLYDRGLHTHRRDT  1
//
```

```
Motif  15 width=29 seqs=13
Cbx8_Mm                  (   84) QAKAKAKTYEFRSDSTRGIRIPYPGRSPQ  1
Cbx8_Rn                  (   84) QAKAKAKTYEFRSDSTRGIRIPYPGRSPQ  1
Cbx8_Ec                  (   84) QAKAKAKTYEFRSDSARGIRIPYPGRSPQ  1
Cbx8_Bt                  (   84) QAKAKAKTYEFRSDSARGIRIPYPGRSPQ  1
Cbx8_Cf                  (   84) QAKAKAKTYEFRSDSARGIRIPYPGRSPQ  1
Cbx8_Hs                  (   84) QAKAKAKTYEFRSDSARGIRIPYPGRSPQ  1
Cbx8_Xt                  (   84) QAKANAKTYEFRSESTRGVRIPYPGHPPQ  1
Cbx8_Xl                  (   84) QAKANAKTYEFRTESTRGVRFPYPGHPSQ  1
Cbx8_Fr                  (   82) KAKAKEKSYDFRGDAFRGIQVSYPVPEPV  1
Cbx8_Dr1                 (   84) QAKEKAKSYEFRNDSSRGIHVTYSSPEPV  1
Cbx8_Tn                  (   84) QAKEKVKSYDFRSESVRGMHISYPTPEPV  1
Cbx8_Tn2                 (   82) KAKAKERAYDFRGDAFRGIQVSYPVPEPV  1
Cbx8_Dr2                 (   84) KAKAKSKTYEFGREMSRDIRVSFPVAEPV  1
//

Motif  16 width=41 seqs=8
Cbx2_Bt                  (  202) PPPLSAPVAGLAALKAHAKEACGGPSAMATPENLASLMKGM  1
Cbx2_Hs                  (  201) PPPLSAPVAGLAALKAHAKEACGGPSAMATPENLASLMKGM  1
Cbx2_Rn                  (  205) PPPLSAPVAGLAALKAHTKEACGGPSTMATPENLASLMKGM  1
Cbx2_Mm                  (  202) PPPLSAPVAGLAALKAHTKEACGGPSTMATPENLASLMKGM  1
Cbx2_Mmu                 (  224) PPPLSAPVAGLAALKAHAKEACGSPSAMATPENLASLMKGM  1
Cbx2_Pt                  (  201) PPPLSAPVAGLAALKAHAKEACGSPSAMATPENLASLMKGM  1
Cbx2_Cf                  (  164) PPPLSAPVAGLAALKAHAKEACSGPGAMATPENLASLMKGM  1
Cbx2_Md                  (  199) PPSLTTPVTSLAALKAHAKEAPGGSSGSSTAENLTNLMKGM  1
//

Motif  17 width=29 seqs=13
Cbx2_Rn                  (   64) EKEVQNRKRGKRPRGRPRKHTVMSSCSRR  1
Cbx2_Bt                  (   65) EKEVQNRKRGKRPRGRPRKHTVMSSCSRH  1
Cbx2_Mm                  (   64) EKEVQNRKRGKRPRGRPRKHTVTSSCSRR  1
Cbx2_Mmu                 (   87) EKEVQNRKRGKRPRGRPRKLTAMSSCSRR  1
Cbx2_Pt                  (   64) EKEVQNRKRGKRPRGRPRKLTAMSSCSRR  1
Cbx2_Hs                  (   64) EKEVQNRKRGKRPRGRPRKLTAMSSCSRR  1
Cbx2_Cf                  (   30) EKEVQNRKRGKRPRGRPRKHTVMSCSRHS  1
Cbx2_Md                  (   64) DKEVQNRKRGKRPRGRPRKHVVVSACNRR  1
Cbx2_Gg                  (   20) EKEVQNRKRGKRPRGRPRKHVEPEMPSKS  1
Cbx2_Xl                  (   64) EKELRNRKRGKRPRGRPRKNVETDIPLKA  1
Cbx2_Fr                  (   64) EKELLMRKKGKRPRGRPRKILENLPEPEK  1
Cbx2_Dr                  (   64) EKELLISKKGKRPRGRPRKIMETIPVVSK  1
Cbx2_Tn2                 (   62) ERELLFQKKGKRPRGRPRKHPLPEVAADR  1
//
```

```
Motif  18 width=29 seqs=8
Cbx8_Ec                  (  321) DMGAQGGRPSLIARIPVARILGDPEEESW  1
Cbx8_Bt                  (  311) DMGAQGGRPSLIARIPVARILGDPEEESW  1
Cbx8_Mm                  (  296) DMGAQGGRPSLIARIPVARILGDPEEESW  1
Cbx8_Rn                  (  300) DMGAQGGRPSLIARIPVARILGDPEEESW  1
Cbx8_Cf                  (  313) DMGAQGGRPSLIARIPVARILGDPEEESW  1
Cbx8_Hs                  (  323) DMGAQGGRPSLIARIPVARILGDPEEESW  1
Cbx8_Xt                  (  296) ALGTHGGKPSLIARIPVARILGEPEEEPW  1
Cbx8_Xl                  (  295) ALGTHGGKPSLIARIPVARILGEPEEEPW  1
//

Motif  19 width=50 seqs=8
Cbx8_Ec                  (  236) AGKFPAGHSVIQLARRQDSDLAQCGVASPSPAEATGKLAVDTFPARVIKH  1
Cbx8_Bt                  (  226) AGKFPAGHSVIQLARRQDSDLAQCGVASPSPAEATGKLAVDTFPARVIKH  1
Cbx8_Cf                  (  228) AGKFPAGHSVIQLARRQDSDLAQCGVASPSPAEATGKLAVDTFPARVIKH  1
Cbx8_Hs                  (  238) AGKFPAGHSVIQLARRQDSDLVQCGVTSPSSAEATGKLAVDTFPARVIKH  1
Cbx8_Mm                  (  211) TGKFPAGHSVIQLARRQDSDLVQYGVTSPSSAEASSKLAVDTFPARVIKH  1
Cbx8_Rn                  (  215) IGKFSAGHSVIQLARRQDSDLVQYGVTSPSSAEASGKLAADTFPARVIKH  1
Cbx8_Xt                  (  203) AGKYHAPHSVIQLARRQDTDLGPSSHVGPGFMHRERALTQTGQDIQEQLS  1
Cbx8_Xl                  (  203) AEKYHATHSVIQLARRQDTELVSSNQAGPGFVRRERGLTQTGQDIQQLNT  1
//

Motif  20 width=34 seqs=9
Cbx2_Pt                  (  336) VPAGCPGPQPAPTQELSLQVLDLQSVKNGMPGVG  1
Cbx2_Hs                  (  336) VPAGCPGPQPAPTQELSLQVLDLQSVKNGMPGVG  1
Cbx2_Mmu                 (  357) IPAGCPGPQSAPTQELSLQVLDLQSVKNGMPGVG  1
Cbx2_Cf                  (  299) GPAGCLGPQPAPTQELSLQVLDLQSVKNGTAGVG  1
Cbx2_Mm                  (  324) NHSGSPGAQLAPTQELSLQVLDLQSVKNGVPGVG  1
Cbx2_Bt                  (  336) VLAGCLGPQPAPPQELSLQVLDLQSVKNGTPAGG  1
Cbx2_Rn                  (  327) NNSGSPGSQLPPTQELSLQVLDLQSVKNGVPGVG  1
Cbx2_Md                  (  334) VGTMSLGTQPASNQELNLQALNLQSVKNGATGGA  1
Cbx2_Gg                  (  291) TSSSVPGGQQASSQELNLQALNLQSVKNGQSAAG  1
//
```

```
Motif  21 width=35 seqs=9
Cbx6_Cf                   (  291) PDDVPPKLLPETMSPSAPDWREPEVLDLSIPPEAA  1
Cbx6_Gg                   (  216) PDDSPPKLLPETLSPAIPDWRESEVLDLSIPPESA  1
Cbx6_Mm                   (  289) PDDAPPKLLPETLSRSVPNWRESEVLDLSIPPEAA  1
Cbx6_Mmu                  (  295) PDDTPPKLLPETVSPSAPSWREPEVLDLSLPPESA  1
Cbx6_Rn                   (  289) PDDAPPKLLPETISRSASNWRESEVLDLSIPPEAA  1
Cbx6_Bt                   (  290) PEDAPPKLLPETTSPSAPDWREPEVLDLSLPPEAA  1
Cbx6_Hs                   (  288) PDDTLPKLLPETVSPSAPSWREPEVLDLSLPPESA  1
Cbx6_Xt                   (  267) SDDRPPKLMPETLSPAVPDWRESEALDLSIPPESS  1
Cbx6_Xl                   (  267) SDDRPPKLMPETLSPAVPDWRESEALDLSIPPESS  1
//

Motif  22 width=29 seqs=10
Cbx2_Bt                   (  467) DSDPDSASLPSAGQNLSVSVQTSQDWKPT  1
Cbx2_Mmu                  (  489) DSDPDSASPPSTGQNPSVSVQTSQDWKPT  1
Cbx2_Pt                   (  468) DSDPDSASPPSTGQNPSVSVQTSQDWKPT  1
Cbx2_Hs                   (  468) DSDPDSASPPSTGQNPSVSVQTSQDWKPT  1
Cbx2_Rn                   (  459) DSDPDSSSLPSAGQNLSVAVQTSQDWKPT  1
Cbx2_Gg                   (  420) DSDRDSASFPSVGQNMSVSIQTSQDWKPT  1
Cbx2_Mm                   (  455) DSDPDSTSLPSAAQNLSVAIQTSQDWKPT  1
Cbx2_Cf                   (  430) DSDPGSASPPGARQNPSVSVQTSQDWKPT  1
Cbx2_Md                   (  463) SERYSASFPGTTGQNLSVSIQTSQDWKPT  1
Cbx2_Dr                   (  446) SDTDHDSSFPRDSHDLSISVQAGQDWRPT  1
//

Motif  23 width=18 seqs=10
Cbx4_Dr                   (  154) QLNSKKHHHYQPDPKMYD  1
Cbx4_Xt                   (  152) QLNSKKHHHYQPDPKMYE  1
Cbx4_Gg                   (  152) QLNSKKHHHYQPDPKMYE  1
Cbx4_Rn                   (  153) QLNSKKHHPYQPDPKMYD  1
Cbx4_Mm                   (  153) QLNSKKHHPYQPDPKMYD  1
Cbx4_Hs                   (  153) QLNSKKHHPYQPDPKMYD  1
Cbx4_Cf                   (  115) QLNSKKHHPYQPDPKMYD  1
Cbx4_Xl                   (  150) QLNSKKHHHYQPDPKLYE  1
Cbx4_Tn                   (  154) QLNSKKHHHYEPDLNMYE  1
Cbx4_Fr1                  (  198) QLNSKKHHHYEPDLNMYE  1
//
```

```
Motif  24 width=50 seqs=6
Pc_Dsi                   (  284) GNQQTPQVPSENNNIPKPCNNLAINQKQPLTPLSPRALPPRFWLPAKCNI  1
Pc_Dse                   (  300) GNQQTPQVPSENNNIPKPCNNLAINQKQPLTPLSPRALPPRFWLPAKCNI  1
Pc_Dy                    (  299) GNQQAPQVPSENNNIPKPCNNLAINQKQPLTPLSPRALPPRFWLPAKCNI  1
Pc_Dm                    (  300) GNQQAPQVPSENNNIPKPCNNLAINQKQPLTPLSPRALPPRFWLPAKCNI  1
Pc_Dps                   (  283) EEVEESGAQPDNNNIPKLSNTLSLSQKQPLTPLSPRALPPRFWLPAKCNI  1
Pc_Dpe                   (  283) EEVEESGAQPDNNNIPKLSNTLSLSQKQPLTPLSPRALPPRFWLPAKCNI  1
//

Motif  25 width=41 seqs=6
Pc_Dy                    (   83) SKRGIKKKEKEPDPEPESEEDEYTFTGDDVDTHQATTSTAT  1
Pc_Dsi                   (   83) SKRGIKKKEKEPDPEPESEEDEYTFTVDDVDTHQATTSSAT  1
Pc_Dse                   (   83) SKRGIKKKEKEPDPEPESEEDEYTFTVDDVDTHQATTSSAT  1
Pc_Dm                    (   83) SKRGIKKKEKEPDPEPESEEDEYTFTENDVDTHQATTSSAT  1
Pc_Dps                   (   84) SKRGLKKKEKEPDPEPESEEDEYTFTGDDVDTPQATTSSAV  1
Pc_Dpe                   (   84) SKRGLKKKEKEPDPEPESEEDEYTFTGDDVDTPQATTSSAV  1
//

Motif  26 width=50 seqs=5
Pc_Dy                    (  167) ESKRQRMDHSSSSNSSFTHNSFVPEPDSNSSSSEDQPLIGTKRKAEVLKE  1
Pc_Dse                   (  168) ESKRQRMDHSSSSNSSFTHNSFVPEPDSNSSSSEDQPLIGTKRKAEVLKE  1
Pc_Dm                    (  168) ESKRQRIDHSSSSNSSFTHNSFVPEPDSNSSSSEDQPLIGTKRKAEVLKE  1
Pc_Dps                   (  168) ESKRQRMDHSSSSNSSSTHNSFVPEADTNSSSSEDQPLIGTKRKAEVLKE  1
Pc_Dpe                   (  168) ESKRQRMDHSSSSNSSSTHNSFVPEADTNSSSSEDQPLIGTKRKAEVLKE  1
//

Motif  27 width=50 seqs=7
Cbx2_Pt                  (  381) VPATNPAPGKGTGSGLIGASGATMPTDTSKSEKLASRAVAPPTPASKRDC  1
Cbx2_Hs                  (  381) VPATNPAPGKGTGSGLIGASGATMPTDTSKSEKLASRAVAPPTPASKRDC  1
Cbx2_Mmu                 (  402) VPATNPVPGKGTGGGLIGGSGAAMPTDTSKSEKLASRAVAPPTPASKRDY  1
Cbx2_Bt                  (  381) VPATNPATGKGAGGGPTAGSGTGLPTDVSKGEKLVSRAAAMPTPAGKRDC  1
Cbx2_Cf                  (  344) VPATNPTTGKSAGGGLAGGSGATVPSDTSKSEKLASRVAALPTSAGKRDC  1
Cbx2_Mm                  (  368) IPATNPATGKGPGSGPTGANMTNAPTDNNKGEKLTCKATALPAPSVKRDT  1
Cbx2_Rn                  (  372) IPATNTATGKGPGSGPTGVNVTNAPTDTSKGEKLTCKTAALPAPSVKRDA  1
//
```

```
Motif  28 width=49 seqs=7
Cbx4_Rn                 (  408) PTAASLPQPEVILLDSDLDEPIDLRCVKMRSDAGEPPSTLQVKPEAPAV  1
Cbx4_Mm                 (  448) PTAASLPQPEVILLDSDLDEPIDLRCVKMRSDAGEPPSTLQVKPEAPAV  1
Cbx4_Cf                 (  410) PPTATLPQPEVILLDSDLDEPIDLRCVKTRGEAGEPPSALQVKPEAPAA  1
Cbx4_Hs                 (  452) PPAAALPQPEVILLDSDLDEPIDLRCVKTRSEAGEPPSSLQVKPETPAS  1
Cbx4_Xl                 (  435) PLNVPLQEPDIILLDSDLDEPIDLRCVKSRCDSDQEVDKPEIQVTQNPQ  1
Cbx4_Xt                 (  421) PINVPAQEPDIILLDSDLDEPIDLRCVKSRCDSDQEVEKPEVQVTQKPQ  1
Cbx4_Gg                 (  419) ERPELPAQPEVILLDSDLDEPIDLRCVKPRAEGEAALVQVKPEEPPAPA  1
//

Motif  29 width=21 seqs=9
Cbx6_Hs                 (   82) KARAQAEALRISDVHFSVKPS  1
Cbx6_Bt                 (   82) KARAQAEALRISDVHFSVKPS  1
Cbx6_Mm                 (   82) KARAQAEALRISDVHFSVKPS  1
Cbx6_Rn                 (   82) KARAQAEALRISDVHFSVKPS  1
Cbx6_Mmu                (   82) KARAQAEALRISDVHFSVKPT  1
Cbx6_Cf                 (   82) KARAQAEALRISDVHFPVKPS  1
Cbx6_Xt                 (   82) KARAQAEALRLNDVHFTMKPG  1
Cbx6_Gg                 (    6) SARAQAEALHIGDVHFSVKPG  1
Cbx6_Xl                 (   82) KARAQTDALQMSDVHFTMKPG  1
//

Motif  30 width=21 seqs=11
Cbx7_Ec                 (  296) PEEEADADLAEGPPPWTPTLP  1
Cbx7_Mmu                (  196) PEEEADADLAEGPPPWTPALP  1
Cbx7_Pt                 (  231) PEEEADADLAEGPPPWTPALP  1
Cbx7_Hs1                (  196) PEEEADADLAEGPPPWTPALP  1
Cbx7_Mm                 (  196) PEEEAEADLTNGPPPWTPTLP  1
Cbx7_Rn                 (  196) PEEEAEADLTNGPPPWTPMLP  1
Cbx7_Cf                 (  194) PPEEEDADLAEGPPPWTPVLP  1
Cbx7_Bt                 (  196) PEEEAEADLGEGPPSWTPTLP  1
Cbx7_Md                 (  103) PQHETSGDPTEGGPPWIPTHP  1
Cbx6_Tn                 (  407) PPAESKRVPAEGDPDWHPEMA  1
Cbx6_Fr                 (  400) PPAESKRVPAEGDPDWHPEMA  1
//

Motif  31 width=40 seqs=6
Cbx8_Bt                 (  183) SSKKRGPKPRKELLDPSQRPLGEPSDGLGDYLKGRKLDDT  1
Cbx8_Rn                 (  172) SSKKRGPKPRKELLDPSQRPLGEPSDGLGEYLKGRKLDET  1
Cbx8_Ec                 (  193) SSKKRGPKPRKELLDPSQRPLGEASDGLSDYLKGRKLDDT  1
Cbx8_Hs                 (  195) SSKKRGPKPRKELPDPSQRPLGEPSAGLGEYLKGRKLDDT  1
Cbx8_Cf                 (  185) SSKKRGPKPRKELLDPSQRPLGEASDGLGDYLKGRKVDEA  1
Cbx8_Mm                 (  168) SSKKRGPKPRKEPLDPSQRPLGEPSAGLGEYLKGRKLDET  1
```

```
//

Motif  32 width=21 seqs=11
Cbx4_Rn                   (   84) PTFARRSNVLTGLQDSSADNR  1
Cbx4_Mm                   (   84) PTFARRSNVLTGLQDSSADNR  1
Cbx4_Cf                   (   46) PTFARRSNVLTGLQDSSADNR  1
Cbx4_Hs                   (   84) PTFARRSNVLTGLQDSSTDNR  1
Cbx4_Gg                   (   84) PSFARRSNILTGLQDPTVDTR  1
Cbx4_Xl                   (   84) PSFARRSNVLSGLHDSSGENR  1
Cbx4_Dr                   (   84) PAFARRSSILGGLQDTSLDEE  1
Cbx4_Xt                   (   84) PSFARRSNVLNGRNDSPGENR  1
Cbx4_Tn                   (   84) PSFARRSSIPAGLEEPSQDAD  1
Cbx4_Fr1                  (  128) PSFARRSSIPAGLEETCQEAD  1
Cbx4_Fr2                  (   46) PSFARRSSVLADIREVSLEEG  1
//

Motif  33 width=50 seqs=5
Cbx2_Pt                   (  286) LGLDLKVRTQKGELGMSPPGSKIPKAPSGGAVEQKVGNTGGPPHTHGASR  1
Cbx2_Hs                   (  286) LGLDLKVRTQKGELGMSPPGSKIPKAPSGGAVEQKVGNTGGPPHTHGASR  1
Cbx2_Mmu                  (  307) LGLDLKVRTQKGELGMSPPGSKIPKAPSSGAVEQKVGSTGGPTHTHGASR  1
Cbx2_Cf                   (  249) LGLDLKVRTQKGELGLSSPGSKVPKAPSSGAVEQKVGGAGGPPYIHSISK  1
Cbx2_Md                   (  284) LGLDLKVRNQKGDLGLAMPGARAMTAPNSGGSEQKSGGTGRTPNLHNGGK  1
//

Motif  34 width=41 seqs=4
Cbx4_Cf                   (  133) LQYQGGHKEAPSPTCPDLGAKSHPPDKWAHGAGAKGYLGAV  1
Cbx4_Mm                   (  171) LQYQGGHKEAPSPTCPDLGTKSHPPDKWAHGAAAKGYLGAV  1
Cbx4_Hs                   (  171) LQYQGGHKEAPSPTCPDLGAKSHPPDKWAQGAGAKGYLGAV  1
Cbx4_Rn                   (  171) LQYQGSHKEAPSPTCPDLGAKSHPPDKWAHGAAAKGYLGAV  1
//
```

```
Motif  35 width=21 seqs=20
Cbx6_Cf                   (  269) PYDARSSSSSGCPSPAPQSSS  1
Cbx6_Hs                   (  267) PYDARSSGSSGCPSPTPQSSD  1
Cbx6_Mmu                  (  274) PYDARSSGSSGCPSPAPQSSD  1
Cbx6_Mm                   (  268) PFDAHSSSSSGCPSPTLQSSD  1
Cbx6_Rn                   (  268) PFDAHSSSSSGCPSPTLQSSD  1
Cbx6_Gg                   (  194) PYDAPSSSSSGCPSPAPHSSS  1
Cbx6_Bt                   (  269) PYDERSSSSSDCPSPVPHSSE  1
Cbx4_Rn                   (  382) RLTARSISTPTCLGGSPVSEH  1
Cbx4_Mm                   (  422) RLTARSISTPTCLGGSPVSEH  1
Cbx4_Cf                   (  385) RLTARSISTPTCLGGSPAAEH  1
Cbx4_Hs                   (  427) RLTARSISTPTCLGGSPAAER  1
Cbx8_Ec                   (  131) PPGSSSSTSSTCRVEPPRDRD  1
Cbx8_Cf                   (  131) PPGSSSSTSSTCRVEPPRDRD  1
Cbx8_Hs                   (  131) PPASSTSTSSTCRAEAPRDRD  1
Cbx6_Fr                   (  280) TRDTDSASTQYQIPPSPSSSC  1
Cbx6_Tn                   (  293) PRDTRSTPTQYQTPPSPSSSS  1
Cbx2_Xl                   (   96) SSSSSSSSSSSSSSSSSSSSD  1
Cbx2_Dr                   (   94) SSSSSSSSSSGSSSSSSSSSS  1
Cbx2_Fr                   (  100) SSSSSSDSSSSCSSSSSSEDD  1
Cbx2_Gg                   (  182) PNAMKSGSSSPSRAISWQSSI  1
//

Motif  36 width=50 seqs=4
Pc_Dsi                    (  229) IQQQPFQDQQQAEKIASEAATPLKSEQQATPLATEAINTTPAESGAEEEE  1
Pc_Dse                    (  245) IQQQPFQDQQQAEKIASEAATPLKSEQQATPLATEAINTTPAESGAEEEE  1
Pc_Dy                     (  244) IQQQPFQDQQQAEKIASEAATPLKSELQATPLATEVINTTPAESGAEEEE  1
Pc_Dm                     (  245) SQQQPFQDQQQAEKIASEAATQLKSEQQATPLATEAINTTPAESGAEEEE  1
//

Motif  37 width=15 seqs=10
Cbx6_Mmu                  (  357) SSEPEAGDWRPEMSP  1
Cbx6_Hs                   (  350) SSEPEAGDWRPEMSP  1
Cbx6_Mm                   (  352) SSEPEAGDWRPEMSP  1
Cbx6_Rn                   (  352) SSEPEAGDWRPEMSP  1
Cbx6_Cf                   (  353) SSEPEAGDWRPEMSP  1
Cbx6_Bt                   (  352) ASEPEAGDWRPEMSP  1
Cbx6_Gg                   (  275) DPELEAGDWRPEMSP  1
Cbx6_Xl                   (  314) DVDEEAGDWRPDMSP  1
Cbx6_Xt                   (  313) SDVDETGDWRPDMSP  1
Cbx6_Dr                   (  374) SEEEEDLDWRPDMTS  1
//
```

```
Motif  38 width=15 seqs=10
Pc_Dy                    (  217) SGKIGVTIKTSPDGP  1
Pc_Dps                   (  218) SGKIGVTIKTSPDGP  1
Pc_Dpe                   (  218) SGKIGVTIKTSPDGP  1
Pc_Dm                    (  218) SGKIGVTIKTSPDGP  1
Pc_Dse                   (  218) SGKIGVTIKTSPDGP  1
Pc_Am                    (  216) SGKIGVTITTSPSGS  1
Pc_Aa                    (  277) EGKVGVTIKTSPDES  1
Pc_Ag                    (  217) EGKVGVTIKTSPDES  1
Pc_Tc                    (  174) SGKIGVTITTSSPTS  1
Pc_Nvi                   (  319) SGKIGVTITTSSPSS  1
//

Motif  39 width=20 seqs=10
Cbx4_Rn                  (  314) EAFGEQPLQLTTKPDLLAWD  1
Cbx4_Mm                  (  354) EAFGEQPLQLTTKPDLLAWD  1
Cbx4_Hs                  (  353) EAFGEQPLQLTTKPDLLAWD  1
Cbx4_Cf                  (  316) EAFGEQPLQLTTKPDLLAWD  1
Cbx4_Gg                  (  346) LPVAEQPLQLTTKPDLVPWS  1
Cbx4_Fr2                 (  325) QLPADQPLQLTTKSNLLSVT  1
Cbx4_Xl                  (  357) ETLNDQPLQLTTKSSHVPMP  1
Cbx4_Xt                  (  343) ESIHDQPLQLTSKSNHIPLS  1
Cbx4_Tn                  (  367) DLPDDLPLQLTASSPVTSWA  1
Cbx4_Fr1                 (  411) DLPDDLPLQLTASSPVTSWA  1
//

Motif  40 width=21 seqs=13
Cbx2_Mmu                 (  467) PGEARKAATLPEMSAGEESSS  1
Cbx2_Pt                  (  446) PGEARKAATLPEMSAGEESSS  1
Cbx2_Hs                  (  446) PGEARKAATLPEMSAGEESSS  1
Cbx2_Bt                  (  445) SGEARKTAALSEMSTGEENSS  1
Cbx2_Mm                  (  433) PGEGRKPPALSELSTGEENSS  1
Cbx2_Rn                  (  437) PGEGQKPPALCELSTGEENSS  1
Cbx2_Gg                  (  399) APESRKAAALSEMSTGDESSS  1
Cbx2_Cf                  (  409) PGDARKLALPSELSAGEESSS  1
Cbx2_Md                  (  441) AGETRKTTTLSEMSTGDDSST  1
Cbx2_Tn1                 (  221) RSAAKDGKKKNEMSAGEDESS  1
Cbx2_Dr                  (  425) KDPSKQSKTLSELSTGEEGSS  1
Cbx2_Fr                  (  410) RSAPKDGKKKNELSAGEDESS  1
Pc_Aa                    (   17) RACATCAHIFAEQSFTEERRS  1
//
```

```
Motif  41 width=29 seqs=6
Cbx8_Bt                  (  282) AKGQGTLDPGGPRVRHGSGTPGSVGGLYR  1
Cbx8_Cf                  (  284) ARGQGALDPGGPRVRHGSGTPGSVGGLYR  1
Cbx8_Ec                  (  292) AKGQSALDPGGPRVRHGSGTPGSVGGLYR  1
Cbx8_Rn                  (  271) AKGQGALDPGGTRVRHSSGTPGSVGSLYR  1
Cbx8_Mm                  (  267) AKGQGALDPGGARVRHSSGTPASVGSLYR  1
Cbx8_Hs                  (  294) AKGQGALDPNGTRVRHGSGPPSSGGGLYR  1
//

Motif  42 width=28 seqs=5
Cbx8_Tn                  (  114) TPRAREGLRAVVPTIFPPSTVNRGESVR  1
Cbx8_Dr2                 (  114) TPRAREGLRTVVPTIFPPSTINRGESVR  1
Cbx8_Dr1                 (  114) APRAREGLRAVVPTIFPPSTVNRGESVR  1
Cbx8_Fr                  (  112) TPRAREGLRTVVPTIFPPSAVNRGESVH  1
Cbx8_Tn2                 (  112) TPRAREGLRAVVPTLFPPSAINRGESVY  1
//

Motif  43 width=15 seqs=8
Cbx7_Mmu                 (  125) LVDKGPLVPTLPFPL  1
Cbx7_Pt                  (  160) LVDKGPLVPTLPFPL  1
Cbx7_Hs1                 (  125) LVDKGPLVPTLPFPL  1
Cbx7_Bt                  (  125) LADKGPLVPTLPFPL  1
Cbx7_Mm                  (  125) LVEKGPLVPTLPFPL  1
Cbx7_Cf                  (  124) MVDKGPMVPALPFPL  1
Cbx7_Ec                  (  225) LVDKGPLVPALPFSL  1
Cbx7_Rn                  (  125) LVEKGPLGPTLPFPL  1
//

Motif  44 width=32 seqs=4
Cbx4_Tn                  (  192) NPGWNLPLALQQKWVRDKETGCLSKVKELAVE  1
Cbx4_Fr1                 (  236) NPGWNLPLALQQKWVRDKETGCLSKVKESAVE  1
Cbx4_Dr                  (  187) NKGWNLPPALQQKWIRNKDTGCLSKVKDLSIE  1
Cbx4_Fr2                 (  148) RQGCNLPPVLQQKWVRDKDSGCLTKVKDIAME  1
//

Motif  45 width=29 seqs=4
Cbx4_Mm                  (  219) GAPGKGSEKGPPNGMTPAPKEAVTGNGIG  1
Cbx4_Cf                  (  181) GAPGKGSEKGPPNGMTPAPKEAVTGNGIG  1
Cbx4_Hs                  (  219) GAPGKGSEKGPPNGMMPAPKEAVTGNGIG  1
Cbx4_Rn                  (  219) GAPGKGSEKGPPNGLTPAPKEAVAGNGIG  1
//
```

```
Motif  46 width=29 seqs=5
Cbx8_Hs                  (  166) RERERERERERERERGTSRVDDKPSSPGD  1
Cbx8_Bt                  (  154) RDRERERERERERERGTSRADDKPSSPGD  1
Cbx8_Ec                  (  164) RERERERERERERDRGVGRADDKPSSPGD  1
Cbx8_Cf                  (  156) RERERERERQRERERGASRTDDKPSSPGD  1
Cbx8_Rn                  (  143) PPRDRDRDRDRERDRGTSRVDEKPSSPGD  1
//

Motif  47 width=15 seqs=7
Cbx6_Mmu                 (  191) GQGAGALARPKVPSR  1
Cbx6_Hs                  (  191) GQGAGALARPKVPSR  1
Cbx6_Bt                  (  191) GQGAGALARPKVPSR  1
Cbx6_Cf                  (  191) GQGAGALARPKVPSR  1
Cbx6_Mm                  (  191) AQGTGALARPKVPSR  1
Cbx6_Rn                  (  191) AQGTGALARPKVPSR  1
Cbx6_Gg                  (  117) ANGAGALARPKIPSR  1
//

Motif  48 width=40 seqs=6
Cbx4_Xt                  (  373) EPYKDTVYTNPRKRCLSEANGDRELCKKALASRSVSAPST  1
Cbx4_Xl                  (  387) EPYNDLVYTNPRKRCLSEANGNKELCKKTLTSRSVSAPGI  1
Cbx4_Tn                  (  398) EHIRIPSFPKDRKRKLSDPAEHRSVSKTYLSARSLSAPST  1
Cbx4_Fr1                 (  442) DHIRIPSFPKDRKRKLSDPVDHRSVSKAYLTSRSLSLPST  1
Cbx4_Gg                  (  376) SMGLNLSSPGARKRCLSEPHGDREPGKKRLTSRSISAPTC  1
Cbx4_Dr                  (  351) SPAKVDAKSNHLKRHLSEPSEDVRNCKQLLSFRSISAPNS  1
//

Motif  49 width=21 seqs=9
Cbx6_Mmu                 (  331) TSKRAPPEVTAAAGPAPPTAP  1
Cbx6_Hs                  (  324) TSKRAPPEVTAAAGPAPPTAP  1
Cbx6_Bt                  (  326) TNKRAPPEVPAAASQALPTVP  1
Cbx6_Cf                  (  327) TNKRAPPDVPAAVGQALTAAP  1
Cbx6_Rn                  (  325) TGQRVPPDVTAAAGQALHTVL  1
Cbx6_Mm                  (  325) TGQRVPPDVTGAADQALHTAL  1
Cbx4_Fr1                 (  523) TKKPAEPEVPLAPPPAVETAP  1
Pc_Am                    (  287) EKKRAEADVPHGPPPSLPRAP  1
Cbx4_Tn                  (  479) TKKPAEPEVPPVAEPPVKAAQ  1
//
```

```
Motif  50 width=29 seqs=7
Cbx4_Rn                  (  278) EEKKAEVPCKRREEEALVSGDPQPQDLGS  1
Cbx4_Mm                  (  318) EEKKAEVPCKRREEEALVSGDAQPQDLGS  1
Cbx4_Hs                  (  317) EEKKVEAPPKRREEEVSGVSDPQPQDAGS  1
Cbx4_Cf                  (  279) EEKKAEAPAKRREEEAPGPGDPQPQDAAS  1
Cbx4_Xl                  (  314) QEEKTEHWKKRVESRVKIHEGSKSVDKGS  1
Cbx4_Xt                  (  300) QEEKTEHWKKRVESRVKINESNGSVDRGS  1
Cbx4_Gg                  (  296) GEEKLEAWRKPGEERAVGSNGLSAAEGES  1
//

Motif  51 width=11 seqs=6
Cbx2_Gg                  (  203) VHYMNRMSQNQ  1
Cbx2_Mmu                 (  280) VHYMNRMTQSQ  1
Cbx2_Mm                  (  257) VHYMNRMSQSQ  1
Cbx2_Xl                  (  247) VHYMNRINQNS  1
Pc_Aa                    (  368) VSNNNTINQHQ  1
Pc_Ag                    (  308) VSNNNTINQHQ  1
//

Motif  52 width=15 seqs=6
Pc_Dsi                   (    1) MTGRGKGSKGKLGRD  1
Pc_Dm                    (    1) MTGRGKGSKGKLGRD  1
Pc_Dse                   (    1) MTGRGKGSKGKLGRD  1
Pc_Dy                    (    1) MTGRGKGGKGKLGRD  1
Pc_Dps                   (    1) MTGRGKGAKGKVVRE  1
Pc_Dpe                   (    1) MTGRGKGAKGKVVRE  1
//

Motif  53 width=29 seqs=4
Cbx6_Fr                  (  247) KMLGFPMYGKPFGIQYGGPLAFHSSPATC  1
Cbx6_Tn                  (  260) KMLGFPMYGKPFGLQYGGPLSFHSSPATC  1
Cbx4_Tn                  (  311) KIVEYPENGIPKEMCSGRTLPVAEHPMKC  1
Cbx4_Fr1                 (  355) KIVEYLENGIPKELCSARTLPVAEHPMKC  1
//

Motif  54 width=15 seqs=7
Cbx8_Ec                  (  115) ASTSRAREGLRNMGL  1
Cbx8_Hs                  (  115) ASTSRAREGLRNMGL  1
Cbx8_Bt                  (  115) ASTSRSREGLRNMGL  1
Cbx8_Rn                  (  115) ASTSRAREGLRNTGL  1
Cbx8_Cf                  (  115) ASTSRARDGLRNMGL  1
Cbx8_Xt                  (  113) ELPSRSREGLRSIPT  1
Cbx8_Xl                  (  113) ELPSRSREGLRSIPT  1
```

```
//

Motif  55 width=11 seqs=6
Pc_Dy                    (  378) ERDMKGDSSPV  1
Pc_Dps                   (  362) ERDMKGDSSPV  1
Pc_Dpe                   (  362) ERDMKGDSSPV  1
Pc_Dsi                   (  363) ERDMKGDSSPV  1
Pc_Dm                    (  379) ERDMKGDSSPV  1
Pc_Dse                   (  379) ERDMKGDSSPV  1
//

Motif  56 width=15 seqs=6
Cbx6_Mm                  (  397) AAGVAGATGGGGGTG  1
Cbx6_Rn                  (  397) AAGVAGATGGGGGTG  1
Cbx6_Mmu                 (  402) AAGVAGAAGGGGSSG  1
Cbx6_Hs                  (  395) AAGVAGAAGGGGSIG  1
Cbx6_Bt                  (  397) AAGVAGAAAGGGSSG  1
Cbx6_Cf                  (  398) ATGVAGATVGGGSGG  1
//

Motif  57 width=11 seqs=5
Cbx4_Fr2                 (    1) YNTWEPEENIL  1
Cbx4_Cf                  (    1) YNTWEPEENIL  1
Cbx2_Mmu                 (   63) HNSWEPEENIL  1
Cbx2_Cf                  (    6) HNSWEPEENIL  1
Cbx7_Gg                  (   16) YSTWEPEDHIL  1
//

Motif  58 width=11 seqs=8
Cbx2_Mmu                 (  117) KLKEPDAPSKS  1
Cbx2_Cf                  (   59) KLKEPDAPSKS  1
Cbx2_Rn                  (   94) KLKEPDAPSKS  1
Cbx2_Mm                  (   94) KLKEPDAPSKS  1
Cbx2_Hs                  (   94) KLKEPDAPSKS  1
Cbx2_Md                  (   94) KIKEPDMPSKS  1
Cbx2_Bt                  (   95) KLKESDAPSKS  1
Cbx2_Pt                  (   94) KLKEPEAPSKS  1
//
```

```
Motif  59 width=35 seqs=4
Cbx2_Fr                  (  177) RRVLKTYQESSLPGTIKKPVHPASFTFMGFHRGSR  1
Cbx2_Tn1                 (  101) RRVLKTFPEPGPPGTIKKPVHPASFTFMGFHRGSG  1
Cbx2_Dr                  (  177) RKILKPISRDSDLRGIKKPLMPASFTYTGLNRTSG  1
Cbx2_Tn2                 (  164) HHHLIRPQRVELRPGMKKPLQPASFTYTGLTRTSR  1
//

Motif  60 width=11 seqs=5
Cbx7_Dr1                 (   84) IYPMDLRSAHK  1
Cbx7_Tn                  (   84) VYTMDLRSAHK  1
Cbx7_Xt                  (   84) LYSMDLRSAHK  1
Cbx7_Gg                  (   61) LYGMDLRSAHK  1
Cbx7_Dr2                 (   85) IYTMDLRSTHR  1
//

Motif  61 width=41 seqs=2
Cbx8_Xt                  (  254) DNHPHRTKHRVDFMASVCKESSVSRSRNSLGYQEQISDFYR  1
Cbx8_Xl                  (  253) DNRPHRTKHRVNFMASMCKESSVSSSRRSLGYQEQISDFYR  1
//

Motif  62 width=16 seqs=3
Cbx6_Xt                  (  217) QIRHLKYGAFSLYNNP  1
Cbx6_Xl                  (  217) QIRHLKYGAFSLYNNP  1
Cbx6_Gg                  (  149) QIRHMKFGGFSLYNKP  1
//

Motif  63 width=50 seqs=2
Cbx8_Xl                  (  130) QGSSSTRGDIFRERLGRTEIRGEHYPMKIKRKKKHRHDKGHCEAGTQAIR  1
Cbx8_Xt                  (  130) QGSSATRGDIFSERLGRTEMRGEHYPMKIKRKKKHHSDKGLCEVGNQAIR  1
//

Motif  64 width=50 seqs=4
Pc_Aa                    (  218) VSNEPTGTSSGGKYPSPGLKISISGQNNDNDTASNSSDDQPLSHKDLAGT  1
Pc_Ag                    (  158) VSNEPTGTSSGGKYPSPGLKISISGQNNDNDTASNSSDDQPLSHKDLAGT  1
Pc_Nvi                   (  475) KSPSPTDSYTNNNRLNAGILNGHHSHNNNNNNNNNLNNNITNNNNNINNN  1
Pc_Tc                    (  299) VSKEEPEKECSAGDKSGDVKVAVNGHNNNNMTDELPVLTSPGSEYWLARN  1
//

Motif  65 width=14 seqs=4
Cbx6_Tn                  (  233) GRQNIPSRNRIIGK  1
Cbx6_Fr                  (  220) GRQNIPSRNRIIGK  1
Cbx6_Dr                  (  227) GRQKIPSRNRVIGK  1
Cbx6_Xt                  (  192) GRPKIPSRNRVIGK  1
```

```
//

Motif  66 width=17 seqs=6
Cbx6_Rn                  (  235) YKPPPAPLVPSTAGKAD  1
Cbx6_Mm                  (  235) YKPPPAPLAPSTAGKAD  1
Cbx6_Cf                  (  236) NKPPPGPLPPPPAGKAD  1
Cbx6_Bt                  (  236) NKPPPGPLQPPPAGKTD  1
Cbx6_Hs                  (  235) YKPPPAPLVAPSPGKAE  1
Cbx6_Mmu                 (  242) LAAPPAPLAAPPAGKAD  1
//

Motif  67 width=11 seqs=6
Cbx2_Mmu                 (  391) LLARHATATKG  1
Cbx2_Pt                  (  370) LLARHATATKG  1
Cbx2_Hs                  (  370) LLARHATATKG  1
Cbx2_Cf                  (  333) MVARHATASKG  1
Cbx2_Rn                  (  361) LPARHATATKA  1
Cbx2_Bt                  (  370) MVVRHAPATKG  1
//

Motif  68 width=50 seqs=2
Pc_Dps                   (  233) TPKHPPQIQSHDQQAQKTTTDSSASLKSEHQSTPLGTEVASTPAESGAEE  1
Pc_Dpe                   (  233) TPKHPPQIQSHDQQAQKTTTDSSASLKSEHQSTPLGTEVASTPAESGAEE  1
//

Motif  69 width=21 seqs=3
Cbx4_Cf                  (  254) PSHKKRAAEERHPPADRTFKK  1
Cbx4_Hs                  (  292) PSHKKRAADERHPPADRTFKK  1
Cbx4_Mm                  (  292) PSHKKRAAEERHPQGDRTFKK  1
//

Motif  70 width=11 seqs=4
Pc_Dsi                   (  217) TIKPQPTQQLT  1
Pc_Dse                   (  233) TIKPQPTQQLT  1
Pc_Dm                    (  233) TIKPQPTQQVT  1
Pc_Dy                    (  232) TIKSQPTQQLT  1
//

Motif  71 width=49 seqs=2
Cbx4_Xl                  (  168) QYTIEKESQISTDVRNRHRDSLTHTQAADMGTQLKNGTDSVISGPERNG  1
Cbx4_Xt                  (  170) QYALGKESQISTDPSNRHRESWAHTKAADVGTQMKNGTDPVLSGLERNG  1
//
```

```
Motif  72 width=47 seqs=2
Pc_Aa                    (  304) MVTPLSTGPKTDIKPAAPLSPDTPASRPESNIPLVDKSAAAGVANNV  1
Pc_Ag                    (  244) MVTPLSTGPKTDIKPAAPLSPDTPASRPESNIPLVDKSAAAGVANNV  1
//

Motif  73 width=29 seqs=2
Cbx8_Fr                  (  197) HPHREAPDHGLIQLTRRFQEETTITPKPC  1
Cbx8_Tn2                 (  198) HPRREAPDHGLIQLTRRFQEETTITPKPY  1
//

Motif  74 width=29 seqs=2
Cbx4_Fr1                 (  488) DTPMDLHCSGRRHSSACETVDSGGQEEPM  1
Cbx4_Tn                  (  444) DTPMDLHCRGRRHSSTHEAADSGGQEEPM  1
//

Motif  75 width=20 seqs=5
Cbx2_Tn1                 (   65) RDHPVPQKKAQIVMAKPEPP  1
Cbx2_Fr                  (  136) RDPPVPQKKAQIVMAKQEPP  1
Cbx2_Dr                  (  136) EHLPVPQKKAQIVVAKPGPP  1
Cbx2_Tn2                 (  118) RVQPGPQKRPQILLARPDPP  1
Cbx2_Xl                  (  131) DSHPVPQKKAPAVLARTELK  1
//

Motif  76 width=50 seqs=2
Pc_Aa                    (  133) IEDSDEDEVPVPAAPVPTTSTTPVVVTPEPVPEPEKPIKETIKKEKEEKH  1
Pc_Ag                    (   73) IEDSDEDEVPVPAAPVPTTSTTPVVVTPEPVPEPEKPIKETIKKEKEEKH  1
//

Motif  77 width=43 seqs=2
Pc_Am                    (   98) GGDVTTRRPRRRDTRYSEHVLANMVVEEEPGGDERVGEDSQDE  1
Pc_Nvi                   (  193) GGDVTARRPRRRDTRYSEQILANLVVEEEPGGDERVGEDSQDE  1
//

Motif  78 width=12 seqs=4
Cbx2_Bt                  (  287) LGLDLKMRTQKG  1
Cbx2_Mm                  (  286) LGLDLKVRTQKG  1
Cbx2_Gg                  (  236) LGLDLKMRNQKG  1
Cbx2_Rn                  (  290) LGPDLKVRTQKG  1
//

Motif  79 width=29 seqs=2
Cbx4_Xl                  (  283) IKSSEEDCDMGDVRRRFDSPGTLNGDKTC  1
Cbx4_Xt                  (  269) IKSSEEDCEMGDPRRRLESPNSFNGDKTC  1
```

```
//

Motif  80 width=26 seqs=2
Cbx6_Fr                  (  361) DGEKECVLDCSPPPQECKKNPHQFRA  1
Cbx6_Tn                  (  368) DGEKDCALDSSSSSQECRKNSHQFRA  1
//

Motif  81 width=11 seqs=4
Cbx4_Rn                  (  107) LELGTQGKGQG  1
Cbx4_Mm                  (  107) LELGTQGKGQG  1
Cbx4_Cf                  (   69) LELGGQGKGQG  1
Cbx4_Hs                  (  107) LDLGAQGKGQG  1
//

Motif  82 width=11 seqs=2
Pc_Nvi                   (  552) GTDYWHARNPV  1
Pc_Am                    (  380) GTDYWHARNPV  1
//

Motif  83 width=21 seqs=2
Cbx4_Tn                  (  345) HFSKPSPSTAEEYNTEVARGQ  1
Cbx4_Fr1                 (  389) HFSKPSPSTAEEYNTEVARGQ  1
//

Motif  84 width=29 seqs=2
Cbx6_Xl                  (  233) ALCGPERAKDGDEATQEGLADDSSSTSGC  1
Cbx6_Xt                  (  233) ALQDPGAAKDGDEATQEGLADDSSSTSGC  1
//

Motif  85 width=38 seqs=2
Cbx7_Gg                  (  110) LPFSLRKQRKNQKYLRLSRKKFPRMASLENRNCRHEFF  1
Cbx7_Xt                  (  129) LQFPLSKQRKGRKFLCLSRKKFLRVSTYEPLSRRSDFF  1
//

Motif  86 width=15 seqs=6
Cbx6_Cf                  (  254) AASPGPGLLLAAPAA  1
Cbx6_Hs                  (  252) ASAPGPGLLLAAPAA  1
Cbx6_Mm                  (  252) VASSGPGLLLATPAA  1
Cbx6_Rn                  (  252) VASSGPGLLLATPAA  1
Cbx6_Mmu                 (  259) ASAPGPGLLLAAPAT  1
Cbx6_Bt                  (  254) TASPGQGLLLAPPSA  1
//
```

```
Motif  87 width=29 seqs=2
Pc_Am                    (  251) YHSHKVNGRRPSSSSVKSTPEEPLPAMPT  1
Pc_Nvi                   (  353) YHTHKVNGRRPSSSSVKSTPEEPLPAVPT  1
//

Motif  88 width=11 seqs=4
Cbx2_Fr                  (  444) QNQDWKPMRSL  1
Cbx2_Tn1                 (  254) QNQGWKPARSL  1
Cbx2_Xl                  (  428) ASQDWKPARSL  1
Cbx2_Tn2                 (  464) AETDWRPARSL  1
//

Motif  89 width=28 seqs=2
Cbx8_Tn2                 (  155) HLPRKRGRKPKQSFDDEEDDGGASAEPY  1
Cbx8_Fr                  (  154) HLPKKRGRTPKQSFPYDEDDGGTSAEPY  1
//

Motif  90 width=9 seqs=2
Cbx7_Gg                  (   76) LCFSLARRF  1
Cbx7_Xt                  (  100) LCFSLSRRF  1
//

Motif  91 width=21 seqs=2
Cbx4_Tn                  (  285) AKHGETSSDHKPQTTKPPENN  1
Cbx4_Fr1                 (  329) AKHGETSSDMKAQSTKPPENN  1
//

Motif  92 width=8 seqs=2
Pc_Nve1                  (  294) YKDFQFRH  1
Pc_Nvi                   (   35) YDDLDFAH  1
//

Motif  93 width=13 seqs=2
Pc_Am                    (  157) DEDTLPSSLDGHE  1
Pc_Nvi                   (  261) DEDTLASSLDGHE  1
//

Motif  94 width=10 seqs=2
Pc_Ag                    (    6) DRVYAAERIM  1
Pc_Tc                    (    5) DHVYAAERII  1
//
```

```
Motif  95 width=8 seqs=2
Pc_Pc                    (  183) KEINCECW  1
Cbx7_Dr1                 (  136) KNRQCRSR  1
//

Motif  96 width=8 seqs=2
Cbx8_Tn2                 (  233) ADHDPAWT  1
Cbx8_Fr                  (  286) ADHDPSWT  1
//

Motif  97 width=8 seqs=2
Cbx7_Dr1                 (  223) CSSIFDQE  1
Pc_Nve1                  (  192) CAETQTQQ  1
//

Motif  98 width=8 seqs=2
Cbx8_Dr2                 (  211) NCDIMHFT  1
Cbx2_Mmu                 (   25) GSTLACCR  1
//

Motif  99 width=9 seqs=2
Cbx7_Dr2                 (  118) CIFQQLNHH  1
Cbx8_Fr                  (  229) CVRGAGLRY  1
//

Motif  100 width=9 seqs=2
Cbx7_Dr1                 (  264) DEDCVWVSH  1
Cbx2_Dr                  (  117) DDDDEDDHN  1
//
```
