# Supplementary material for: Novel motifs distinguish multiple homologues of Polycomb in vertebrates: expansion and diversification of the epigenetic toolkit
Source: BMC Genomics. 2009 Nov 20;10:549. doi: 10.1186/1471-2164-10-549 (PMC2784810; doi:10.1186/1471-2164-10-549)
Supplement: Additional file 4 — The motif alignment of PC homologues. The motifs predicted by MEME tool are aligned to the homologues. [file 1471-2164-10-549-S4.PDF]

Additional file 4 - The motif alignment of PC homologues

The motifs predicted by MEME tool are aligned to the homologues. Sequences were grouped based on their motif conservation and given the name of their species. The length of protein and its e-value is shown. Each motif is represented with a number and the details about the motifs are given in the Additional file 3.

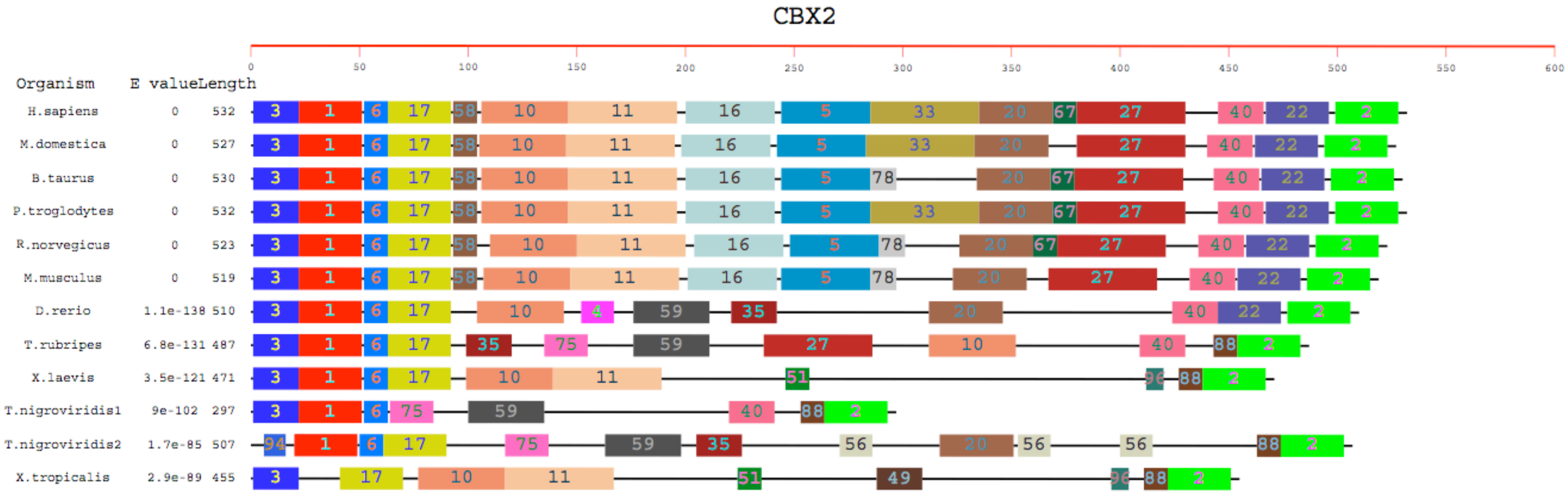

CBX4

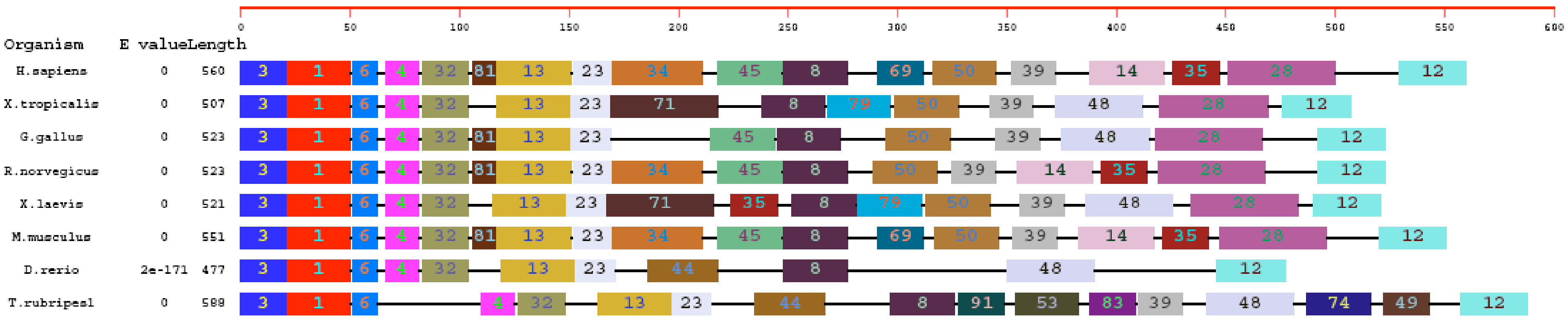
0 50 100 150 200 250 300 350 400 450 500 550 600
Organism
E value
Length
H.sapiens 0 560
X.tropicalis 0 507
G.gallus 0 523
R.norvegicus 0 523
X.laevis 0 521
M.musculus 0 551
D.rerio 2e-171 477
T.rubripesl 0 589

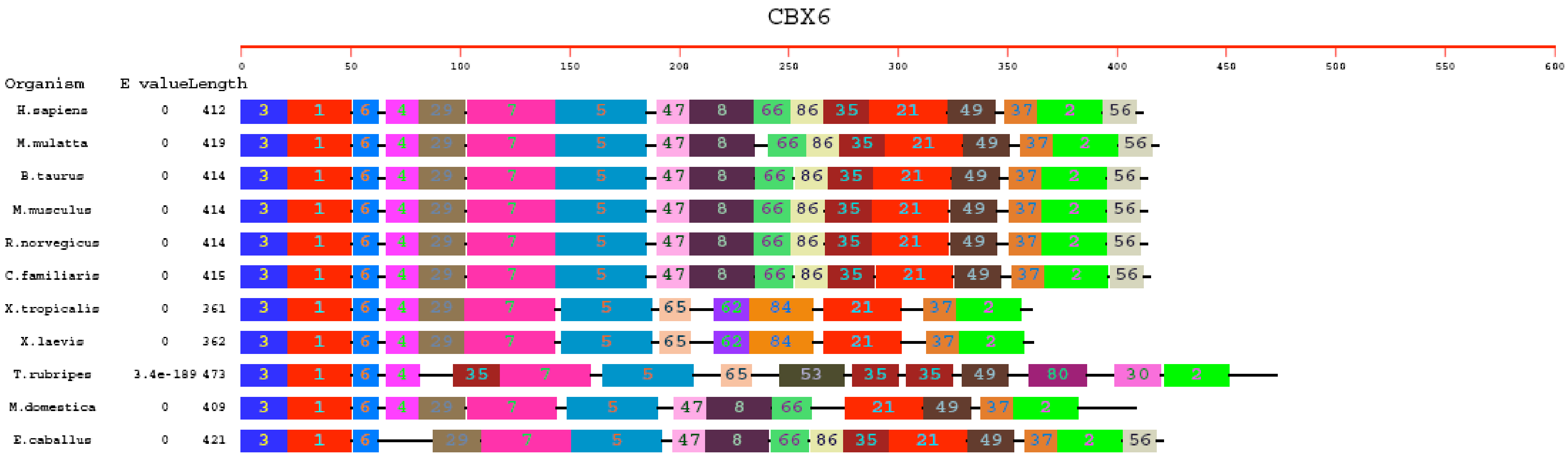

CBX6
0 50 100 150 200 250 300 350 400 450 500 550 600
Organism E value Length
H.sapiens 0 412
M.mulatta 0 419
B.taurus 0 414
M.musculus 0 414
R.norvegicus 0 414
C.familiaris 0 415
X.tropicalis 0 361
X.laevis 0 362
T.rubripes 3.4e-189 473
M.domestica 0 409
E.caballus 0 421

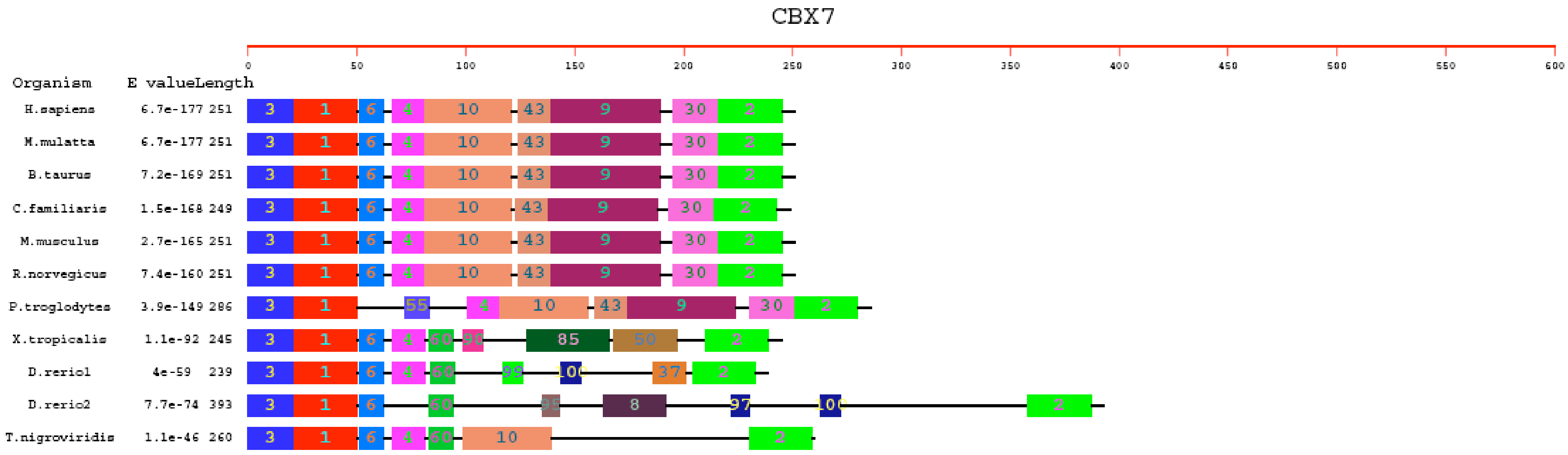
CBX7
0 50 100 150 200 250 300 350 400 450 500 550 600
Organism
E value
Length
H.sapiens 6.7e-177 251
M.mulatta 6.7e-177 251
B.taurus 7.2e-169 251
C.familiaris 1.5e-168 249
M.musculus 2.7e-165 251
R.norvegicus 7.4e-160 251
P.troglodytes 3.9e-149 286
X.tropicalis 1.1e-92 245
D.rerio1 4e-59 239
D.rerio2 7.7e-74 393
T.nigroviridis 1.1e-46 260

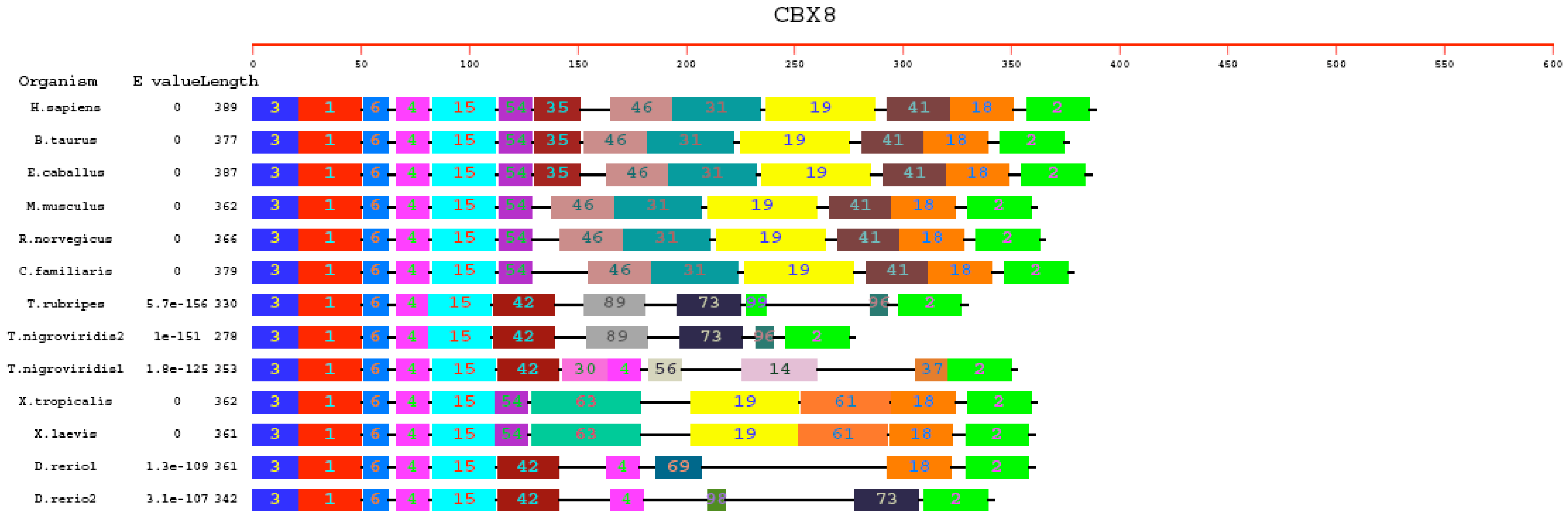
CBX8
0
50
100
150
200
250
300
350
400
450
500
550
600
Organism
E value
Length
H.sapiens 0 389
B.taurus 0 377
E.caballus 0 387
M.musculus 0 362
R.norvegicus 0 366
C.familiaris 0 379
T.rubripes 5.7e-156 330
T.nigroviridis2 1e-151 278
T.nigroviridis1 1.8e-125 353
X.tropicalis 0 362
X.laevis 0 361
D.rerio1 1.3e-109 361
D.rerio2 3.1e-107 342

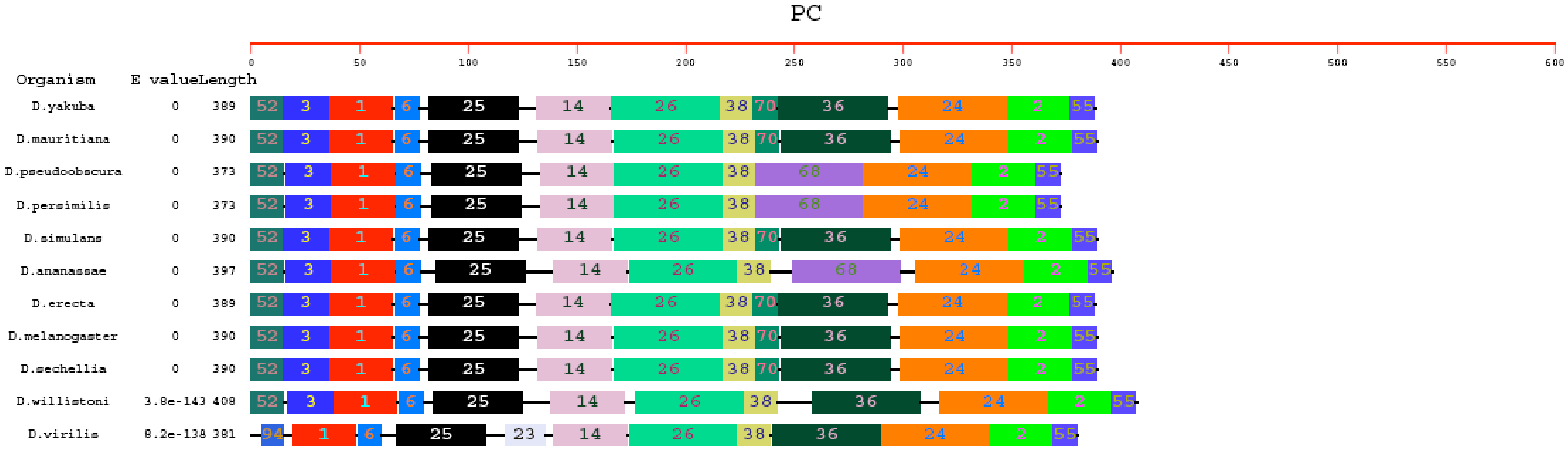
PC
0 50 100 150 200 250 300 350 400 450 500 550 600
Organism E value Length
D.yakuba 0 389
52 3 1 6 25 14 26 38 70 36 24 2 55
D.mauritiana 0 390
52 3 1 6 25 14 26 38 70 36 24 2 55
D.pseudoobscura 0 373
52 3 1 6 25 14 26 38 68 24 2 55
D.persimilis 0 373
52 3 1 6 25 14 26 38 68 24 2 55
D.simulans 0 390
52 3 1 6 25 14 26 38 70 36 24 2 55
D.ananassae 0 397
52 3 1 6 25 14 26 38 68 24 2 55
D.erecta 0 389
52 3 1 6 25 14 26 38 70 36 24 2 55
D.melanogaster 0 390
52 3 1 6 25 14 26 38 70 36 24 2 55
D.sechellia 0 390
52 3 1 6 25 14 26 38 70 36 24 2 55
D.willistoni 3.8e-143 408
52 3 1 6 25 14 26 38 36 24 2 55
D.virilis 8.2e-138 381
94 1 6 25 23 14 26 38 36 24 2 55
