# Supplementary material for: Novel motifs distinguish multiple homologues of Polycomb in vertebrates: expansion and diversification of the epigenetic toolkit
Source: BMC Genomics. 2009 Nov 20;10:549. doi: 10.1186/1471-2164-10-549 (PMC2784810; doi:10.1186/1471-2164-10-549)

## Additional file 5 - Conserved regions of PC homologues

The conserved motifs that are described in Figure 2 and 3 are represented in multiple sequence alignment format. The sequences are named with the protein name and species name. The degree of conservation is represented as bar graph at the bottom of the alignment. The amino acids are highlighted in different colours in clustal format based on their properties. The duplicated fish homologues are highlighted with a number following to the species name.

### AT-Hook motif

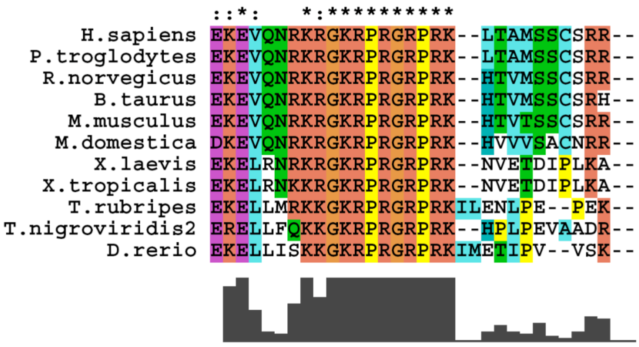

**AT-Hook Like motif**

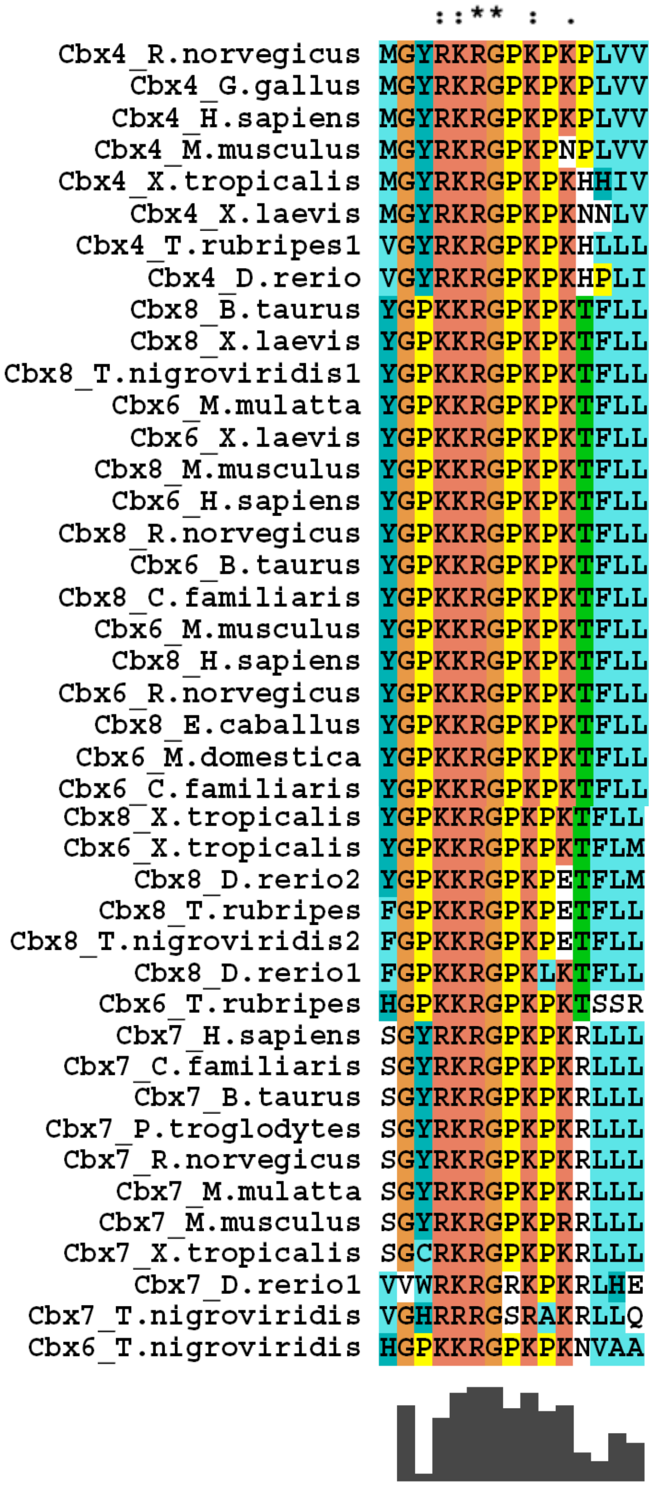

## Cx2.1

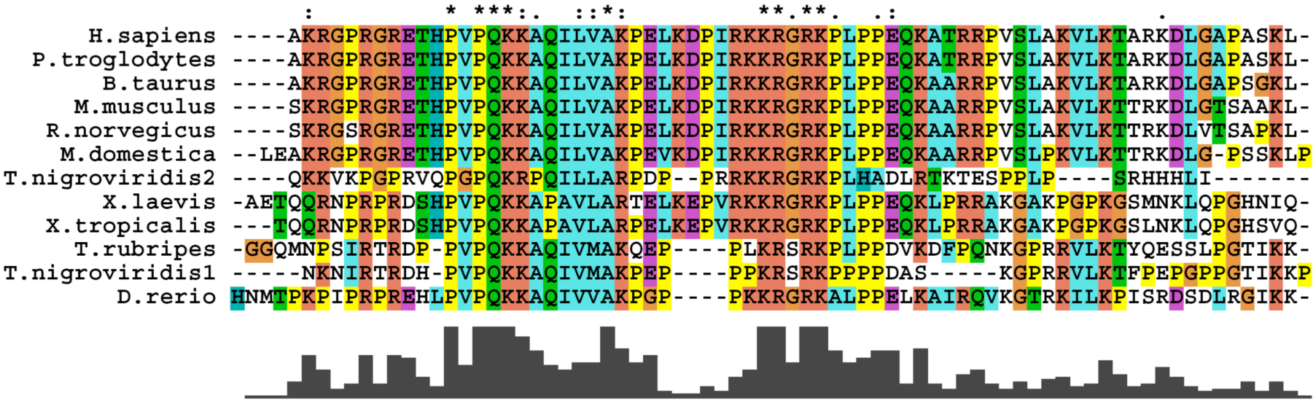

H.sapiens ----AKRGPRGRETHPVPQKKAQILVAKPELKDPIRKKRGRKPLPPEQKATRRPVSLAKVLKTARKDLGAPASKL-
P.troglodytes ----AKRGPRGRETHPVPQKKAQILVAKPELKDPIRKKRGRKPLPPEQKATRRPVSLAKVLKTARKDLGAPASKL-
B.taurus ----AKRGPRGRETHPVPQKKAQILVAKPELKDPIRKKRGRKPLPPEQKAARRPVSLAKVLKTARKDLGAPSGKL-
M.musculus ----SKRGPRGRETHPVPQKKAQILVAKPELKDPIRKKRGRKPLPPEQKAARRPVSLAKVLKTTRKDLGTSAAKL-
R.norvegicus ----SKRGSRGRETHPVPQKKAQILVAKPELKDPIRKKRGRKPLPPEQKAARRPVSLAKVLKTTRKDLVTSAPKL-
M.domestica --LEAKRGPRGRETHPVPQKKAQILVAKPEVKDPIRKKRGRKPLPPEQKAARRPVSLPKVLKTTRKDLG-PSSKLP
T.nigroviridis2 ----QKKVKPGPRVQPGPQKRPQILLARPDP--PRRKKRGRKPLHADLRTKTESPPLP----SRHHHLI-------
X.laevis -AETQQRNPRPRDSHPVPQKKAPAVLARTELKEPVRKKRGRKPLPPEQKLPRRAKGAKPGPKGSMNKLQPGHNIQ-
X.tropicalis ---TQQRNPRPRDSHPVPQKKAPAVLARPELKEPVRKKRGRKPLPPEQKLPRRAKGAKPGPKGSLNKLQPGHSVQ-
T.rubripes -GGQMNPSIRTRDP-PVPQKKAQIVMAKQEP----PLKRSRKPLPPDVKDFPQNKGPRRVLKTYQESSLPGTIKK-
T.nigroviridis1 -----NKNIRTRDH-PVPQKKAQIVMAKPEP----PPKRSRKPPPPDAS-----KGPRRVLKTFPEPGPPGTIKKP
D.rerio HNMTPKPIPRPREHLPVPQKKAQIVVAKPGP----PKKRGRKALPPELKAIRQVKGTRKILKPISRDSDLRGIKK-

**Cx2.2**

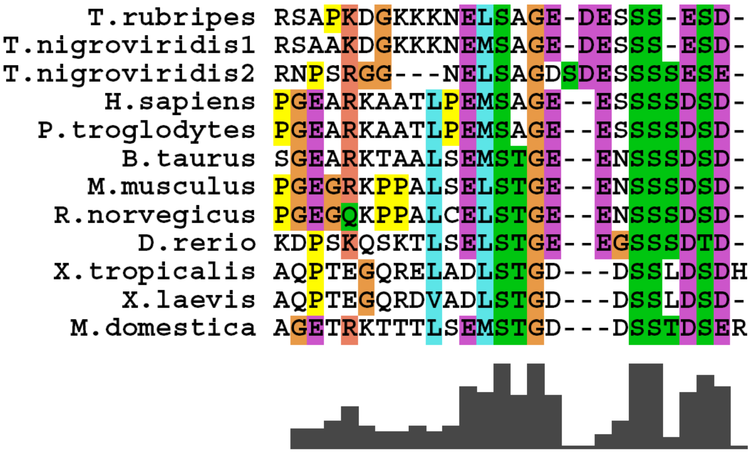
T.rubripes RSAPKDGKKKNELSAGE-DESSS-ESD-
T.nigroviridis1 RSAAKDGKKKNEMSAGE-DESSS-ESD-
T.nigroviridis2 RNPSRGG---NELSAGDSDESSSSESE-
H.sapiens PGEARKAATLPEMSAGE--ESSSSDSD-
P.troglodytes PGEARKAATLPEMSAGE--ESSSSDSD-
B.taurus SGEARKTAALSEMSTGE--ENSSSDSD-
M.musculus PGEGRKPPALSELSTGE--ENSSSDSD-
R.norvegicus PGEGQKPPALCELSTGE--ENSSSDSD-
D.rerio KDPSKQSKTLSELSTGE--EGSSSDTD-
X.tropicalis AQPTEGQRELADLSTGD---DSSLDSDH
X.laevis AQPTEGQRDVADLSTGD---DSSLDSD-
M.domestica AGETRKTTTLSEMSTGD---DSSTDSER

## Serine rich region (CBX2)

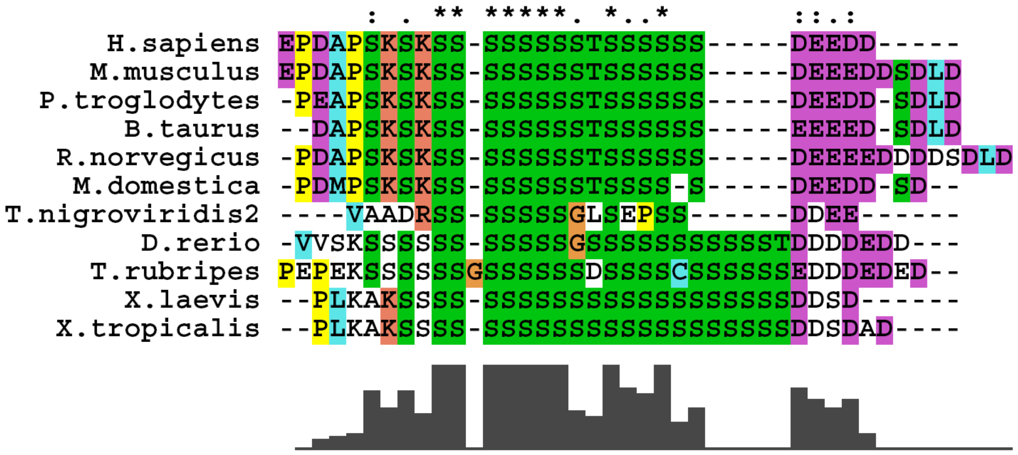

Cx4.1

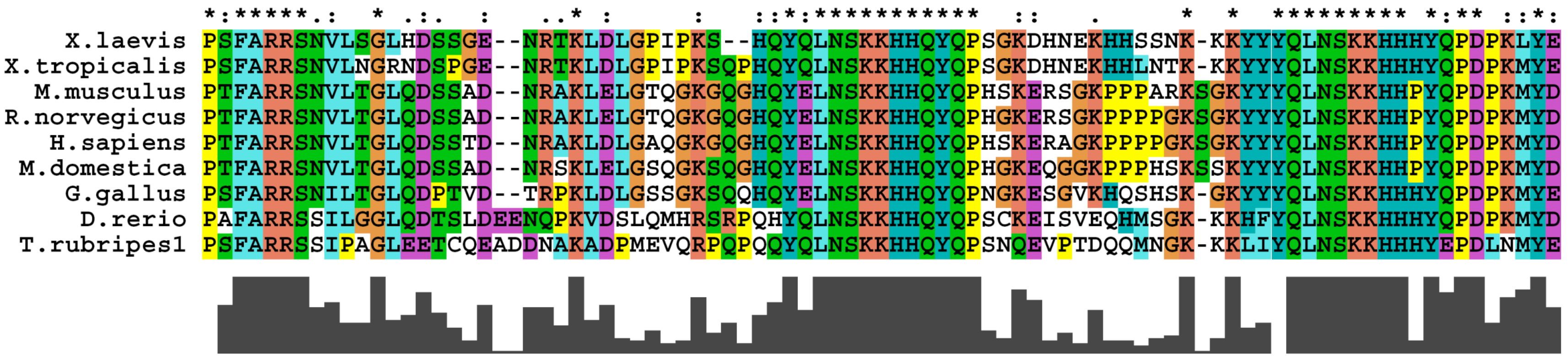
X.laevis PSFARRSNVLSGLHDSSGE--NRTKLDLGPIPKS--HQYQLNSKKHHQYQPSGKDHNEKHHSSNK-KKYYYQLNSKKHHHYQPDPKLYE
X.tropicalis PSFARRSNVLNGRNDSPGE--NRTKLDLGPIPKSQPHQYQLNSKKHHQYQPSGKDHNEKHHLNTK-KKYYYQLNSKKHHHYQPDPKMYE
M.musculus PTFARRSNVLTGLQDSSAD--NRAKLELGTQGKGQGHQYELNSKKHHQYQPHSKERSGKPPPARKSGKYYYQLNSKKHHPYQPDPKMYD
R.norvegicus PTFARRSNVLTGLQDSSAD--NRAKLELGTQGKGQGHQYELNSKKHHQYQPHGKERSGKPPPPGKSGKYYYQLNSKKHHPYQPDPKMYD
H.sapiens PTFARRSNVLTGLQDSSTD--NRAKLDLGAQGKGQGHQYELNSKKHHQYQPHSKERAGKPPPPGKSGKYYYQLNSKKHHPYQPDPKMYD
M.domestica PTFARRSNVLTGLQDSSAD--NRSKLELGSQGKSQGHQYELNSKKHHQYQPHGKEQGGKPPPHSKSSKYYYQLNSKKHHPYQPDPKMYD
G.gallus PSFARRSNILTGLQDPTVD--TRPKLDLGSSGKSQQHQYELNSKKHHQYQPNGKESGVKHQSHSK-GKYYYQLNSKKHHHYQPDPKMYE
D.rerio PAFARRSSILGGLQDTSLDEENQPKVDSLQMHRSRPQHYQLNSKKHHQYQPSCKEISVEQHMSGK-KKHFYQLNSKKHHHYQPDPKMYD
T.rubripes1 PSFARRSSIPAGLEETCQEADDNAKADPMEVQRPQPQQYQLNSKKHHQYQPSNQEVPTDQQMNGK-KKLIYQLNSKKHHHYEPDLNMYE

**Cx4.2**

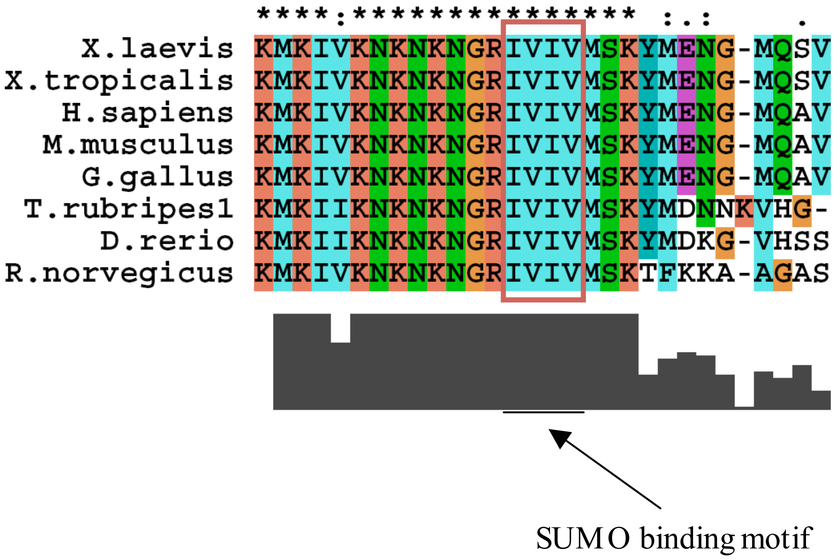
X.laevis KMKIVKNKNKNGRIVIVMSKYMENG-MQSV
X.tropicalis KMKIVKNKNKNGRIVIVMSKYMENG-MQSV
H.sapiens KMKIVKNKNKNGRIVIVMSKYMENG-MQAV
M.musculus KMKIVKNKNKNGRIVIVMSKYMENG-MQAV
G.gallus KMKIVKNKNKNGRIVIVMSKYMENG-MQAV
T.rubripes1 KMKIIKNKNKNGRIVIVMSKYMDNNKVHG-
D.rerio KMKIIKNKNKNGRIVIVMSKYMDKG-VHSS
R.norvegicus KMKIVKNKNKNGRIVIVMSKTFKKA-AGAS
SUMO binding motif

**Cx4.3**

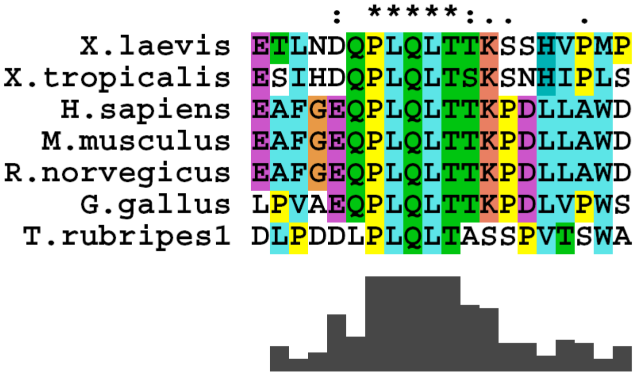

: *****:.. .
X.laevis ETLNDQPLQLTTKSSHVPMP
X.tropicalis ESIHDQPLQLTSKSNHIPLS
H.sapiens EAFGEQPLQLTTKPDLLAWD
M.musculus EAFGEQPLQLTTKPDLLAWD
R.norvegicus EAFGEQPLQLTTKPDLLAWD
G.gallus LPVAEQPLQLTTKPDLVPWS
T.rubripes1 DLPDDLPLQLTASSPVTSWA

**Cx4.4**

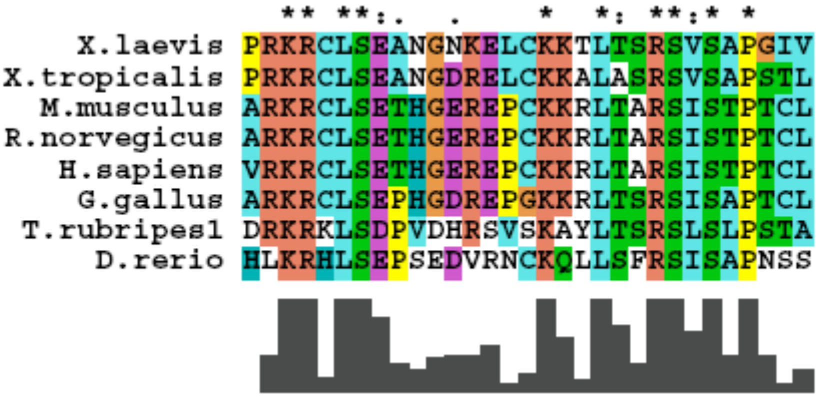
X.laevis PRKRCLSEANGNKELCKKTLTSRSVSAPGIV
X.tropicalis PRKRCLSEANGDRELCKKALASRSVSAPSTL
M.musculus ARKRCLSETHGEREPCKKRLTARSISTPTCL
R.norvegicus ARKRCLSETHGEREPCKKRLTARSISTPTCL
H.sapiens VRKRCLSETHGEREPCKKRLTARSISTPTCL
G.gallus ARKRCLSEPHGDREPGKKRLTSRSISAPTCL
T.rubripes1 DRKRKLSDPVDHRSVSKAYLTSRSLSLPSTA
D.rerio HLKRHLSEPSEDVRNCKQLLSFRSISAPNSS

Cx6.1

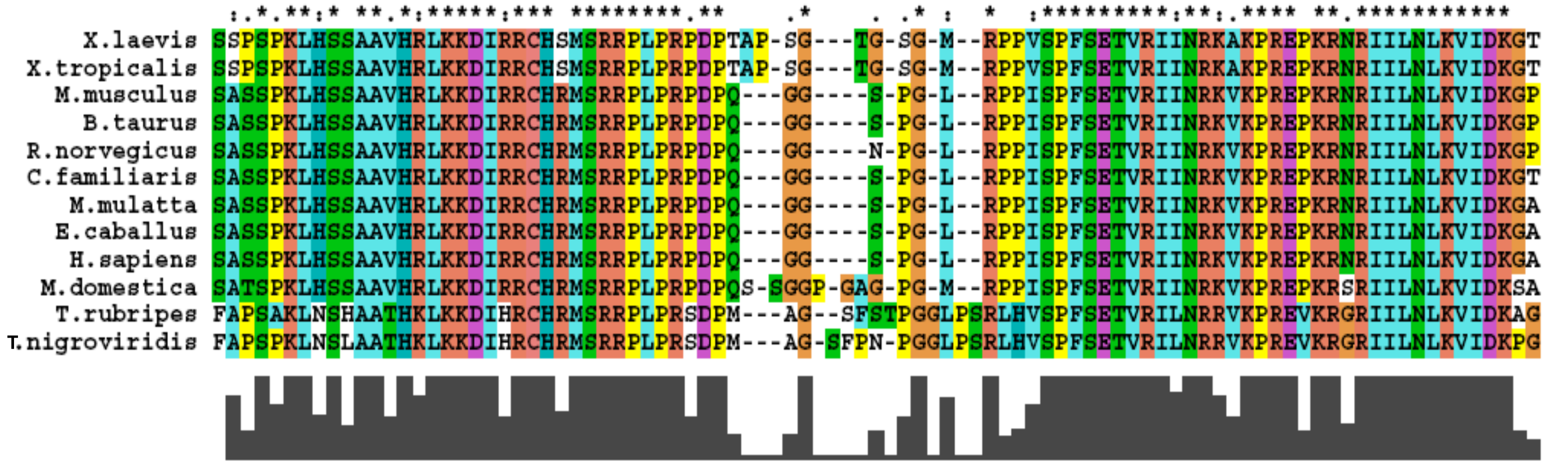

X.laevis SSPSPKLHSSAAVHRLKKDIRRCHSMSRRPLPRPDPTAP-SG---TG-SG-M--RPPVSPFSETVRIINRKAKPREPKRNRIILNLKVIDKGT
X.tropicalis SSPSPKLHSSAAVHRLKKDIRRCHSMSRRPLPRPDPTAP-SG---TG-SG-M--RPPVSPFSETVRIINRKAKPREPKRNRIILNLKVIDKGT
M.musculus SASSPKLHSSAAVHRLKKDIRRCHRMSRRPLPRPDPQ---GG----S-PG-L--RPPISPFSETVRIINRKVKPREPKRNRIILNLKVIDKGP
B.taurus SASSPKLHSSAAVHRLKKDIRRCHRMSRRPLPRPDPQ---GG----S-PG-L--RPPISPFSETVRIINRKVKPREPKRNRIILNLKVIDKGP
R.norvegicus SASSPKLHSSAAVHRLKKDIRRCHRMSRRPLPRPDPQ---GG----N-PG-L--RPPISPFSETVRIINRKVKPREPKRNRIILNLKVIDKGP
C.familiaris SASSPKLHSSAAVHRLKKDIRRCHRMSRRPLPRPDPQ---GG----S-PG-L--RPPISPFSETVRIINRKVKPREPKRNRIILNLKVIDKGT
M.mulatta SASSPKLHSSAAVHRLKKDIRRCHRMSRRPLPRPDPQ---GG----S-PG-L--RPPISPFSETVRIINRKVKPREPKRNRIILNLKVIDKGA
E.caballus SASSPKLHSSAAVHRLKKDIRRCHRMSRRPLPRPDPQ---GG----S-PG-L--RPPISPFSETVRIINRKVKPREPKRNRIILNLKVIDKGA
H.sapiens SASSPKLHSSAAVHRLKKDIRRCHRMSRRPLPRPDPQ---GG----S-PG-L--RPPISPFSETVRIINRKVKPREPKRNRIILNLKVIDKGA
M.domestica SATSPKLHSSAAVHRLKKDIRRCHRMSRRPLPRPDPQS-SGGP-GAG-PG-M--RPPISPFSETVRIINRKVKPREPKRSRIILNLKVIDKSA
T.rubripes FAPSAKLNSHAATHKLKKDIHRCHRMSRRPLPRSDPM---AG--SFSTPGGLPSRLHVSPFSETVRILNRRVKPREVKRGRIILNLKVIDKAG
T.nigroviridis FAPSPKLNSLAATHKLKKDIHRCHRMSRRPLPRSDPM---AG-SFPN-PGGLPSRLHVSPFSETVRILNRRVKPREVKRGRIILNLKVIDKPG

**Cx6.2**

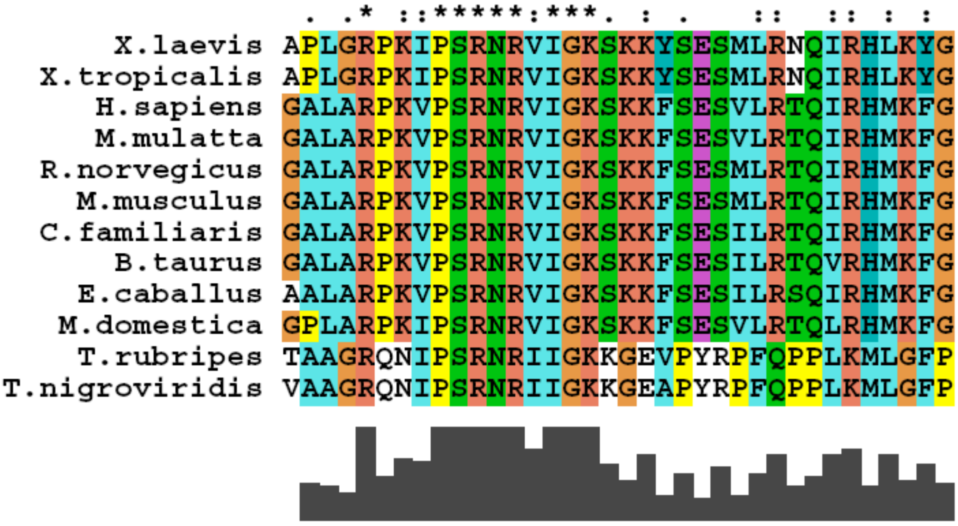
. .* ::*****:***. : . :: :: : :
X.laevis APLGRPKIPSRNRVIGKSKKYSESMLRNQIRHLKYG
X.tropicalis APLGRPKIPSRNRVIGKSKKYSESMLRNQIRHLKYG
H.sapiens GALARPKVPSRNRVIGKSKKFSESVLRTQIRHMKFG
M.mulatta GALARPKVPSRNRVIGKSKKFSESVLRTQIRHMKFG
R.norvegicus GALARPKVPSRNRVIGKSKKFSESMLRTQIRHMKFG
M.musculus GALARPKVPSRNRVIGKSKKFSESMLRTQIRHMKFG
C.familiaris GALARPKVPSRNRVIGKSKKFSESILRTQIRHMKFG
B.taurus GALARPKVPSRNRVIGKSKKFSESILRTQVRHMKFG
E.caballus AALARPKVPSRNRVIGKSKKFSESILRSQIRHMKFG
M.domestica GPLARPKIPSRNRVIGKSKKFSESVLRTQLRHMKFG
T.rubripes TAAGRQNIPSRNRIIGKKGEVPYRPFQPPLKMLGFP
T.nigroviridis VAAGRQNIPSRNRIIGKKGEAPYRPFQPPLKMLGFP

## Cx6.3

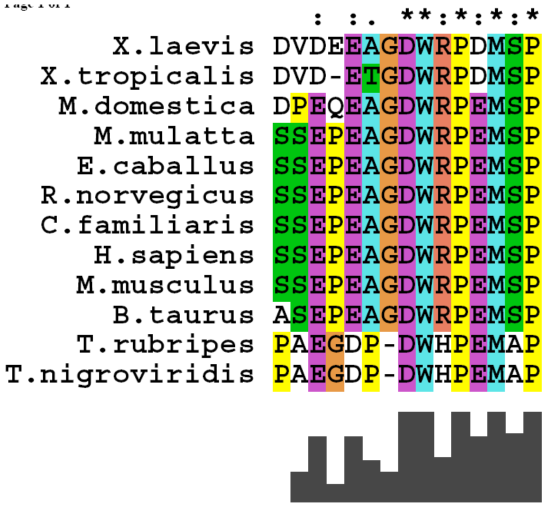
X.laevis DVDEEAGDWRPDMSP
X.tropicalis DVD-ETGDWRPDMSP
M.domestica DPEQEAGDWRPEMSP
M.mulatta SSEPEAGDWRPEMSP
E.caballus SSEPEAGDWRPEMSP
R.norvegicus SSEPEAGDWRPEMSP
C.familiaris SSEPEAGDWRPEMSP
H.sapiens SSEPEAGDWRPEMSP
M.musculus SSEPEAGDWRPEMSP
B.taurus ASEPEAGDWRPEMSP
T.rubripes PAEGDP-DWHPEMAP
T.nigroviridis PAEGDP-DWHPEMAP

**Cx8.1**

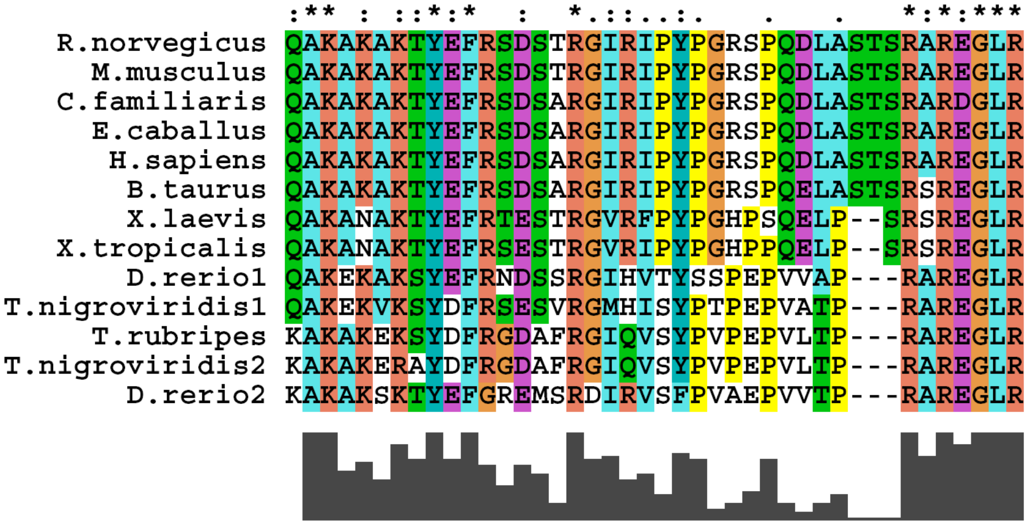
R.norvegicus QAKAKAKTYEFRSDSTRGIRIPYPGRSPQDLASTSRAREGLR
M.musculus QAKAKAKTYEFRSDSTRGIRIPYPGRSPQDLASTSRAREGLR
C.familiaris QAKAKAKTYEFRSDSARGIRIPYPGRSPQDLASTSRARDGLR
E.caballus QAKAKAKTYEFRSDSARGIRIPYPGRSPQDLASTSRAREGLR
H.sapiens QAKAKAKTYEFRSDSARGIRIPYPGRSPQDLASTSRAREGLR
B.taurus QAKAKAKTYEFRSDSARGIRIPYPGRSPQELASTSRSREGLR
X.laevis QAKANAKTYEFRTESTRGVRFPYPGHPSQELP--SRSREGLR
X.tropicalis QAKANAKTYEFRSESTRGVRIPYPGHPPQELP--SRSREGLR
D.rerio1 QAKEKAKSYEFRNDSSRGIHVTYSSPEPVVAP---RAREGLR
T.nigroviridis1 QAKEKVKSYDFRSESVRGMHISYPTPEPVATP---RAREGLR
T.rubripes KAKAKEKSYDFRGDAFRGIQVSYPVPEPVLTP---RAREGLR
T.nigroviridis2 KAKAKERAYDFRGDAFRGIQVSYPVPEPVLTP---RAREGLR
D.rerio2 KAKAKSKTYEFGREMSRDIRVSFPVAEPVVTP---RAREGLR

**RE/RED repeat (CBX8)**

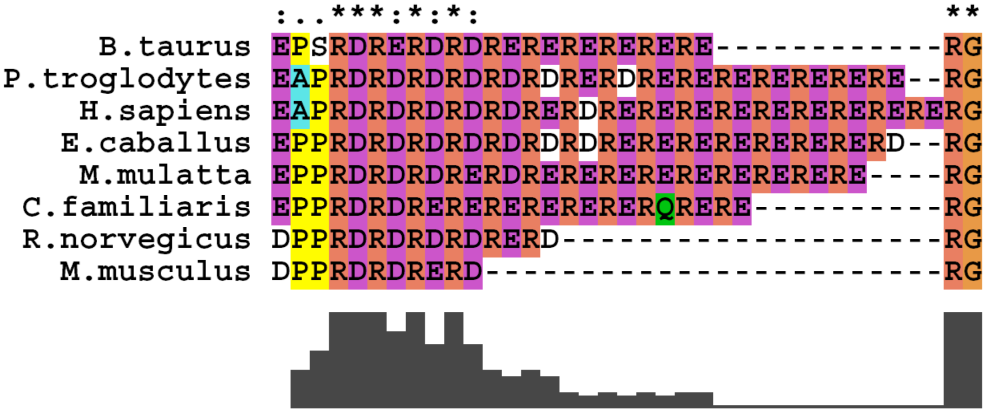

## Conserved Insect Pc (CIPC) box

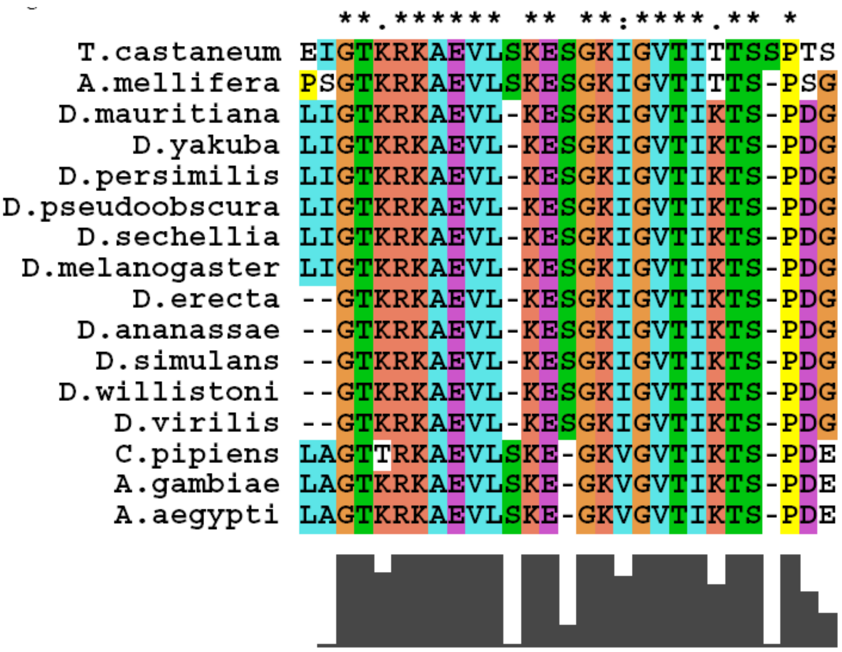

Supplement: Additional file 5 — Conserved regions of PC homologues. The conserved motifs that are described in Figures 2 and 3 are represented in multiple sequence alignment format. [file 1471-2164-10-549-S5.PDF]
