# Supplementary material for: Novel motifs distinguish multiple homologues of Polycomb in vertebrates: expansion and diversification of the epigenetic toolkit
Source: BMC Genomics. 2009 Nov 20;10:549. doi: 10.1186/1471-2164-10-549 (PMC2784810; doi:10.1186/1471-2164-10-549)
Supplement: Additional file 6 — List of AT Hook like motif containing proteins. The table describes proteins having AT Hook Like motif, ATHL, from different organisms. [file 1471-2164-10-549-S6.PDF]

Additional file 6 – The list of AT Hook Like motif containing proteins

S No	Protein	Accession id	Known domain	Organism
1	Sex comb on midleg (SCM)	Q8K214	MBT repeat, SAM domain	*Drosophila, Homo sapiens*
2	Putative transposase	BAF49631	Transposase DDE domain	*Microcystis aeruginosa*
3	SMARC5	O60264	SANT,DNA/RNA helicase, C-terminal,DEAD-like helicase	*Homo sapiens*
4	Cbx4,Cbx6,Cbx7 and Cbx8	-	Chromodomain	*Vertebrates*
5	Rapamycin-insensitive companion of mTOR	Q6R327	Ras-like GTPases, ARM repeat	*Homo sapiens*
6	Mapk15	Q80Y86	-	*Mus musculus*
7	pH-response transcription factor	Q6CQ07	Zinc finger C2H2-type 1,2,3	*Kluyveromyces lactis*
8	H3-K79-HMTase	Q6FNM5	SAM-binding motif	*Candida glabrata*
9	Putative zinc cluster protein	P43551.1	Zn(2)-C6 fungal-type	*Saccharomyces cerevisiae*
10	Putative TetR family transcriptional regulator	YP_002779987.1	Serine/Threonine protein kinase domain	*Rhodococcus opacus*
11	Holliday junction DNA helicase RuvB	ZP_03609659.1	ATPase, AAA-type	*Campylobacter rectus RM3267*
12	RNA polymerase, sigma 70 subunit	YP_002487688.1	RNA polymerase sigma-70 factor	*Arthrobacter chlorophenolicus A6*
13	Serine/threonine protein kinase with CHASE2 sensor	YP_002512778.1	Protein kinase domain	*Thioalkalivibrio sp.*
14	Similar to phd finger protein	XP_001948004.1	Zinc finger, FYVE/PHD-type	*Acyrthosiphon pisum*
15	Histone family protein nucleoid-structuring protein H-NS	ZP_02881528.1	Histone_HNS	*Burkholderia graminis C4D1M*
16	Zinc finger protein	XP_001862258.1	Zinc finger, C2H2-type	*Culex quinquefasciatus*
17	Transposase	YP_001660441.1	Transposase, IS4-like	*Microcystis aeruginosa*
18	DEAD/DEAH box helicase domain protein	YP_002431477.1	DNA/RNA helicase, DEAD/DEAH box type	*Desulfatibacillum alkenivorans*
19	RRP1-like protein/Novel nuclear protein 1	P56183	-	*Mus musculus*
20	WD-40 repeat-containing protein MSI4	O22607	WD40	*Arabidopsis thaliana*
21	Iimmediate-early protein	BAA32781.1	Herpes_ICP4	*Canid herpesvirus 1*
