# Supplementary material for: Novel motifs distinguish multiple homologues of Polycomb in vertebrates: expansion and diversification of the epigenetic toolkit
Source: BMC Genomics. 2009 Nov 20;10:549. doi: 10.1186/1471-2164-10-549 (PMC2784810; doi:10.1186/1471-2164-10-549)
Supplement: Additional file 7 — The phylogenetic tree of Chromdomain, PcR box and PC homologues. The phylogenetic tree generated using Chromodomain, PcR box and PC homologues (entire protein) represented in Figures 6, 9 and 10 are shown in a simplified form based on the consensus branching of the homologues. [file 1471-2164-10-549-S7.PDF]

Additional file 7 - The phylogenetic tree of Chromdomain, PcR box and PC homologues

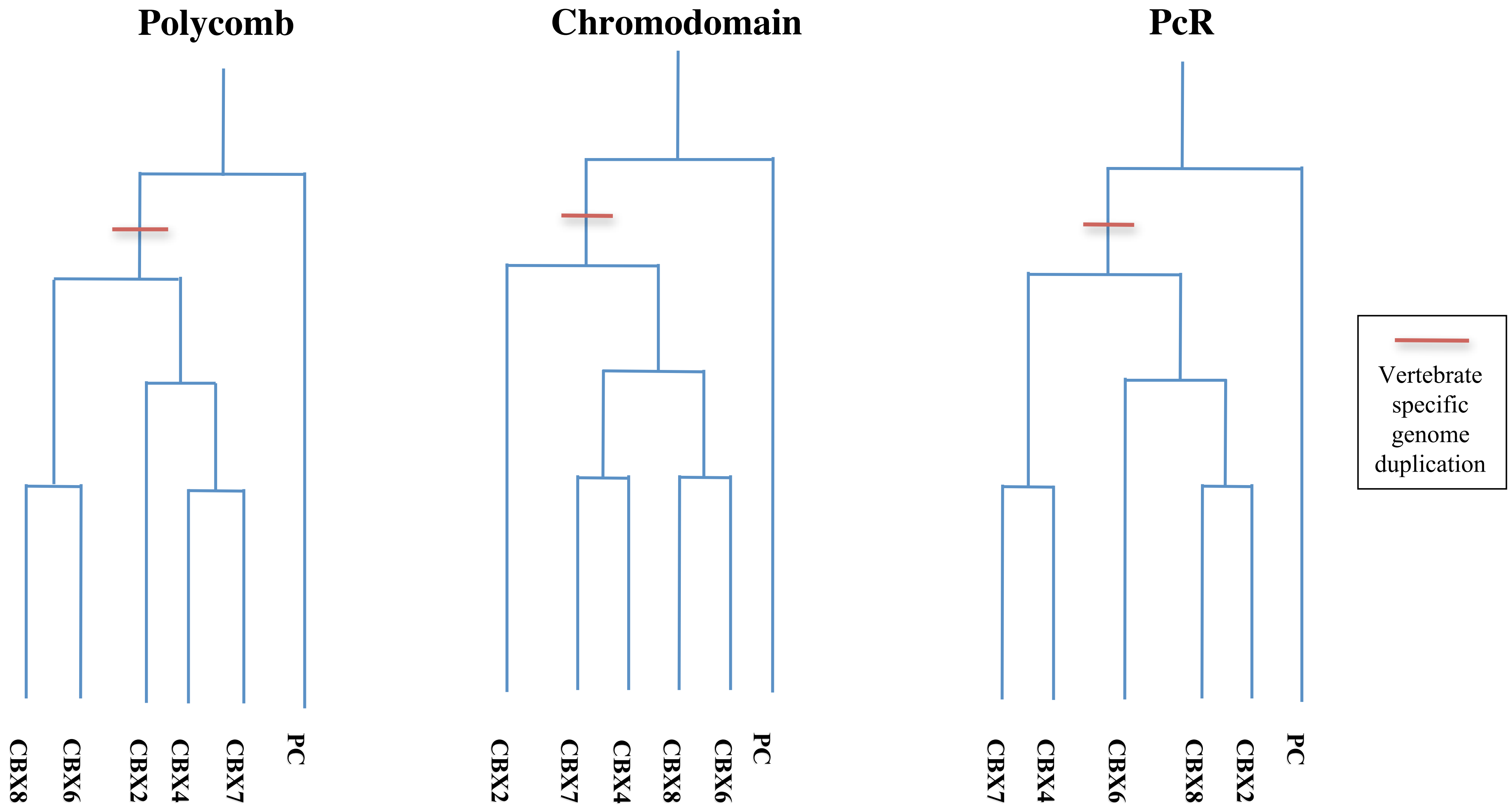

The phylogenetic tree of Chromodomain, PcR box and PC homologues represented in Figure 6, 9 and 10 are simplified based on the consensus branching of the homologues. The length of branches are not to the scale.
