# Supplementary material for: Novel motifs distinguish multiple homologues of Polycomb in vertebrates: expansion and diversification of the epigenetic toolkit
Source: BMC Genomics. 2009 Nov 20;10:549. doi: 10.1186/1471-2164-10-549 (PMC2784810; doi:10.1186/1471-2164-10-549)
Supplement: Additional file 8 — A nucleosome cartoon showing three way interaction of DNA-polycomb-H3K27Me3. AT-Hook/ATHL motif is shown to interact with DNA while adjacent chomodomain interacts with the histone H3K27Me3. This three-way interaction locks the nucleosome disallowing any remodelling or dissociation till PC (alone or as part of PRC1) is dislodged. [file 1471-2164-10-549-S8.PDF]

Additional file 8 – A nucleosome cartoon showing three way interaction of DNA-polycomb-H3K27Me3

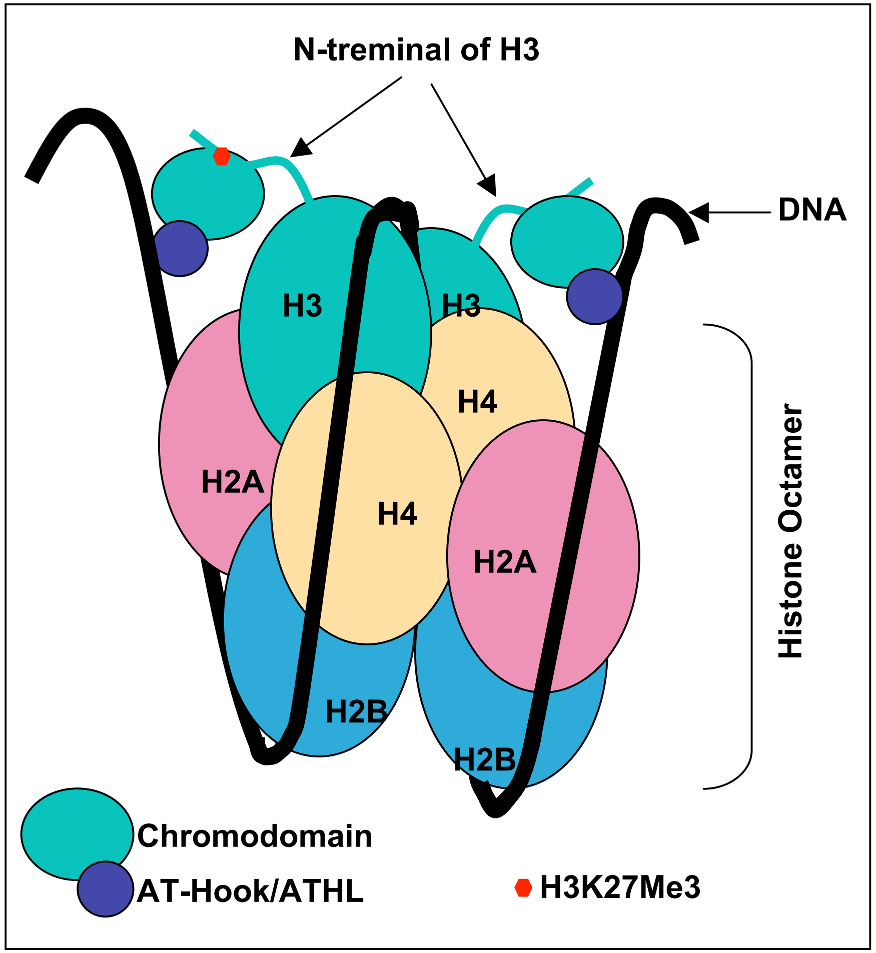

AT-Hook/ATHL motif is shown to interact with DNA. Chromodomain adjacent to the DNA binding domain interacts with the histone H3K27Me3. This three-way interaction locks the nucleosome disallowing any remodelling or dissociation till *Polycomb* (alone or as part of PRC1) is dislodged.

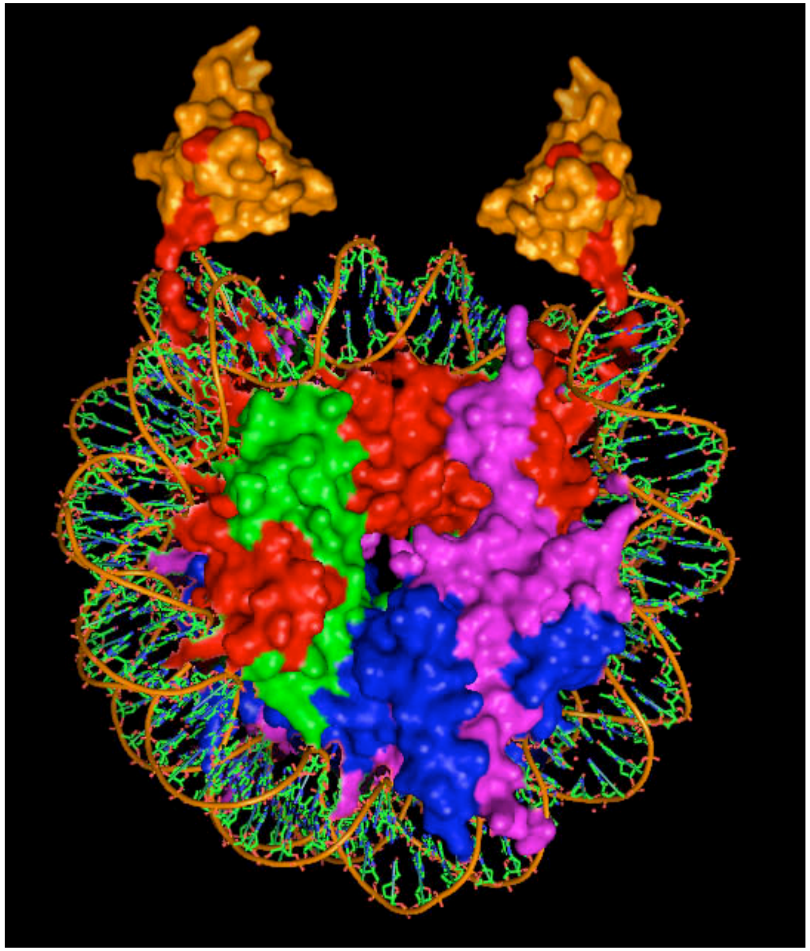

Depiction of chormodomain interacting with the N-terminal tail of Histone H3 on the structure of nucleosome. Proximity of the chromodomain to the minor groove of DNA offers site of interaction with the AT-Hook/ATHL motif as shown in the left side panel.
