# Supplementary material for: Novel motifs distinguish multiple homologues of Polycomb in vertebrates: expansion and diversification of the epigenetic toolkit
Source: BMC Genomics. 2009 Nov 20;10:549. doi: 10.1186/1471-2164-10-549 (PMC2784810; doi:10.1186/1471-2164-10-549)
Supplement: Additional file 9 — List of genome databases searched for mining the homologues. List shows name of the organism and genome database source used in this study to look for PC homologues. [file 1471-2164-10-549-S9.PDF]

Additional file 9 - List of genome databases searched for mining the homologues

Organism	Common name	Source
Homo sapiens	Human	NCBI
Mus musculus	Mouse	NCBI
Rattus norvegicus	Rat	NCBI
Pan troglodytes	Chimp	NCBI
Canis familiaris	Dog	NCBI
Bos taurus	Cow	NCBI
Macaca mulatta	Rhesus macaque	NCBI
Monodelphis domestica	Opossum	NCBI
Equus caballus	Horse	NCBI
Ornithorhynchus anatinus	Platypus	NCBI
Gallus gallus	Chicken	NCBI
Xenopus laevis	African clawed frog	NCBI
Xenopus tropicalis	western clawed frog	Xenbase
Tetraodon nigroviridis	Green spotted pufferfish	Tetraodon genome browser (genoscope.cns.fr)
Danio rerio	Zebrafish	NCBI
Takifugu rubripes	Japanese pufferfish	Fugu genome project(fugu-sg.org)
Aedes aegypti	Yellow fever mosquito	NCBI
Anopheles gambiae	African malaria mosquito	NCBI
Apis mellifera	Honey bee	NCBI
Bombyx mori	Silk moth	NCBI
Culex pipiens quinquefasciatus	Southern house mosquito	NCBI
Drosophila ananassae	Fruit fly sps	NCBI
Drosophila erecta	Fruit fly sps	NCBI
Drosophila melanogaster	Fruit fly sps	NCBI
Drosophila persimilis	Fruit fly sps	NCBI
Drosophila pseudoobscura	Fruit fly sps	NCBI
Drosophila sechellia	Fruit fly sps	NCBI
Drosophila simulans	Fruit fly sps	NCBI
Drosophila virilis	Fruit fly sps	NCBI
Drosophila willistoni	Fruit fly sps	NCBI
Drosophila yakuba	Fruit fly sps	NCBI
Pediculus humanus corporis	head louse	NCBI
Hydra magnipapillata	Hydra	Compagen (compagen.zoologie.uni-kiel.de)
Nematostella vectensis	Starlet sea anemone	Compagen (compagen.zoologie.uni-kiel.de)
Strongylocentrotus purpuratus	Purple sea urchin	NCBI
